# Preserved higher-order mentalising during strategic interactions after medial prefrontal cortex damage

**DOI:** 10.1038/s41467-026-77235-3

**Published:** 2026-10-10

**Authors:** Yi-Chen Hsieh, Arkady Konovalov, Zhi-Lin Su, Todd A. Vogel, Sanjay G. Manohar, Matthew A. J. Apps, Masud Husain, Christian C. Ruff, Patricia L. Lockwood

**Affiliations:** 1 Centre for Human Brain Health, School of Psychology, University of Birmingham, Birmingham, UK; 2 Zurich Center for Neuroeconomics, Department of Economics, University of Zurich, Zürich, Switzerland; 3 Department of Experimental Psychology, University of Oxford, Oxford, UK; 4 Nuffield Department of Clinical Neurosciences, University of Oxford, Oxford, UK; 5 Department of Neurology, John Radcliffe Hospital, Oxford, UK; 6 Wellcome Centre for Integrative Neuroimaging, University of Oxford, Oxford, UK; 7 Institute for Mental Health, School of Psychology, University of Birmingham, Birmingham, UK; 8 University Research Priority Program “Brain Network Mechanisms in Learning and Development” (URPP AdaBD), University of Zurich, Zürich, Switzerland; 9 Faculty of Medicine, University of Zurich, Zürich, Switzerland

**Keywords:** Social neuroscience, Human behaviour

## Abstract

Mentalising, the ability to infer the beliefs and intentions of others, is essential for successful social interaction. Previous studies have implicated the medial prefrontal cortex in mentalising, but whether this region is necessary for higher-order mentalising remains unclear. Here we show that people with focal damage to the medial prefrontal cortex retain the ability to engage in higher-order mentalising during a strategic social game. We compare their performance with patients with brain damage in other regions and lesion-free participants. Individuals with medial prefrontal cortex damage achieve similar rewards and use comparable mentalising strategies, but they are slower when making strategic decisions. Computational modelling indicates that this slowing reflects a greater tendency to require evidence before committing to a choice. Voxel-based lesion-symptom mapping identifies medial prefrontal regions linked to altered decision processes rather than mentalising. These findings suggest that the medial prefrontal cortex supports strategic decisions while higher-order mentalising remains intact.

## Abstract (web summary)

Mentalising is essential for social interaction and is thought to rely on the medial prefrontal cortex. Here, the authors show that damage to the medial prefrontal cortex leaves mentalising intact but alters how quickly people make social decisions.

## Introduction

Successful social interactions frequently rely on our capacity to discern the intentions and beliefs of others, also known as Theory of Mind (ToM) or mentalisation. This ability enables individuals to predict and interpret the behaviour of others in various contexts, ranging from everyday conversations to complex strategic interactions^1–6^. ToM has been linked to multiple aspects of social cognition and decision-making, including cooperation^4^ and empathy^7,8^. However, ToM also plays a crucial role in competitive settings, where anticipating an opponent’s actions can provide a strategic advantage^9–11^.

Several neuroimaging studies in healthy individuals have suggested that ToM relies on a network of brain regions that work together to support mental state attribution^12–21^. These include the temporoparietal junction (TPJ)^22–30^, temporal pole (TP)/superior temporal sulcus (STS)^15,22,24,27,31^, different portions of the anterior cingulate cortex^12,15,28,32^, and several regions of the broader medial prefrontal cortex (mPFC), including dorsomedial and ventromedial mPFC (dmPFC/vmPFC)^9,14,22,24,26,29,31,33–37^. However, findings have not always been consistent across studies, with some suggesting that different regions may support distinct underlying mechanisms^16–18,20,38^. For example, while the TPJ has been implicated in tracking and coding others’ beliefs^24,25^, the temporal poles contribute to belief-based inferences by integrating multimodal information to infer others’ thoughts and emotions in specific contexts^14,24^.

Within the mPFC, there is considerable disagreement regarding the subregions causally involved in ToM and those that are simply correlated with ToM ability. An important case study of a patient with extensive bilateral medial frontal lobe damage showed that their ToM, as measured by pictures, animations, and stories, was surprisingly intact^39^. Several subsequent studies have tested ToM ability using similar measures and reported both preserved and impaired ToM^40^. However, most of these studies used small samples of 15 patients or fewer. Given significant individual differences in ToM ability, larger samples are needed. Moreover, an important aspect of ToM is the capacity to use ToM for successful dynamic social interactions rather than deciphering people’s intentions from stories or cartoons. Finally, whereas fMRI studies provide relatively high-resolution information about which areas of the mPFC are associated with ToM, existing lesion studies have relied on group comparisons with limited ability to identify mPFC subregions involved or to disentangle ToM-specific impairments from more general lesion-related effects on cognition^41,42^. New developments in lesion-symptom mapping^43^ that can be applied to larger patient samples may therefore be necessary to determine whether specific regions are causally implicated in ToM.

More recently, dynamic strategic paradigms have been proposed to measure ToM during actual social interactions involving two or more people. These have shown that the mPFC may track key aspects of ToM, including perspective-taking^10,26^, belief updating^36,44,45^, and social reasoning^9,37,46–50^. Another key advantage is that these online measures of social cognition, where people make decisions repeatedly over multiple trials, can be combined with computational models of behaviour^28,51–53^ to reveal the underlying mechanisms, such as belief updating. Crucially, computational models allow behaviour to be decomposed into latent processes, separating belief-related computations from more general decision dynamics that may also be affected by brain lesions. The participants’ individual parameters from these models can then be used jointly with lesion-symptom mapping to identify specific regions that map onto these computational mechanisms^54–56^.

**Fig. 1:**
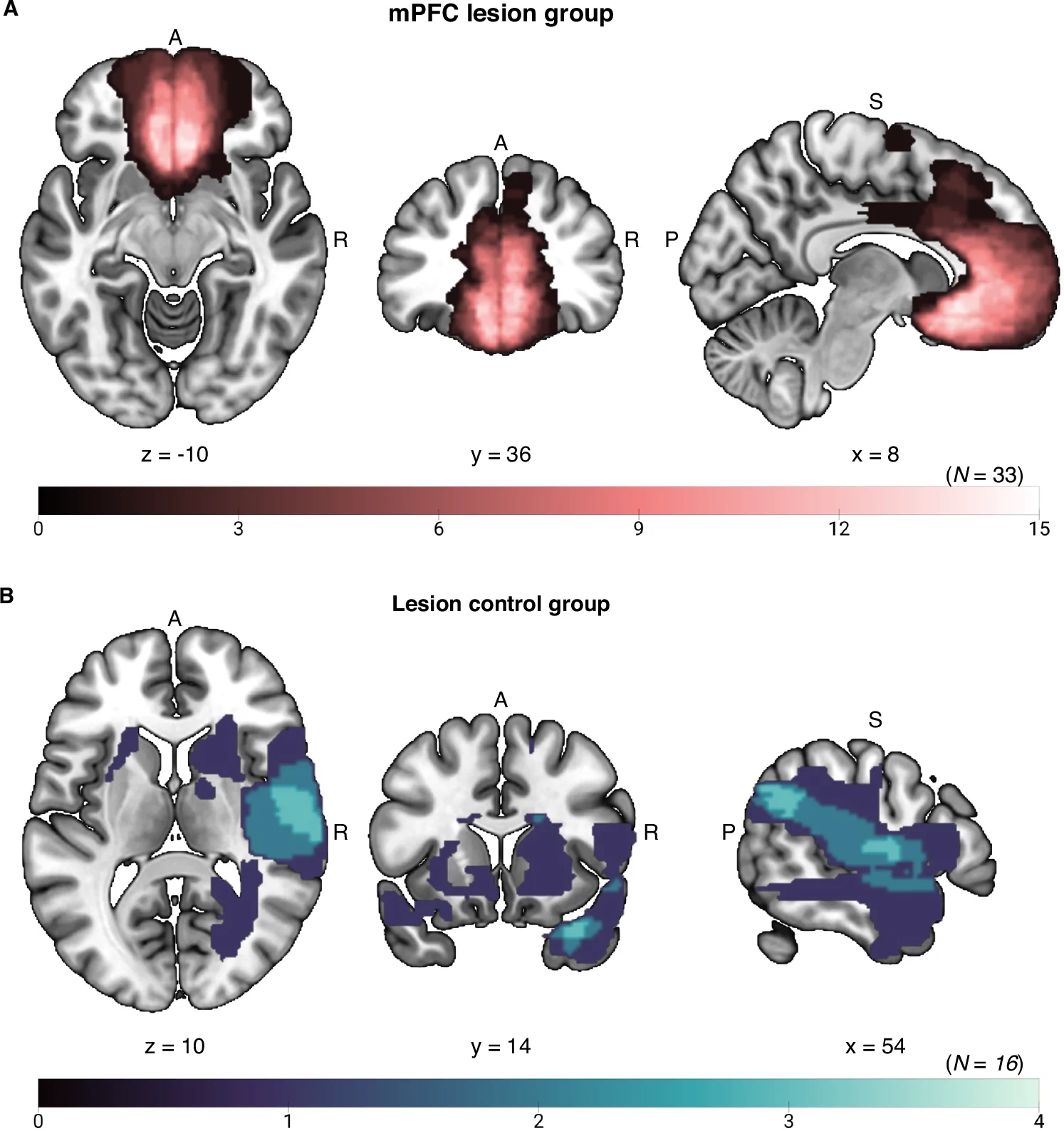
Lesion locations of mPFC lesion patients and lesion control patients. **A** Patients in the mPFC lesion group (*n* = 33) exhibited focal damage to the medial prefrontal cortex (mPFC), with lesions extending into the lateral sections (area 13) and encompassing medial surface subregions (areas 9, 14, 25, and 32) of both hemispheres. **B** The lesion control group (*n* = 16) also sustained damage due to subarachnoid haemorrhage, but to parts of the brain not including the mPFC. Note that three patients in the lesion control group had damage covering the temporal parietal junction. We therefore analysed all results with and without these patients in the lesion control group, and results remained substantively the same despite reduced power (see Supplementary Notes, Supplementary Fig. S12, and Supplementary Table S13). We also conducted additional control analyses in a subgroup of mPFC patients with bilateral rather than unilateral lesions, and again, results remained substantively the same despite reduced power (see Supplementary Notes, Supplementary Fig. S13, and Supplementary Table S14). A: Anterior, R: Right, S: Superior, P: Posterior; x, y, and z: x-, y-, and z-axis; *N*: Number of patients.

Here, we leveraged these advances in model-based lesion-symptom mapping and studied a relatively large sample of patients with focal mPFC damage (*n* = 33, Fig. 1A) as they played a social strategic interaction game that required ToM^26,45^. We compared performance on this task in the mPFC damage group to two closely matched control groups, a sample of healthy age- and gender-matched controls (*n* = 73) and a sample of patients who also had lesions that were outside of the mPFC (*n* = 16, Fig. 1B). The lesion control group was included to control for the general consequences of experiencing a focal brain lesion and the ensuing medical treatment, while avoiding systematic damage to any single non-mPFC region^57^. Given that several studies have linked the mPFC to other cognitive processes such as reaction time latency^58,59^, we built a computational model that combined choice (drift-diffusion) and mentalising (belief-updating) processes. Drift–diffusion models (DDM) use both choices and response times to estimate how fast people accumulate evidence during a choice, their threshold for initiating a response, and the time taken for non-decision processes. This allowed us to simultaneously assess a range of latent processes guiding behaviour, thereby providing insight into the mechanisms required to successfully interact with others and elucidating the specific behavioural deficits caused by mPFC damage.

Our findings show that patients with mPFC damage have preserved mentalisation during strategic interactions with others: they earn as many credits as healthy individuals and lesion controls, and computational modelling reveals that they are able to strategically influence other players to a similar degree. Self-report additionally showed that those with mPFC damage strategically used mentalising strategies to the same extent as healthy controls.

Intriguingly, mPFC damage led to a specific slowing when making strategic decisions, which drift-diffusion modelling (DDM) attributed to more cautious responding (a higher boundary in accumulating evidence) rather than to general slowing of perception and motor planning (non-decision processes). Lesion-symptom mapping further demonstrated that no area of mPFC was related to mentalising as quantified by using strategic influence play (which requires ToM), whereas damage to several regions of the mPFC was associated with aspects of response speed, including the drift rate, decision boundary, and non-decision time. Together, these findings suggest that while the mPFC is not necessary for successful mentalisation during strategic interactions, subregions of the mPFC may be crucial for the speed of social decision-making. These results suggest that caution may be warranted when interpreting ToM-related measures in neuroimaging studies and in patients with mPFC damage in future studies.

## Results

**Fig. 2:**
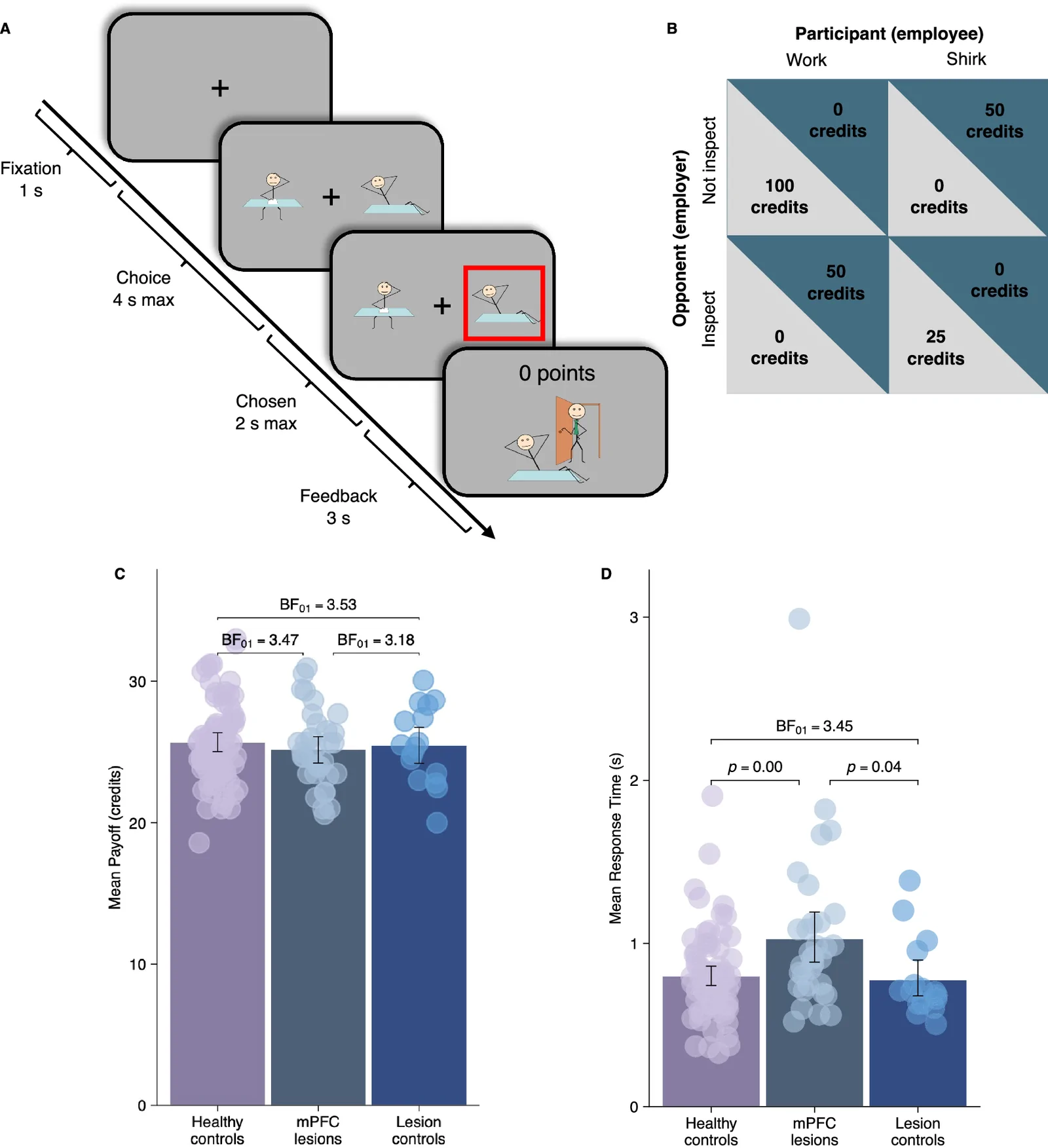
The inspection game design and game performance. **A** In the strategic mentalising inspection game, participants were instructed to choose between work and shirk for 150 trials as employees. They were told that they would play against another participant they had anonymously met at the beginning, who had been allocated the role of employer. For experimental control, the employer was in fact a computer algorithm modelled to behave like another player (See Methods for details and Supplementary Fig. S1 for role-assignment procedure^60–63^). Participants were informed on each trial to choose between working and not working. The red square would then mark the chosen option, followed by the result. The outcome was instructive about the participant’s decision, their opponents’ choice, and the points they earned on that trial. **B** The points shown on the outcome page were computed based on the payoff matrix and were converted into a monetary bonus payment. **C**, **D** There was strong Bayesian evidence to support no difference in overall task payoff between the three groups (BF_01_ = 7.98). Pairwise Bayesian* t* tests are shown for descriptive comparison (HC vs mPFC: BF_01_ = 3.47; mPFC vs LC: BF_01_ = 3.18; HC vs LC: BF_01_ = 3.53). In contrast, those with mPFC damage had longer response times compared to both control groups, suggesting that although they could effectively use mentalising, they were consistently slower to do so. Group differences were assessed using a one-way ANOVA (two-sided) together with Bayesian ANOVA and Bayesian *t* tests. Each coloured dot represents an individual participant (HC: *n* = 73; mPFC: *n* = 33; LC: *n* = 16). Bars indicate group means, and error bars represent 95% bootstrap confidence intervals. *p* = *p*-value, BF_01_ = Bayes factor for the null hypothesis. See Supplementary Fis. S2, S3 for the accumulated payoff and RTs distribution; see Supplementary Table S2–S10 for additional control and robustness analyses. Panel A was adapted from Hill et al. (2017)^26^, in accordance with Springer Nature’s author reuse policy.

To examine the causal role of the mPFC in mentalising during strategic interactions, we tested 33 participants with rare focal mPFC damage (mean age: 56.88; 17 females, Fig. 1A), 16 lesion control patients (mean age: 56.06; 11 females, Fig. 1B), and 73 healthy control participants (mean age: 60.53; 38 females). The three groups were matched on age, gender, and education, with no significant difference in cognitive ability and visual attention. In addition, the two lesion groups had no significant difference in cognitive function, depressive symptoms, or apathy levels (Supplementary Table S1; see Methods and Supplementary Methods). All participants completed the inspection game task^26,45^. In this game, two participants are told they play a game where one of them is assigned to be an employer and the other an employee. In the role of the employee, they earn credits (converted into money) in two scenarios: if they work and the other participant inspects what they are doing, or if they shirk and the other participant does not inspect. However, if they unnecessarily work and the other player does not inspect, or if they shirk and the other player inspects, they do not receive any credits (Fig. 2A, B). To successfully play the game, the best strategy, termed influence play, is to pick choices that influence what the other person will do; specifically, alternating one’s choices is a marker of using ToM. Other strategies include simply using reinforcement learning with no ToM or using fictitious play, a more basic strategy that only tracks the opponent’s past actions. We introduced participants to the other player (a confederate) while hiding their identity before they played the game^60–63^ (see Supplementary Methods and Supplementary Fig. S1). All participants played the role of the employee, with an algorithm (which combined fictitious and influence play) serving as the employer to ensure experimental control across participants. No participant reported disbelief that the algorithm was not another person.

### Patients with brain damage demonstrate preserved performance but slower response times in strategic social interactions

We first analysed whether the three groups differed in overall performance. We found that there was moderate evidence for no difference in total reward received between the three groups (Fig. 2C; one-way ANOVA: *F*_*(2,119)*_ = 0.313, *p* = 0.732, η² = 0.005; Bayesian ANOVA: BF_01_ = 7.98; see cumulative payoff trajectories across rounds for each group in Supplementary Fig. S2). However, patients with mPFC lesions exhibited longer response times compared to the other two groups (Fig. 2D; one-way ANOVA: F_(2,119)_ = 5.796, *p* = 0.004, η² = 0.090, 95% CI [0.02, 1.00], BF_10_ = 10.23; mPFC vs HC: diff [95% CI] = 0.23 [0.06 0.40], *p* = 0.004, BF_10_ = 14.75, mPFC vs LC: diff [95% CI] = 0.25 [0.50 0.01], *p* = 0.040, BF_10_ = 1.41), whereas healthy controls and lesion controls did not differ (LC vs HC: diff [95% CI] = −0.02 [−0.24 0.20], *p* = 0.963, BF_01_ = 3.45; see participants’ RT distribution in the Supplementary Fig. S3). Therefore, although mPFC damage was associated with slower response times, performance remained intact. This pattern remained unchanged when controlling for individual differences in general cognitive ability and non-social processing speed, as indexed by Raven Standard Progressive Matrices and Trail Making Test performance (see Supplementary Notes, Supplementary Tables S9, S10). Additional robustness analyses examining the distribution of response times, censoring trials and individuals with extreme RTs, log-transforming RTs, and using a mixed-effects model are reported in the Supplementary Notes (Supplementary Tables S2–S6) and confirm that the RT effects are robust. Complementary analyses of trial-wise RT changes and practice-phase performance indicate that these effects do not reflect slower task adaptation or delayed task understanding (see Supplementary Notes, Supplementary Table S7, S8).

### Separating mentalising from basic choice mechanisms and perceptual and motor speed using computational modelling

Having shown that mPFC damage leads to slower decisions but does not alter the ability to gain reward during strategic interactions, it is crucial to identify the cognitive strategy participants used and the learning or choice mechanisms that are affected by the brain damage. Computational models can reveal latent factors driving participants’ accuracy and response times that are not evident from summary statistics alone. We therefore developed a combined drift-diffusion decision and learning model to capture the mechanisms underlying accuracy and response times within a single model. Previous learning models of mentalising have shown that the degree to which humans exhibit influence play, the most advanced type of mentalising in the inspection game, can be modelled by a parameter ${{{\rm{\kappa }}}}$ (second-order belief weight). This parameter quantifies how strongly people update their beliefs about how the other person reacts to their own choices, in other words, their mental model of how their opponent’s beliefs are shaped by their own choices. In addition to this index of mentalising, the model captures lower-level observational learning, or fictitious play, with a parameter ${{{\rm{\eta }}}}$ (first-order belief weight), quantifying how strongly people track the opponent’s probability of selecting a particular action, irrespective of any concerns about the opponent’s belief. In addition to these parameters, capturing the extent of influence and fictitious play, the DDM choice component modelled drift rate multiplier d (quantifying sensitivity to evidence for choosing one specific action), decision boundary ${{{\rm{\alpha }}}}$ (quantifying response caution, i.e., the amount of evidence required before committing to a choice), and non-decision time ${{{\rm{\tau }}}}$ (quantifying the constant speed of perceptual processing before the choice process and/or of motor initiation). We compared this influence-DDM model to three other plausible models, again established in previous work (1) a standard reinforcement learning model combined with the DDM (RL-DDM), (2) a fictitious play DDM model, and (3) a simple influence DDM model that only takes one previous trial into account (see Methods and Supplementary Methods).

**Fig. 3:**
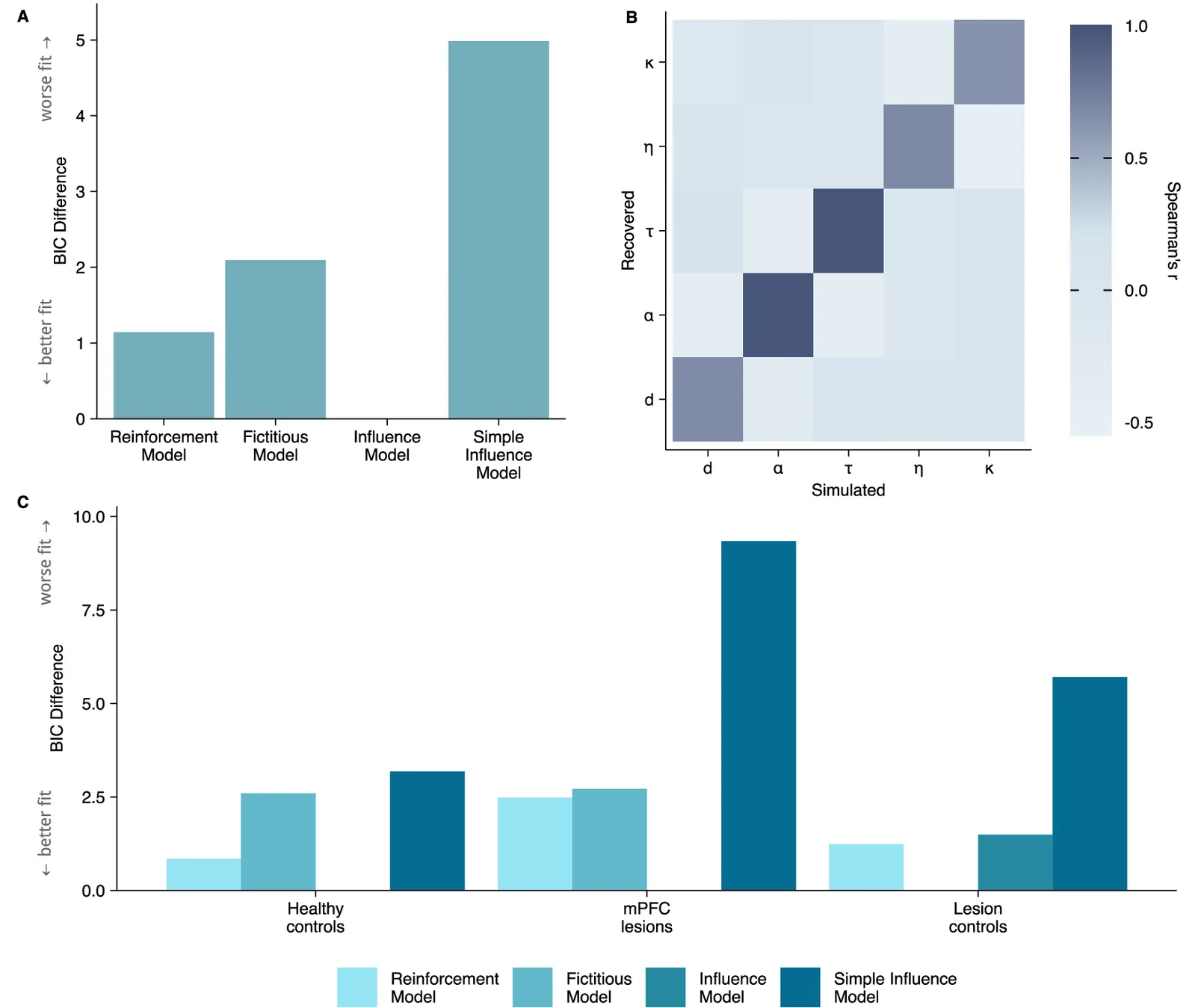
The influence-DDM model best accounts for participants’ behaviour. **A** The Bayesian Information Criterion (BIC) value was calculated for each participant and model, averaged for each model, and plotted after subtracting the lowest average BIC value associated with the winning influence-DDM model. Thus, the best-fit influence-DDM model has a zero BIC difference value, and the worst-fit model has the highest value. **B** The winning model showed excellent parameter recovery, as evidenced by the high correlations on the diagonal and the low correlations off the diagonal (see Supplementary Notes and Supplementary Fig. S5 for model validation). **C** BIC difference of each group (see Supplementary Fig. S4 for the plot with individual data points overlaid; see Supplementary Fig. S6 for additional predictive accuracy comparisons within the LC group). Also see Supplementary Fig. S7 for illustrative trial sequence examples and Supplementary Fig. S8 for the parameter correlation matrix.

The influence-DDM model was best able to explain behaviour across groups, showing the lowest Bayesian Information Criterion (BIC) score (mean BIC: RL-DDM = 307.42, Fictitious-DDM = 308.37, Influence-DDM = 306.28, Simple Influence-DDM = 311.27) (Fig. 3A; see Fig. 3C and Supplementary Fig. S4 for across-groups BIC with and without individual data points; see also Supplementary Fig. S7 for illustrative example trial sequences). Although inspection of group-wise BIC values indicated that the lesion control (LC) group showed a marginal preference for the simpler fictitious-DDM, this difference was small and was not supported by predictive accuracy analyses (see Supplementary Notes and Supplementary Fig. S6). The model had excellent parameter recovery (Fig. 3B) and could accurately reconstruct participants’ choices (see Supplementary Notes and Supplementary Fig. S5). To assess the independence of model parameters, we examined correlations across all five parameters and tested their multicollinearity. These analyses revealed minimal collinearity (all VIFs ≈ 1.01; see Supplementary Notes and Supplementary Fig. S8), indicating that the parameters capture largely independent aspects of behaviour. Therefore, a computational model that incorporated mentalising was best able to describe participants’ behaviour across groups, which again supports that mPFC damage does not seem to impair mentalising ability.

### mPFC damage preserves mentalising and leads to a higher boundary of evidence accumulation

**Fig. 4:**
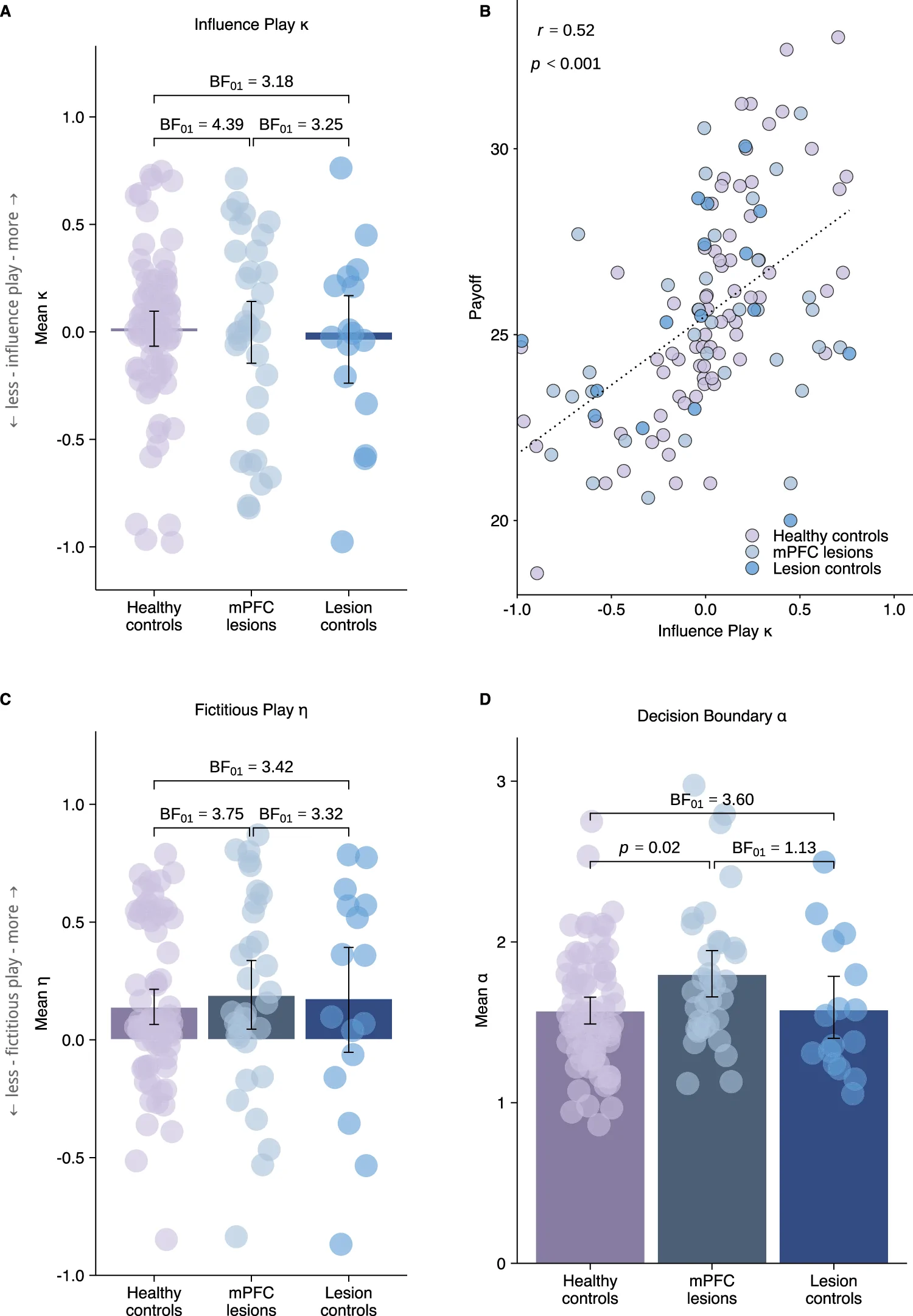
Patients with mPFC damage display preserved influence and fictitious play but a higher decision boundary. **A** The mPFC patients still engage in influence play with strong Bayesian evidence for no difference between groups. **B** Across participants, those who use more influence play gain higher payoffs (two-sided Pearson correlation, *r*_(120)_ = 0.52, 95% CI [0.37, 0.64], *p* = 1.17 × 10⁻⁹), highlighting that using influence play is the optimal strategy. **C** The mPFC patients still engage in fictitious play with strong Bayesian evidence for no difference between groups. **D** The mPFC patients had a higher decision boundary than healthy controls, with anecdotal evidence for a difference from lesion controls (see Supplementary Fig. S9 for the other two DDM parameters, Supplementary Fig. S10 for analysis of decision boundary and payoff, Supplementary Fig. S11 for exploratory analysis, and Supplementary Figs. S12, S13 for control analyses). Group differences were assessed using a one-way ANOVA (two-sided) followed by Tukey-adjusted post hoc comparisons, together with Bayesian ANOVA and Bayesian *t* tests. Each coloured dot represents an individual participant (HC: *n* = 73; mPFC: *n* = 33; LC: *n* = 16). Bars indicate group means, and error bars represent 95% bootstrap confidence intervals. *p* = *p*-value, BF_01 _= Bayes factor for null hypothesis.

Next, we identified whether mPFC damage was associated with differences in latent processes captured by the best-fitting influence-DDM model. We found that higher-level mentalising, as captured by the influence play parameter from the computational model, was preserved after medial prefrontal damage, as well as after damage elsewhere (Fig. 4A, influence play parameter ${{{\rm{\kappa }}}}$: *F*_*(2,119)*_ = 0.15, *p* = 0.861, η² = 0.003, BF_01_ = 9.24; mPFC vs HC: diff [95% CI] = − 0.02 [− 0.22 0.18], *p* = 0.957, BF_01_ = 4.39; LC vs HC: diff [95% CI] = − 0.06 [− 0.32 0.20], *p* = 0.860, BF_01_ = 3.18; LC vs mPFC: diff [95% CI] = 0.03 [− 0.32 0.25], *p* = 0.957, BF_01_ = 3.25). We then checked whether exhibiting more influence play, i.e., more mentalising, in the task resulted in higher performance. We observed that across groups, using more influence play was indeed associated with greater reward (Fig. 4B, r_*(120)*_ = 0.52, *p* = 1.17e-09, 95% CI [0.37, 0.64]), making it the optimal strategy (as intended by the opponent algorithm design).

We also observed no significant difference between the groups in the level of fictitious play ${{{\rm{\eta }}}}$ (Fig. 4C*; F*_*(2,119)*_ = 0.22, *p* = 0.803, η² = 0.004, BF_01_ = 8.62; mPFC vs HC: diff [95% CI] = − 0.05 [− 0.14 0.24], *p* = 0.803, BF_01_ = 3.75; LC vs HC: diff [95% CI] = − 0.04 [− 0.21 0.28], *p* = 0.936, BF_01_ = 3.42; LC vs mPFC: diff [95% CI] = − 0.01 [− 0.29 0.26], *p* = 0.992, BF_01_ = 3.32). In contrast, mPFC patients showed a significantly higher decision boundary ${{{\rm{\alpha }}}}$ compared to healthy controls (Fig. 4D; F_*(2,119)*_ = 3.93, *p* = 0.022, η² = 0.062, BF_10_ = 2.40; mPFC vs HC: diff [95% CI] = 0.23 [0.03 0.42], *p* = 0.019, BF_10_ = 5.92; with weak evidence showing no difference from lesion controls: LC vs mPFC: diff [95% CI] = − 0.22 [− 0.51 0.07], *p* = 0.163, BF_01_ = 1.13; and moderate evidence of no difference between healthy and lesion controls: LC vs HC: diff [95% CI] = − 0.01 [− 0.25 0.26], *p* = 0.998, BF_01_ = 3.60). The other two DDM parameters (drift rate temperature d and non-decision time ${{{\rm{\tau }}}}$) did not significantly differ between groups (see Supplementary Notes and Supplementary Fig. S9). These results remained when controlling for several cognitive measurements (see Supplementary Notes and Supplementary Table S11). Although simulations indicated that higher decision boundaries can, in principle, increase payoff, no direct association between fitted decision boundary and payoff was observed in the empirical data, where behaviour reflects the joint influence of multiple model parameters (see Supplementary Notes, Supplementary Fig. S10, and Supplementary Table S12). These findings suggest that mPFC patients need more evidence before committing to a decision, aligning with the observed response time differences between the groups, and that all three groups processed the perceptual information similarly and were equally sensitive to evidence for one choice.

Finally, we conducted two sets of control analyses. First, we re-ran analyses excluding patients in our lesion control group (*n* = 3) who had damage covering the right TPJ (remaining LC sample *n* = 13), and results remained substantively the same (see Supplementary Notes, Supplementary Fig. S12, and Supplementary Table S13*)*. Second, we re-ran our analyses, including only those patients with bilateral or partially bilateral mPFC lesions (*n* = 17), excluding all unilateral cases, to test whether preserved mentalising could be accounted for by the intact contralateral hemisphere. Again, results remained substantively the same (see Supplementary Notes, Supplementary Fig. S13, and Supplementary Table S14*)*. We also ran an additional exploratory analysis to examine how RT latencies reflected belief updating from the computational model between groups. This additional analysis suggested participants with mPFC lesions tended to exhibit larger second-order belief updates on faster-response trials, a pattern that was consistent across alternative model specifications (see Supplementary Notes, Supplementary Fig. S11, and Supplementary Table S15*)*.

### Damage to mPFC subregions impacts boundary, drift rate temperature, and non-decision time

Our behavioural analysis showed that mPFC damage was associated with preserved mentalising but slower decision-making during strategic interactions, captured in our model by higher boundaries in the evidence accumulation process. We next sought to examine whether there were specific brain regions in which damage was associated with more fine-grained differences in the extent of influence play, fictitious play, or the DDM choice parameters. Given our relatively large sample of brain-lesion patients (*N* = 49), we used voxel-based lesion-symptom mapping (VLSM), which tests whether voxel-level damage predicts variability in model-derived parameters across patients, by generating a brain t-statistics map that is independent of group-level differences. The VLSM was conducted using the permutation-based threshold-free cluster enhancement (TFCE)^64,65^ implemented in the FMRIB Software Library (FSL) (see Methods and Supplementary Methods).

In this whole-brain analysis, we included all lesion patients (the mPFC lesion group and the lesion control group). This approach tests whether damage to a given voxel accounts for parameter variability beyond the general effects of a brain lesion. To ensure stable voxel-wise estimates, voxels damaged in fewer than five patients were classified as intact rather than lesioned. The analysis, therefore, provides a conservative assessment of voxel-level lesion–behaviour associations across the brain. Using a single unified analysis across all five model parameters (significance was reported at permutation-based TFCE *p* < 0.01, Bonferroni-corrected across five regressors), we first examined strategy-related parameters. Neither the influence play nor the fictitious play parameters were significantly correlated with any areas of damage.

**Fig. 5:**
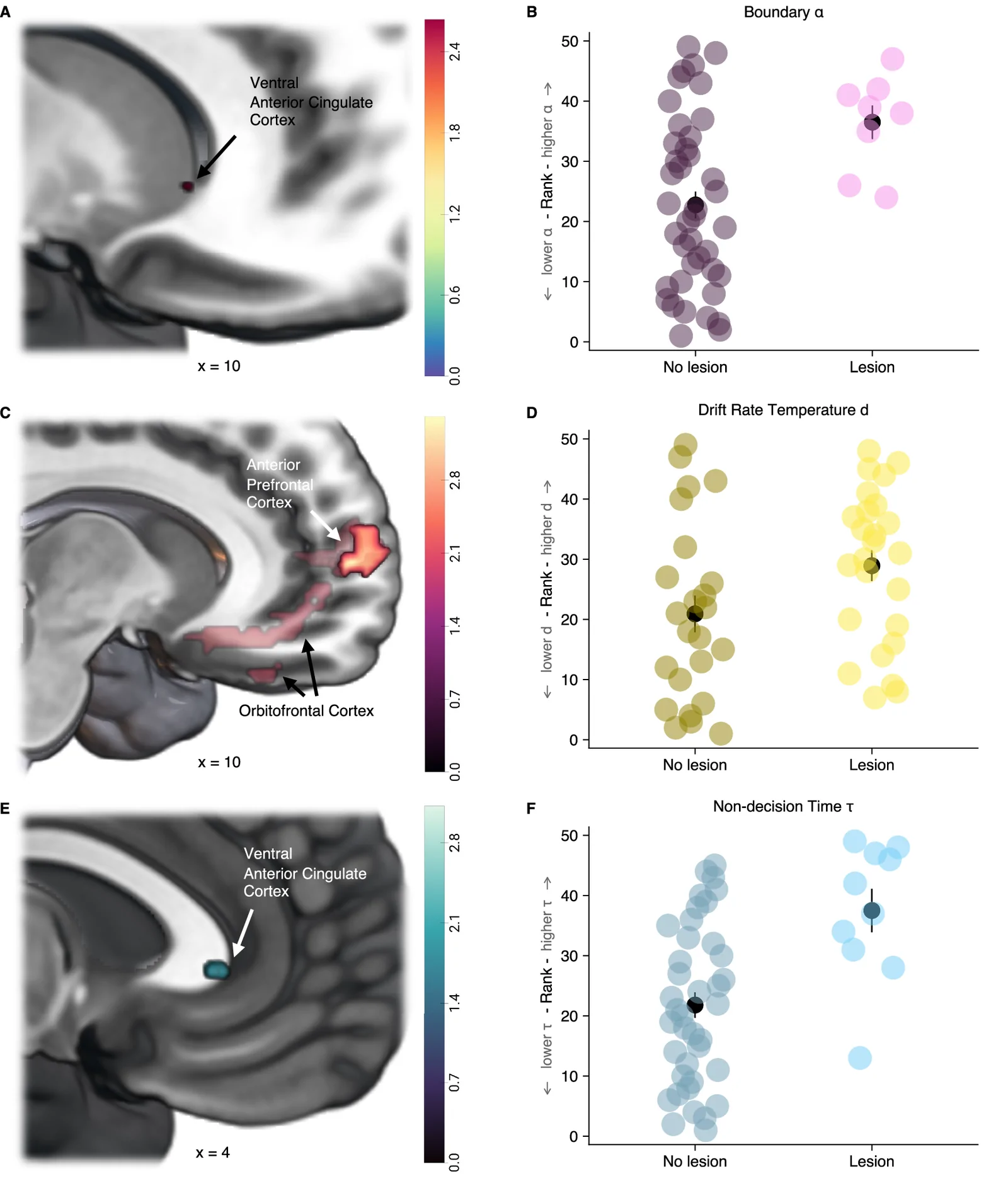
Damage to the orbitofrontal cortex (OFC), anterior prefrontal cortex (aPFC), and ventral anterior cingulate cortex (vACC) was associated with the DDM parameters. Permutation-based, whole-brain, non-parametric voxel-based lesion-symptom mapping (VLSM) results of (**A**) decision boundary ${{{\rm{\alpha }}}}$, (**C**) drift rate temperature d, and (**E**) non-decision time ${{{\rm{\tau }}}}$. The ranked (**B**) boundary ${{{\rm{\alpha }}}}$ (no lesio*n*: *n* = 41; lesio*n*: *n* = 8), (**D**) temperature d (no lesion: *n* = 24; lesion: *n* = 25), and (**F**) non-decision time ${{{\rm{\tau }}}}$ (no lesion: *n* = 39; lesion: *n* = 10) contrasts of participants with damage or no damage in the areas identified by the VLSM analysis (see Supplementary Fig. S15 for mPFC patients only analysis). *p* < 0.01 (the colour bar represents the *t*-value of the analysis). Note: Panels (**B**), (**D**), and (**F**) are for illustrative purposes only and display the ranked difference in model parameter contrasts between participants with vs. without lesions in the ROI defined by a whole-brain contrast (see Supplementary Fig. S14 for lesion volume correlations) Each coloured dot represents an individual participant (included patients with mPFC lesion, *n* = 33, and patients with lesion elsewhere, *n* = 16). Black dots indicate group rank means, and error bars indicate mean ± SEM.

We next examined decision-related DDM parameters. We observed that damage to specific mPFC subregions was associated with a higher boundary ${{{\rm{\alpha }}}}$. Lesions involving the ventral anterior cingulate cortex (vACC) compared to damage elsewhere showed this pattern (Fig. 5A, B; peak MNI coordinate [– 4,10,24], cluster size k = 2). Damage to the orbitofrontal cortex (OFC), extending to anterior prefrontal cortex (aPFC), resulted in a higher drift rate parameter ${{{\rm{d}}}}$ compared to damage elsewhere (Fig. 5C, D; peak MNI coordinate [7,10,62], cluster size *k* = 1700; peak MNI coordinate [– 14,1,36], cluster size *k* = 526; peak MNI coordinate [– 27,2,36], cluster size *k* = 526). Finally, participants with lesions in the vACC also displayed a higher non-decision time ${{{\rm{\tau }}}}$ (Fig. 5E, F**;** peak MNI coordinate [– 1,4,32], cluster size *k* = 42).

To further examine whether variability within the mPFC itself was associated with these parameters, we conducted a complementary VLSM analysis restricted to the focal mPFC lesion group. This mPFC-only analysis tests whether differences in lesion location within the mPFC predict parameter variability, independent of damage outside this region. Results and the discussion from this within-region analysis are presented in the Supplementary Notes and Supplementary Fig. S15.

Together, these analyses indicate that while higher-order mentalising parameter (influence-play) was not associated with damage to specific mPFC regions, variability in decision-related parameters was linked to damage within the mPFC regions, as revealed by both whole-brain and within-region lesion-mapping approaches. These findings support the mPFC areas as crucial for the latent factors influencing the decision process in general and response times specifically during strategic interactions.

### mPFC patients report use of mentalising and related strategies when interacting with others

**Fig. 6:**
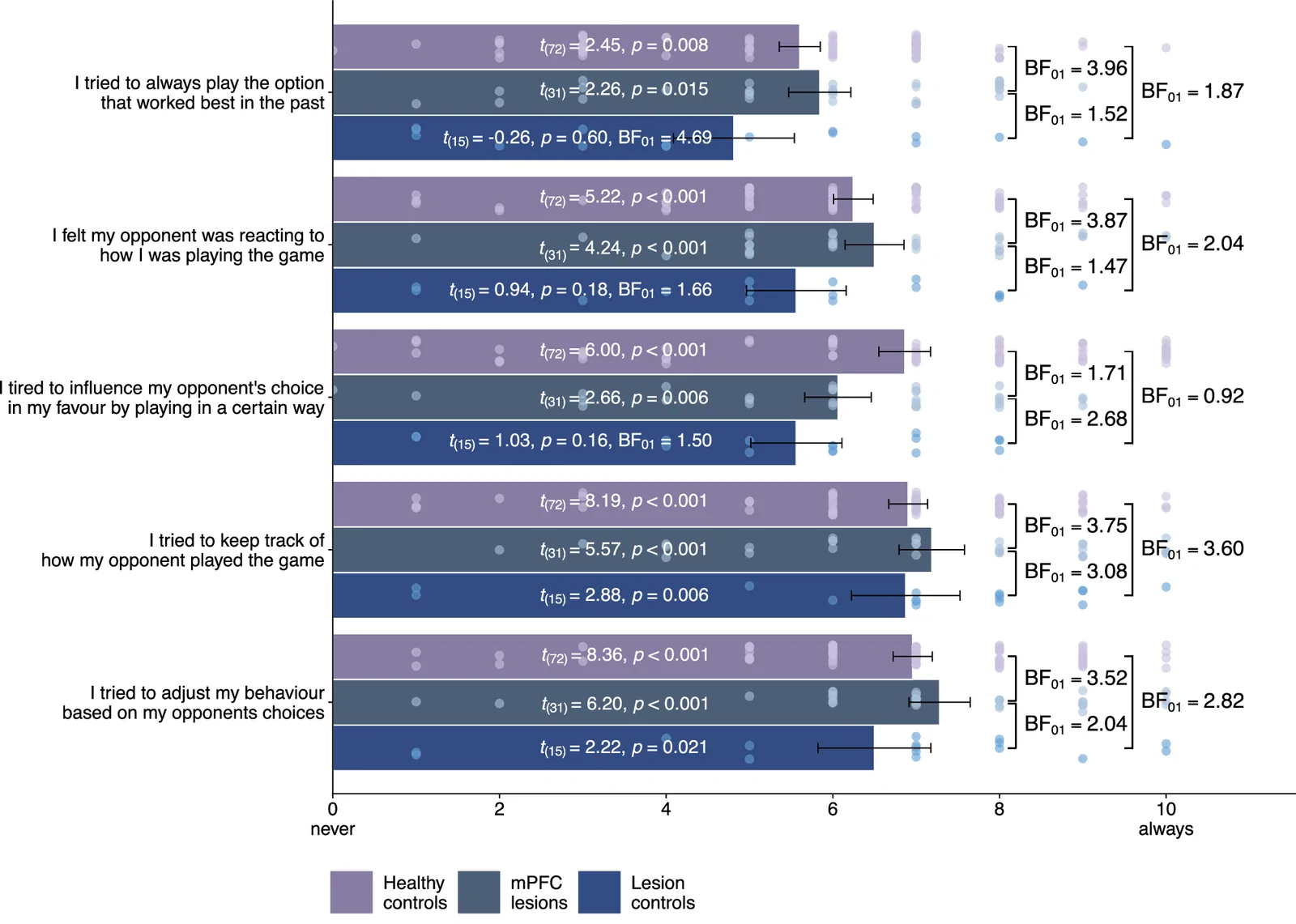
Patients with mPFC damage self-report using mentalising strategies when interacting with others. The strategies participants reported using while playing the inspection game. Participants provided a response on a 0–10 scale for each strategy statement (0 = never, 10 = always). Bayesian evidence showed the preserved use of mentalising strategies when interacting with others in patients with mPFC damage (see Supplementary Table S18, S19 for full statistics). We also assessed whether participants’ subjective strategy-use scores were significantly greater than 5, with the results displayed on the coloured bars (see Supplementary Table S17 for full statistics). Bars represent mean values, and error bars represent SEM. Each coloured dot represents an individual subject (HC: *n* = 72; mPFC: *n* = 32; LC: *n* = 16). Group differences were assessed using one-way ANOVA (two-sided). Differences from the midpoint value (5) were assessed using one-sided one-sample* t* tests. *p* = *p*-value. BF_01 _= Bayes factor for the null hypothesis.

Finally, we asked all participants at the end of the experimental task to report the extent to which they used different strategies when playing the game, to probe whether patients were aware of using particular strategies during their interactions. We asked a series of questions, such as “I tracked what my opponent was doing during the game” and “I tried to influence my opponent’s choice by playing a certain way” (0-10 scale, Fig. 6). Participants with mPFC damage reported using higher-order mentalising (influence play) strategy significantly more than the neutral midpoint (*t*_(31)_ = 2.66, *p* = 0.006; see Supplementary Table S17), and there was no statistically significant difference in the self-reported strategy between any group (see Supplementary Table S18), with Bayesian pairwise evidence ranging from anecdotal to moderate evidence for the null hypothesis (0.92 ≤ BF_01_s ≤ 3.96; see Supplementary Table S19). This shows that mPFC patients not only had preserved mentalising but also preserved insight into the use of the related strategies.

## Discussion

Functional imaging in healthy individuals has shown that activity within the medial prefrontal cortex (mPFC) is associated with mentalising, a process crucial for successful social interaction^8,9,12,21,33–37,47,66–68^. However, whether the mPFC is necessary for mentalising remains unclear^25,39,69^. Here, we used a carefully developed social strategic game that required mentalising in a large rare sample of patients with focal mPFC lesions and compared performance to matched healthy controls and patients with lesions outside of the mPFC. We found that patients with mPFC damage showed a preserved ability to mentalise when interacting with others. However, intriguingly, mPFC patients were significantly slower to mentalise, which was not due to different perceptual or motor abilities but to a heightened threshold for mentalising-related evidence accumulation in the service of reaching a decision. Moreover, lesion mapping showed discrete subregions of mPFC where damage altered other choice-related subprocesses, such as non-decision time and the drift rate of evidence accumulation, but not mentalising computations themselves. Together, these findings suggest that mPFC damage preserves the ability to mentalise during strategic interactions, yet changes the speed at which mentalising decisions are made.

While neuroimaging studies have consistently implicated the mPFC in mentalising processes^9,35,47,50,68,70^, their correlational nature and the signal dropout in ventral regions limit conclusions regarding causality and the accuracy of spatial inferences^71^. In contrast, lesion studies offer a more direct approach to establishing causal links. However, findings from such studies have been inconsistent, with some reporting impairments in mentalising following mPFC damage^72–76^ and others demonstrating preserved or only subtle impairments^39,40,77,78^. Bird and colleagues (2004) suggested that the mPFC may not be necessary for ToM performance in a case study involving a patient with extensive bilateral mPFC damage who retained her ability to perform well within the normal range of healthy individuals on four out of the five ToM tasks. In all previous lesion studies, paradigms required social understanding from stories, cartoons, or faces. Yet whether mPFC damage affects the use of mentalising when strategically interacting with others is unknown. Here, we addressed these limitations by testing a relatively large sample of over 30 individuals with focal mPFC lesions and measuring social behaviour. Notably, the performance of mPFC patients in a social strategic interaction task did not differ from that of healthy controls or lesion controls, suggesting that higher-order mentalising abilities can be preserved despite mPFC damage.

These findings should be interpreted within a multifaceted view of mentalising^79^, rather than as evidence that the mPFC is unnecessary for all forms of social cognition. Contemporary accounts increasingly emphasise that mentalising comprises dissociable components—such as belief-based strategic reasoning, affective understanding, valuation of others, and self-referential processing—that may rely on partially distinct neural substrates^80–83^. The paradigm we used specifically targets belief-based inference about another agent’s likely actions during repeated strategic interaction, a component that may be supported by a distributed social cognition network rather than by the mPFC alone^44,84,85^. From this perspective, preserved performance following mPFC damage may reflect compensation via other regions within this network, either through long-term plastic reorganisation after the lesion or through the flexible recruitment of intact regions during task performance^86^. This aligns with some studies suggesting that a distributed network, including the temporoparietal junction (TPJ)^22–30^, flexibly supports mental state attribution. Among these studies, Hill et al. (2017)^26^ used transcranial magnetic stimulation (TMS) and applied the same inspection game and computational modelling approach to establish causal links between specific brain regions and mentalising processes. The findings highlight the right TPJ (rTPJ) as the core region for higher-order mentalising, particularly the influence-update computation that supports sophisticated strategic behaviour. In contrast, the mPFC was associated with related but distinct functions, including representing others’ strategies and integrating social information into value-based decisions^38^. This functional distinction aligns with our current findings, which show that individuals with mPFC damage can still engage in belief-based reasoning as captured by the same computational model, which incorporates first- and second-order belief and can describe the capacity to mentalise in games.

Although participants with mPFC lesions exhibited preserved mentalising abilities, their response times during social strategic decision-making were significantly longer, indicating altered decision dynamics. Previous research has highlighted the mPFC as a key node responsible for integrating social information^21,31,87–89^ and value-based processing^90–93^, functions that may influence decision latency even in the absence of explicit mentalising requirements. Studies have shown that the vmPFC is involved in the integration of self- and other-related values^94,95^. Similarly, the dmPFC has been associated with the monitoring of others’ intentions in primates^96^, especially when updating strategic models^26,45^. All these processes may increase computational demands during strategic social interactions. While several lesion studies have indicated that mPFC damage can impair the efficiency of strategic decision-making^58,59,97,98^, to our knowledge, none have specifically investigated how such impairments manifest during interactive social contexts. Consequently, our findings provide evidence in this domain, demonstrating that mPFC damage may selectively affect the temporal dynamics of social decision-making, even when mentalising abilities remain intact.

Leveraging computational modelling^99,100^, we employed a novel model that combines learning and the drift-diffusion choice process to elucidate the underlying mechanisms responsible for the social decision latency observed^101–105^. Drift-diffusion modelling raises the possibility that the observed deceleration in participants with mPFC lesions may primarily be attributed to factors such as slower perceptual and motor processing (captured by increased no-decision time) or more cautious decision making as indicated by an increased decision boundary^106,107^. Our findings support the latter, suggesting that individuals with damage to the mPFC may require additional evidence before committing to a strategic choice^107,108^, potentially indicating alterations in decision maintenance or confidence thresholds^109–111^. Conversely, the observed latency is unlikely to reflect a general slowing of perception or impairment in executive function^102,105,107,112–114^, as the non-decision time parameter in the model did not differ between groups, with Bayesian evidence supporting no significant difference. The integrated results imply that the delayed decision-making process is more likely a domain-specific phenomenon. Future studies may examine whether this domain-specificity is for social choices in general, or even more specifically, during strategic choice.

Furthermore, our computational modelling results revealed no differences between groups in either the first-order belief update weight (fictitious play parameter) or the second-order belief update weight (influence play parameter). The absence of group differences in these mentalising-related parameters^26,45^ provides crucial evidence that the mPFC lesion patients are capable of engaging in higher-order mentalising. Despite damage to the mPFC, participants’ choice behaviour could still be best captured by the influence-DDM model, and their use of higher-order belief updating strategies^26^ remained. This preservation of social strategic behaviour stands in striking contrast to well-recognised impairments in broader strategic functions commonly associated with mPFC damage, such as deficits in multi-step planning^115–117^, as well as altered goal-directed reward processing^118^. Moreover, recent lesion studies have implicated the mPFC in processing social influence from others^56^ and prosocial motivation^54^, further suggesting that while the mPFC plays an important role in integrating social information during valuation and social motivation, the computations underpinning social strategic interaction, such as belief-based modelling of others, may constitute a distinct construct. Our findings suggest that this form of social strategy can be preserved even when the mPFC is compromised^11,18,22–24,38,119^.

Due to our relatively large sample, we were able to use voxel-based lesion-symptom mapping^54–56^. Notably, we did not identify any specific subregion within the mPFC that exhibited a predictive role in higher-order mentalisation, as assessed by the influence play parameter^26,45^. In contrast, damage to several mPFC subregions was associated with distinct components of decision speed^113^, specifically the drift-rate temperature (OFC, aPFC), decision boundary (vACC), and non-decision time (vACC). This suggests a distributed role of the mPFC in regulating the temporal aspects of social strategic interaction^120^. This dissociation sharpens the conceptual boundary between mentalising competence and its temporal execution, highlighting a need for future research to further disentangle these dimensions within the social brain network.

Despite employing a validated interactive social task and a relatively large lesion sample, several limitations should be acknowledged. First, the lesion distribution was not uniform, potentially limiting our ability to detect fine-grained functional specialisation within the mPFC. While our lesion-symptom mapping did not identify distinct subregions associated with higher-order mentalising, future studies with larger and more spatially diverse samples may provide greater resolution. Second, although previous research has implicated the rTPJ as necessary for sophisticated strategic behaviour^11,23,26,38,119^, our study was not designed to directly assess whether the TPJ compensates for mPFC damage in supporting higher-order mentalisation. In our lesion control group, only three participants had lesions involving the TPJ. We conducted analyses with and without these three patients, and results remained substantively the same (see Supplementary Notes for exclusion results). However, future research should expand on this by probing whether compensatory roles are played by other regions, particularly TPJ. Third, our findings are based on naturally occurring lesions, which tend to follow vascular patterns and result in correlated damage across regions. This spatial dependency may obscure the localisation of cognitive functions^43^. In addition, our study did not specifically address the question of lateralisation, and it remains possible that preserved function in the contralateral mPFC may compensate for unilateral damage. In our current sample, approximately half of the mPFC lesion patients had bilateral (*n* = 9) or partial bilateral (*n* = 8) lesions. In control analyses, we again show that results remained substantively the same, limiting them to patients with bilateral lesions (see Supplementary Notes for the analyses including only bilateral mPFC lesion patients). Thus, while lateralisation cannot be fully ruled out as a contributing factor, it is unlikely to account for the overall pattern of results. Larger samples and multivariate lesion-mapping approaches may help address these issues in future research^121–123^. Finally, the present approach reflects an inherent trade-off between experimental control and ecological richness. While the controlled and anonymised interaction enabled isolation of social strategic belief-based reasoning and additional control analyses mitigate concerns about general cognitive impairment, the absence of a formal language assessment means that language-related factors cannot be fully excluded. In addition, the task does not fully capture the richness of naturalistic social interaction, and standard neuropsychological measures do not provide trial-level non-social benchmarks for directly comparing decision dynamics. Even with these limitations, the present work represents an advance beyond traditional ToM paradigms^124–126^ by integrating a more grounded interactive task with computational modelling and lesion-symptom mapping to capture dynamic strategy-based social interactions that require mentalisation.

In conclusion, our findings provide causal evidence that mPFC damage does not impair higher-order mentalising but instead alters the efficiency with which strategic social decisions are made. While belief-updating strategies remain intact, increased decision boundaries suggest a more cautious approach to social strategic interaction following mPFC lesions.

## Methods

### Participants

We recruited participants from a database of 453 neurological patients, university databases, and the Oxford community. Three groups of participants were recruited: one patient group with focal damage to the mPFC (mPFC), another patient group with lesions elsewhere outside of the mPFC as a lesion control group (LC), and one age- and gender-matched healthy control group (HC). For the healthy controls, we excluded individuals who had a history of neurological or psychiatric illness. We also excluded individuals who studied psychology in all groups.

The final sample included 122 participants, comprising 33 patients with mPFC lesions (age range: 39–76 years old, mean = 56.88; 17 females), 16 LC (age range: 28-74 years old, mean = 56.06; 11 females), and 73 HC (age range: 21–80 years old, mean = 60.53; 38 females). No statistical method was used to predetermine sample size; sample size was determined by recruitment feasibility and the availability of participants across groups. The study design was not randomised, and investigators were not blinded to allocation during experiments or outcome assessment.

There were no significant differences in age (*ps* > 0.42) or gender (${\chi }_{(2)}^{2}$ = 1.59, *p* = 0.451) between the three groups (see Supplementary Table S1 for demographics). In addition, the mPFC patients did not differ significantly from the healthy or lesion control groups in the neuropsychological test assessing visual attention and processing speed (Trail Making Test; Part A *ps* > 0.62, Part B *ps* > 0.12). Participants also completed additional neuropsychological assessments indexing general cognitive ability, including the Raven Standard Progressive Matrices (RSPM) and the Addenbrooke’s Cognitive Examination (ACE-III). Group differences were observed on these measures, with lesion patients scoring lower than healthy controls, consistent with broader cognitive consequences of focal brain injury (see Supplementary Notes and Supplementary Table S1). Importantly, the two lesion groups were broadly comparable on these measures.

The lesion control (LC) group was included to control for the general consequences of experiencing brain lesions, beyond comparisons with healthy individuals, while avoiding systematic damage to the mPFC. Lesions in this group were intentionally heterogeneous for both scientific and practical reasons. Although lesions in the LC group were more frequently right-lateralised, inspection of individual lesion maps revealed limited spatial overlap, such that no more than four individuals shared damage in the same region (Fig. 1B). Accordingly, this group was not intended to index effects of any specific non-mPFC region, but rather to provide a control for lesion-related factors that are not specific to mPFC damage.

In this study, most patients had experienced subarachnoid haemorrhages because of aneurysm rupture, particularly anterior communicating artery aneurysms in mPFC patients. Four mPFC lesion patients underwent frontal meningioma resection, and one mPFC lesion patient had suffered an ischaemic stroke. For all patients, the brain damage occurred at least 24 months prior to the testing date (see Supplementary Methods for neurotropic medication).

### Lesion identification

Except for two, all 49 patients underwent structural MRI (1 mm isotropic T1 FSPGR MRI with a 6 mm axial T2 PROPELLER sequence). Two cases were excluded due to the presence of metal surgical clips and an implantable defibrillator, necessitating only a CT scan in those instances. Prior to conducting behavioural testing, a clinical neurologist (SGM) manually traced each participant’s lesion on their brain scan using FSL55 (http://fsl.fmrib.ox.ac.uk/fsl). This traced lesion was subsequently mapped onto the MNI152 template using a Gaussian kernel with a full width at half-maximum of 5 mm. The average volume of the lesions was 2.71 ± 2.65 cm³, varying from 0.02 to 9.74 cm³. Notably, there was no statistically significant difference in lesion volumes between the two lesion groups (mPFC = 2.28 ± 2.38 cm³; LC = 3.60 ± 3.03 cm³; *p* = 0.103; BF_01_ = 1.11). No significant correlation between the overall lesion volume and any VLSM variables was observed in either of the two lesion patient groups (see Supplementary Notes and Supplementary Fig. S14). To visualise the extent of lesions, an overlap map was generated by counting the number of participants with lesions exceeding 10% of the total lesion volume within each voxel (Fig. 1).

### Procedure

Participants joined a single on-site experiment session that began with a clinical assessment by a neurologist (SGM). Participants were then introduced to another ‘participant’ who was in fact a confederate. They were informed that they would be competing against the other participant and subsequently underwent a role assignment procedure to ensure that they believed the other player was present in the lab but remained anonymous in terms of age and gender^60–63^ (see Supplementary Methods and Supplementary Fig. S1). In all cases, participants were consistently assigned the role of the employee.

Participants then read the inspection game task instructions from the screen and filled out a comprehension questionnaire. Participants were only allowed to proceed once all comprehension questions were answered correctly, which was verified by the experimenter. They completed a short practice task to familiarise them with the required response time and were told that during this practice, responses were randomly chosen by a computer. The practice phase was not repeated, as task understanding was confirmed prior to its onset. After the practice phase, the experimenter left the room to check whether the other player was ready, with a brief delay introduced to reinforce the impression of synchronisation with another participant. The start of the main task was preceded by a screen indicating that the system was connecting to the other player, followed by an additional delay before the game commenced. These steps were designed to create a credible social interaction context while maintaining anonymity and experimental control. Notably, no participants suspected the opponent to be artificial or algorithmic in the post-task debriefing. After the series of tasks was completed (three other experimental tasks were reported elsewhere)^54–56^, participants completed a range of questionnaires (see below and Supplementary Methods). Participants received compensation of £10 per hour and a bonus of up to £5 based on their task performance. All participants provided written consent. The study was approved by the Oxford University Medical Sciences Inter Divisional Research Ethics Committee and the National Health Service Ethics.

### Inspection game

The inspection game used in this study was adapted from the task used by Hampton et al. (2008)^45^ and Hill et al. (2017)^26^. In our version of the inspection game, the participant always played the role of the employee and had to choose between work and shirk (Fig. 2A). Each trial began with a fixation cross for 1 s, then the options of work or shirk were presented. Participants pressed the arrow keys on the keyboard to indicate their choice within 4 s, and the chosen option was highlighted with a red square for 2 s. The choice of their opponent, whether to inspect or not inspect, was then presented on the screen for 3 s, alongside the outcome of that trial. The participant received 50 points if they chose work while the employer inspected, and if they shirked while the employer did not inspect. In the other two scenarios, the participant did not get the points and knew that the inspector would get the corresponding points indicated by the outcome payoff matrix (shown in Fig. 2B). The task included 150 trials. Points from the task were added and converted to a bonus payment in GBP at the end of the session. Participants were not informed of the conversion amount but were simply told that the more points they accumulated, the more additional payment they would receive.

Participants were told they were playing against a real opponent in another room (the person whose hand they saw before), but they were actually playing against a computer algorithm based on the influence model fitted to real human data from the previous inspection game^26,45^. This approach enabled precise control over the opponent’s behaviour and allowed us to compare participants’ performance against a consistent ground truth. It also ensured that testing sessions were able to go ahead without another scheduled person present, given the difficulties with testing patient samples and the rare nature of these patients. The artificial opponent’s responses were generated online by the fitted influence model, which would both (1) track player choices and respond optimally to their frequency, (2) use its own past choices to form player expectations, and (3) introduce randomness to the choices based on these two computations. The model parameters were fixed and derived from the average play of the human opponents in a previous study^26^: the first-order belief updating *η* = 0.3, second-order belief updating *κ* = 0.1, and inverse temperature *β* = 1.5. No participant reported a disbelief that the computer algorithm was not, in fact, another person.

We used MATLAB 2012a (Mathworks) and the Cogent 2000 v125 toolbox (www.vislab.ucl.ac.uk/Cogent/) to present the task.

### Assessment and questionnaire measures

Following the main task, participants completed a battery of standardised assessments to characterise individual differences in cognition, affect, and motivation. The Apathy Motivation Index (AMI) was administered to measure trait apathy across behavioural, social, and emotional domains^127^. The Beck Depression Inventory (BDI) assessed depressive symptom severity^128^. The Trail Making Test (TMT) evaluated visual attention and executive function^129^. Fluid intelligence was measured using a shortened version of the Raven Standard Progressive Matrices (RSPM)^130^. The Addenbrooke’s Cognitive Examination (ACE-III) was used to assess global cognitive functioning across attention, memory, fluency, language, and visuospatial domains in participants over 40 and in both lesion groups^131^ (see Supplementary Methods for the full descriptions of the assessments).

After completing all the tasks and tests, we inquired with participants about the specific strategies they employed while playing the inspection game. The strategies identified were as follows: (1) I tried to influence my opponent’s choice, (2) I played an option that worked in the past, (3) I tried to keep track of how my opponent played, (4) I tried to adjust behaviour based on the opponent’s choices, and (5) I felt my opponent was reacting to how I was playing. For each strategy, participants provided a response on a 0–10 scale, with higher scores indicating a greater frequency of using that particular strategy.

### Statistical analysis

All analyses reported were specified a priori based on the task design and theoretical framework, and no additional unreported analyses were conducted. All statistical analyses were conducted in R v4.4.1^132^ using RStudio v2024.09.1 + 394^133^ and Positron (2026.02.1)^134^ (see Supplementary Methods for the used package). Behavioural and model-derived variables were inspected using the Analysis of Variance (ANOVA). Post-hoc pairwise comparisons were conducted using Tukey’s Honest Significant Difference (HSD) test. Bayesian hypothesis testing was used to quantify evidence for both the alternative and null hypotheses. Bayesian factor analyses were performed using the package’s default prior settings (the Rouder et al. (2012)^135^ prior for ANOVA models and a Cauchy prior with scale r = 0.707 for *t* tests). Bayes factors are reported for all statistical tests. The interpretation and reporting of Bayesian factors adhered to the terminology recommended by Schönbrodt and Wagenmakers (2018)^136^. Sensitivity analyses for the primary Bayesian analyses demonstrated that the conclusions were robust across different prior specifications (Supplementary Table S16).

### Computational modelling

#### Model fitting

We applied a drift-diffusion model to the choice behaviour in the task in conjunction with four neurocomputational models involved in social learning behaviour. We selected four learning models based on Hampton et al. (2008)^45^ and adapted from Hill et al. (2017)^26^ (see Supplementary Methods for more model fitting, comparison, and validation details and each model elaboration).

#### Drift-diffusion model (DDM)

The drift-diffusion model (DDM) is a cognitive process model that describes the decision-making process between two alternatives by jointly accounting for choices and reaction times^107^. The model assumes that simple decisions between two choices use a sequential sampling framework, with decisions occurring when the accumulated evidence reaches a threshold. Reaction times are therefore informative about the dynamics of evidence accumulation, allowing evidence accumulation speed and response caution to be dissociated. The value of each choice (e.g., the value of choosing work), which was calculated using separate learning models (see below), was scaled by decision noise parameters, drift rate temperature (d):

1 $${{{{\rm{v}}}}}_{{{{\rm{t}}}}}=d\cdot {V}_{t}^{{Work}}$$

The weighted choice values ${{{{\rm{\nu }}}}}_{{{{\rm{t}}}}}$ were then input into the DDM. The decision boundary parameter ${{{\rm{\alpha }}}}$ in the DDM captured response caution, with higher values indicating that more evidence was required before committing to a choice. The non-decision time parameter ${{{\rm{\tau }}}}$ was also added to each trial to capture components of response time unrelated to evidence accumulation, such as sensory encoding and motor execution.

#### Reinforcement learning model (RL-DDM)

The first model employed is a conventional reinforcement learning model, specifically the Q-learning algorithm. The model suggests that participants do not pay attention to the opponent but acquire knowledge from their previous actions and rewards, subsequently making informed decisions. Reinforcement learning is a prediction-error-based model that modifies the value of each choice for each trial *t* in response to a prediction error δ weighted by a learning rate η:

2 $${V}_{t}^{{Work}}={V}_{t-1}^{{Work}}+{{{\rm{\eta }}}}\cdot {\delta }_{t-1}$$

The updated choice value was then used as the input of the DDM in Eq. (1).

#### Fictitious play model (Fictitious-DDM)

The fictitious play model, the second model employed for fitting, posits that the participant does not track the value of their own actions but instead monitors their opponent’s probability of selecting an action (specifically, the probability of choosing not inspect) based on the opponent’s previous play history:

3 $${P}_{t}^{{Not}\,{Inspect}}={P}_{t-1}^{{NotInspect}}+{{{\rm{\eta }}}}\cdot {{{{\rm{\delta }}}}}_{t-1}^{P}.$$

This first-order belief is used to select the action that maximises the expected outcome. The participant’s choice value in the fictitious play model is determined based on the payoff matrix (Fig. 2B):

4 $${V}_{t}^{{Work}}=50-100\cdot {P}_{t}^{{Not}\,{Inspect}}$$

The anticipated value ${V}_{t}^{{Work}}$ is subsequently input into the DDM function as per Eq. (1).

#### Influence play model (Influence-DDM)

The influence play model integrates both first- and second-order beliefs, suggesting that participants’ decisions in trial t are influenced by both observations of the opponent’s past choices and inferences about what beliefs the opponent may form based on observing the participant’s past choices:

5 $${P}_{t}^{{Not}\,{Inspect}}={P}_{t-1}^{{Not}\,{Inspect}}+{{{\rm{\eta }}}}\cdot {{{{\rm{\delta }}}}}_{t-1}^{P}+{{{\rm{\kappa }}}}\cdot {{{{\rm{\delta }}}}}_{t-1}^{Q}$$

The second-order belief in the influence learning model is weighted by ${{{\rm{\kappa }}}}$.${P}_{t}^{{Not}\,{Inspect}}$ is obtained by combining the first-order (fictitious play) belief update ${{{\rm{\eta }}}}\cdot {{{{\rm{\delta }}}}}_{t-1}^{P}$ and the second-order (influence play) belief update ${{{\rm{\kappa }}}}\cdot {{{{\rm{\delta }}}}}_{t-1}^{Q}$. ${P}_{t}^{{Not}\,{Inspect}}$ is then used to update the choice value ${V}_{t}^{{Work}}$ in Eq. (4) and incorporated into the DDM. If the weight ${{{\rm{\kappa }}}}$ for second-order belief is equal to 0, the model reduces to the fictitious play model.

#### Simple influence model (Simple influence-DDM)

The last model represents the simplified version of the influence learning model:

6 $${V}_{t}^{{Work}}={{{\rm{\eta }}}}\cdot {P}_{t-1}+{{{\rm{\kappa }}}}\cdot {Q}_{t-1}$$

The value of the participant’s choice is updated based on both first- and second-order beliefs. The opponent’s choice, ${P}_{t-1}$, is weighted by ${{{\rm{\eta }}}}$, and while the participant’s own choice, ${Q}_{t-1}$, is weighted by ${{{\rm{\kappa }}}}$. The ${V}_{t}^{{Work}}$ of this simple influence model was then incorporated into the DDM, where the weighted choice value ${{{{\rm{\nu }}}}}_{{{{\rm{t}}}}}$ was derived as

7 $${{{{\rm{\nu }}}}}_{{{{\rm{t}}}}}=d+{V}_{t}^{{Work}}$$

with a constant parameter d added.

#### Model comparison

For comparison, each model’s fitting results were calculated at the subject level using the Bayesian Information Criterion (BIC)^137^. To mitigate the risk of overfitting and enhance the model’s generalisability, BIC incorporated a penalty for free parameters. The model with the lowest BIC is regarded as the most suitable fit for the data.

#### Parameter recovery

To validate the winning model, we conducted parameter recovery simulations. Data were simulated using the final parameter values from the optimal fit of the influence-DDM model. A confusion matrix was constructed to assess the correlations between simulated and recovered parameter values^137^. The strong parameter recovery for the winning model is evident from the high correlations between simulated and recovered parameters on the diagonal and the low correlations off the diagonal (as shown in Fig. 3B) (see Supplementary Notes and Supplementary Fig. S5 for model validation).

### Voxel-based lesion-symptom mapping (VLSM)

We conducted Voxel-based lesion-symptom mapping (VLSM) analyses on the best-fitting parameters of the influence-DDM model: fictitious play ${{{\rm{\eta }}}}$ and influence play ${{{\rm{\kappa }}}}$ to examine links between specific brain damage and mentalising ability. We also analysed drift rate temperature d, boundary ${{{\rm{\alpha }}}}$, and non-decision time ${{{\rm{\tau }}}}$, to assess associations with task performance and response latency. The VLSM analyses assess voxel-level associations between lesion location and model-derived parameters and do not rely on the presence of significant group differences in these parameters. This approach, therefore, allows detection of spatially specific lesion–parameter relationships that may not be evident in group-level comparisons, particularly when effects are confined to smaller subregions or relate to variability within groups.

For each participant, parameters were ranked to rectify the skewness of the residual distribution and subsequently z-scored^65^. Separate tests were conducted for positive and negative ranks to evaluate these hypotheses. Rankings were input into the FMRIB software library (FSL v6.0.7.15)^138^, and permutation-based VLSM analyses were performed using the randomise function to compare each damaged voxel across all patients (mPFC lesion and lesion controls)^65^. For the primary analysis, we performed a whole-brain VLSM including all lesion patients (mPFC lesion group and lesion control group). This approach provides a conservative test of lesion–behaviour associations by leveraging heterogeneous lesion coverage across the brain while controlling for nonspecific lesion effects. Voxels were only included if at least five patients had that voxel damaged to some extent^139^. Lesion maps were mirrored to increase power, given no prior lateralisation hypothesis.

We used threshold-free cluster enhancement (TFCE) in FSL with 5000 permutations and default TFCE settings, which enhances power and employs non-arbitrary cluster size definitions^64^ (see Supplementary Methods). Bonferroni correction was applied for multiple comparisons^140^: *p* < 0.01. Significant regions were visualised with binarised masks applied to *t*-values and labelled using BioImage Suite (https://www.bioimagesuite.org)^141^ and the fourth edition of Atlas of the Human Brain (https://www.thehumanbrain.info/)^142^.

In addition to the whole-brain analysis, we conducted a supplementary VLSM restricted to the focal mPFC lesion group to examine subregional variability within the mPFC. This mPFC-only analysis is reported in the Supplementary Materials and is interpreted as a complementary within-region analysis.

### Reporting summary

Further information on research design is available in the Nature Portfolio Reporting Summary linked to this article.

## Supplementary information

Supplementary Information

Reporting Summary

Transparent Peer Review file

## Data Availability

The behavioural, questionnaire, computational modelling, and lesion-symptom mapping data generated in this study have been deposited in the Open Science Framework (OSF) repository under accession code https://doi.org/10.17605/OSF.IO/AX85S. The processed behavioural and neuropsychological data used for all analyses are publicly available in the OSF repository. Lesion-symptom mapping input files and permutation-based statistical outputs are also provided to enable full reproducibility of the reported analyses. Raw neuroimaging and clinical data are not publicly available due to participant privacy and ethical restrictions under institutional data protection policies. All data required to reproduce the results reported in this study are available in the OSF repository under the same accession code.

## Code availability

All analysis code used in this study has been deposited in the Open Science Framework (OSF) repository under accession code https://doi.org/10.17605/OSF.IO/AX85S.
