# Supplementary material for: Preserved higher-order mentalising during strategic interactions after medial prefrontal cortex damage
Source: Nat Commun. 2026 Oct 10;17:11514. doi: 10.1038/s41467-026-77235-3 (PMC13745589; doi:10.1038/s41467-026-77235-3)
Supplement: Supplementary file 1 [file 41467_2026_77235_MOESM1_ESM.pdf]

Supplementary Information for

Preserved Higher-order Mentalising During Strategic Interactions After Medial Prefrontal Cortex Damage

Supplementary Methods

Participants' neurotropic medications

Thirteen patients with lesions in the medial prefrontal cortex (mPFC) were prescribed antihypertensive medications. Two patients were concurrently receiving antidepressants (amitriptyline), one patient was taking an anticonvulsant (levetiracetam), and one patient was prescribed pregabalin for migraine management.

In the lesion control group (LC), four patients were prescribed antihypertensive medications. Two patients were concurrently taking antidepressants (citalopram). One of these two patients was also taking paroxetine, while the other was prescribed pregabalin. Additionally, one lesion control group patient was concurrently taking anticonvulsants (lamotrigine and levetiracetam).

Role assignment process

To establish an anonymous but real social context prior to the task, participants underwent a standardised role assignment procedure[1–4] (Figure S1). Upon arrival, each participant was informed that they would be randomly assigned to the role of either employer or employee in the upcoming inspection game. In reality, all participants were consistently assigned the role of employee. To reinforce the belief that another real participant was present while minimising identity-based biases, the participant and a confederate researcher (posing as another participant) were introduced under controlled conditions without direct visual contact. Specifically, the two were positioned on opposite sides of a door, allowing them to see two experimenters simultaneously while preventing face-to-face interaction. Both the participant and the confederate wore rubber gloves and were instructed to silently wave at each other during the introduction.

This brief controlled interaction served to create a believable yet depersonalised social context for the subsequent game. Participants were reminded that the inspection game would be played against the other participant they had met at the beginning of the session. Connection delays prior to the main task further reinforced the impression of real-time interaction. Post-task debriefing did not indicate that participants suspected the opponent to be artificial or algorithmic, suggesting that the interaction was experienced as social from the participants' perspective. The use of a non-video-based interaction was a deliberate design choice to minimise prior impressions or biases related to facial appearance or identity, thereby allowing strategic belief-based reasoning to be examined in a controlled social context.

Assessments

Following the main task, participants completed standardised assessments to assess cognitive, affective, and motivational functioning (Table S1). These measures were included to ensure comparability between the two lesion groups and to verify that the lesion control group served as an appropriate control. In addition, certain assessments (e.g., Trail Making Test, see below) were used to confirm that participants, including lesion patients, had typical cognitive function. Detailed descriptions of each assessment are provided below.

Apathy Motivation Index. The Apathy Motivation Index (AMI) is a scale designed to assess apathetic traits[5]. The scale consists of 18 items on a 5-point Likert scale (0-4) and measures three individual differences in apathy-motivation: behavioural activation, social motivation, and emotional sensitivity. We reverse-scored participants' ratings, resulting in a higher score reflecting an increased level of apathy.

Beck Depression Inventory. The Beck Depression Inventory (BDI) was used to assess participants' symptoms of depression[6]. Participants rate on a 4-point Likert scale (0-3) 21 items. Higher cumulative scores indicate increased severity of depressive symptoms.

Trail Making Test. The Trail Making Test (TMT) is a subset of the Army Individual Test Battery (1944) designed to assess general mental ability, particularly general intelligence. The test can be utilised to evaluate cognitive abilities in patients with brain damage[7] and

is commonly employed as a clinical assessment tool. The test comprises two parts: Part A, in which participants must connect 25 scattered numbers sequentially in ascending order (i.e., 1-2-3-4, etc.); Part B, in which participants are instructed to connect the items alternating between 13 numbers (1-13) and 12 letters (A-L) (i.e., 1-A-2-B, etc.). Participants were required to complete the tasks as swiftly as possible and rectify any errors made. Part A is designed to assess visual attention, while Part B is intended to evaluate executive function. The time taken to complete each task was recorded as the score for the subsequent analysis.

Raven Standard Progressive Matrices. The Raven Standard Progressive Matrices (RSPM), commonly referred to as Raven's test, is the initial instrument within the Raven's Progressive Matrices series, which serves as a non-verbal assessment of fluid intelligence[8]. In our study, we used one of the five sets, comprising 12 testing matrices. The 3x3 matrix presented 8 figures, with the 9th figure missing. Participants were tasked with identifying the sole correct figure among the 8 alternative figures that align with the established patterns and shape progression of the existing 8 figures. The task has a time limit of 10 minutes. Each correct answer was recorded as 1 point, and these points were accumulated for subsequent analysis.

Addenbrooke's Cognitive Examination. Participants in the healthy control group (HC) who were age > 40, mPFC lesion patients, and lesion control patients were assessed for cognitive deficits using the Addenbrooke's Cognitive Examination (ACE-III)[9]. This neuropsychological test comprises five cognitive domains: attention, memory, fluency, language, and visuospatial skills. The total score of the five domains was added up, with the lower score signifying the deficit in cognitive function.

Statistical analysis packages

Statistical analyses were performed using R v4.4.1[10] along with RStudio v2024.09.1+394[11]. All statistical figures were generated using the {*ggplot2*} package (version 3.5.1)[12]. Behavioural data and model-fitting parameters were inspected using the {*stats*} (version 4.4.2), {dplyr} (version 1.1.4)[13], and {tidyverse} (version 2.0.0)[14] packages. Post-hoc analyses when significant were conducted using Tukey's Honest Significant Difference method ('*TukeyHSD*' from {*stats*}). For the insignificant results, Bayesian factors (BF_{01})

were calculated using the 'anovaBF' function from the {BayesFactor} package (version 0.9.12.4.7)[15]. Alternatively, Bayesian factors were calculated for paired and independent *t*-test designs using the 'ttestBF' function from the same package.

Additional analyses examining the effects of response time on second-order belief updates were conducted using robust linear mixed-effects models (RLMMS; '*rlmer*' function from the {*robustlmm*} package version 3.3.1)[16]. The model included update, group, and last condition as fixed effects, along with their interactions, and a random intercept for participants. Post-hoc comparisons were performed using the {*emmeans*} package (version 1.10.5)[17], including pairwise contrasts within groups and interaction contrasts across update × group conditions.

Computational modelling details

Model fitting. To examine the computational mechanisms underlying participants' choice behaviour, we fitted a series of drift diffusion models (DDMs) incorporating four learning models. We fitted the models using R and RStudio, using the '*dwiener*' function from the {*RWiener*} package (version 1.3.3) along with {doParallel} (version 1.0.17)[18] and {foreach} (version 1.5.2)[19] packages to fit the Wiener diffusion model[20].

Drift diffusion model (DDM). We applied the DDM to jointly account for participants' choices and response times (RTs). In our model fitting, bias parameters were set to 0.5, representing the absence of initial bias. The model calculated the likelihood of the response time (RT) and choice on each trial and used the total maximum likelihood to acquire the best parameter fits for each participant.

Reinforcement learning model (RL-DDM). The Q-learning model we used updates choice values based on the prediction error signal. Here, we elaborate on how this prediction error was computed and used to guide value updating on each trial. The prediction error quantifies the discrepancy between the predicted outcome and the actual outcome, thereby reflecting the selection of action and whether it was subsequently rewarded:

$$\delta_{t-1} = R_{t-1} - V_{t-1}^{Work}. \quad \text{(S1)}$$

Fictitious play model (Fictitious-DDM). The fictitious play model updates the participant's belief about the opponent's behaviour based on a prediction error signal. This prediction error, δ_{t-1}^{P}, was computed from the opponent's actual choice on the previous trial and the model's prior estimate of the probability of 'not Inspect'. The η-weighted prediction error δ_{t-1}^{P} is updated as follows:

$$\delta_{t-1}^{P} = P_{t-1} - P_{t-1}^{Not\ Inspect}. \tag{S2}$$

P_{t-1} represents the observed action taken by the opponent, which is assigned a value of 1 if the employer chose 'not Inspect' and 0 if the opponent chose 'inspect'.

Influence play model (Influence-DDM). The influence play incorporates second-order belief updates, allowing participants to infer how their own actions might shape the opponent's beliefs. Here, we expand on the calculation of the second-order prediction error, δ_{t-1}^{Q}, which quantifies the discrepancy between the participant's actual choice and the opponent's inferred expectation of that choice. The prediction error of the inferred opponent's belief about the participant's action is calculated as:

$$\delta_{t-1}^{Q} = Q_{t-1} - Q_{t-1}^{Work}, \tag{S3}$$

where Q_{t-1} is the participant's action at the trial, recorded as 1 when the participant chooses 'work' and 0 when the participant chooses 'shirk'. Q_{t-1}^{Work} is the opponent's inferred participant's working probability:

$$Q_{t-1}^{Work} = \frac{1}{5} - \frac{1}{5} \cdot \frac{1}{25 \cdot d} \cdot \log\left(\frac{1 - P_{t-1}^{Not\ Inspect}}{P_{t-1}^{Not\ Inspect}}\right). \tag{S4}$$

Model comparison. We employed a random grid with 50,000 parameter sets to identify the most promising parameter sets. This grid yielded 10 sets of parameters that exhibited the highest likelihood. These 10 sets were subsequently used as initial starting points for gradient optimisation. The final parameter values were determined from the optimal fit obtained among these 10 sets. To evaluate model performance and prevent overfitting, we computed the Bayesian Information Criterion (BIC) for each participant and model [21]. BIC balances model fit against complexity by incorporating a penalty term proportional to the number of free parameters:

$$BIC = k_m \log(T) - 2 \log LL\,. \tag{S5}$$

The $k_m \log(T)$ serves as the penalty for the number of parameters in the model, where k_m is the number of parameters in the model and T is the number of each participant's completed trials. Models with lower BIC values indicate better trade-offs between explanatory power and parsimony. While the main text reports BIC comparisons based on the full mixed group sample, here we present BIC comparisons separately for each participant group (see ***Supplement Results*** and **Figure S2** below).

Parameter recovery. To validate the winning model, we performed a parameter recovery analysis using simulated datasets. These datasets were generated with the final parameter estimates from the influence-DDM model (bias fixed at 0.5), using the '*rwiener*' function from the {*RWiener*} package (version 1.3.3)[20]. The same model was then refitted to the simulated data to obtain recovered parameters. To evaluate recovery accuracy, we computed a confusion matrix showing the correlations between simulated and recovered parameters[21]. Successful recovery was indicated by high correlations along the diagonal and low correlations elsewhere.

Posterior predictive check. We further examined model validity by conducting posterior predictive checks to assess how well the model captured both choices (probability of selecting work) and RTs. For each participant and trial, we simulated 100 responses using the final fitted parameters from the influence-DDM model (again with bias fixed at 0.5). From these simulations, we extracted predicted probabilities of choosing work and associated RTs, which were then compared to observed data (see Supplement Results and Figure S3 below). Simulations were implemented using the same '*rwiener*' function from the {*RWiener*} package (version 1.3.3).

Voxel-based lesion-symptom mapping (VLSM)

Voxel-based lesion-symptom mapping (VLSM) analyses were conducted using permutation-based threshold-free cluster enhancement (TFCE) in FMRIB Software Library (FSL)[22]. Here, we provide additional implementation details. We included both lesion groups in the analysis, where lesion voxels were only included if at least five patients had that voxel damaged to some extent[23]. TFCE was applied via the *randomise* function with 5,000 permutations and default settings optimised for voxel-based data[24].

Permutation testing involves repeatedly analysing randomly shuffled data to compute voxel-wise *p*-values, estimating the likelihood that observed effects could arise by chance. This approach requires fewer assumptions, accurately reflects the structure of the data, and enhances sensitivity by amplifying the signal from spatially contiguous clusters, without relying on arbitrary thresholding. Bonferroni correction was applied for multiple comparisons[25] ,and the results were labelled using the fourth edition of Atlas of the Human Brain (https://www.thehumanbrain.info/)[26].

Supplementary Notes

Robustness analyses of response time (RT) effects

To assess the robustness of the observed group differences in response times (RTs), we conducted a series of additional analyses addressing the potential influence of extreme trials, extreme individuals, the analytic approach, and distributional assumptions.

Extreme RTs trimming. In the main analyses, RTs were analysed without excluding extreme values. To examine whether the results were driven by unusually short or long RTs, we performed a trimming procedure in which trials below the 5th percentile and above the 95th percentile of each participant's RT distribution were excluded (Ratcliff, 1993). The pattern of group differences in RTs remained unchanged following trimming, indicating that the effects were not driven by extreme trials (see Table S2).

Log-transformation of RTs. We analysed untransformed RTs in the main analyses to maintain interpretability and consistency with the drift–diffusion modelling framework, which explicitly decomposes RT into decision-related and non-decision components. As a robustness check, we repeated the main analyses using log-transformed RTs. These analyses yielded the same pattern of results as the untransformed analyses, confirming that the findings did not depend on the scale of RT measurement (see Table S3).

Robust linear mixed-effects modelling. To further account for within-participant variability and to reduce sensitivity to extreme observations, we re-analysed the RT data using robust mixed-effects models with random intercepts for participants that downweigh the influence of extreme trial-level observations. The approach yielded results consistent with the main analyses, demonstrating that the observed group differences in RTs are robust to alternative modelling strategies (see Table S4).

Distributional analyses at the participant and trial levels. We additionally examined RT distributions at both the participant and trial levels. Inspection of participant-level median RTs revealed that a small number of participants exceeded a conservative 2.5 SD criterion within their respective groups (N: HC = 2, mPFC = 1, LC = 2); however, these individuals were distributed across all groups rather than being specific to the mPFC group (see Figure S3 for participants' median RT distribution). Excluding these participants from

the analyses did not alter the pattern of results (see Table S5). At the trial level, we conducted distributional analyses using permutation-based non-parametric tests to assess whether group differences could be attributed to a small subset of extreme RTs[27,28]. Pairwise comparisons using Kolmogorov–Smirnov tests confirmed distributional differences between the mPFC group and both control groups, whereas differences between HC and LC were comparatively small (mPFC vs HC: D = 0.139, $p < 0.001$; mPFC vs LC: D = 0.149, $p < 0.001$; HC vs LC: D =0.034, $p = 0.024$). Permutation-based energy tests yielded a similar pattern (see Table S6). These analyses indicated that RT differences reflect distribution-level shifts rather than being driven by a limited number of extreme trials.

Across all robustness analyses—including trimming, transformation, mixed-effects modelling, and distributional assessments—the pattern of RT results remained consistent. Together, these findings demonstrate that the observed RT effects are robust and not driven by extreme trials, extreme individuals, or specific analytic choices.

Task adaptation and practice-phase response times

Trial-wise changes in response time. To examine whether response times (RTs) changed over the course of the experiment, and whether groups differed in their rate of adaptation, we fitted robust linear mixed-effects models including trial progression and its interaction with group. Across participants, RTs decreased reliably over trials, indicating general task adaptation. Crucially, there was no evidence for a group-by-trial interaction, suggesting that participants with mPFC lesions did not adapt more slowly to the task than healthy or lesion control groups (Table S7).

Response times during the practice phase. We additionally examined RTs during the practice phase to assess whether group differences in RT might reflect differences in early task engagement or familiarisation. RTs were longer during the practice phase than during the main task across all groups, consistent with participants familiarising themselves with the task structure. The mPFC lesion group showed a larger reduction in RT from the practice to the main task compared to healthy controls, while not differing significantly from lesion controls (Table S8).

Together, these analyses indicate that the observed group differences in RT are unlikely to be explained by slower task adaptation or delayed familiarisation in the mPFC group. These findings should, however, be interpreted with caution, given the relatively small number (<10) of practice trials available for RT analysis.

Influence-DDM best fits the mPFC and HC groups, while the LC group favours simpler fictitious-DDM

As shown in Figure 3C & S4, both the HC group and the mPFC lesion group showed the lowest BIC for the influence-DDM model, suggesting that incorporating second-order belief updating best captured their strategic choices. In contrast, the LC group showed the best fit for the fictitious-DDM model (mean BIC = 282.89), with the influence-DDM yielding a higher BIC value (mean BIC = 284.39). However, this BIC difference was relatively small (ΔBIC < 1.5).

To formally assess whether the fictitious-DDM provided a meaningful advantage in the LC group, we conducted additional analyses comparing models based on how well they predicted participants' choices. Predictive accuracy was evaluated by simulating trial-by-trial choices for each participant using their model-derived parameter estimates and comparing these simulated choices with observed choices across trials. Contrary to what might be inferred from the small BIC difference, the influence-DDM showed slightly higher predictive accuracy than the fictitious-DDM even within the LC group (mean difference = 2.74 percentage points ± 0.77 SEM; paired $t(15) = 3.54$, $p = 0.003$; Figure S6A-S6C; see Figure F6B for the direct comparison between the fictitious-DDM and the influence-DDM).

These results indicate that the apparent preference for the fictitious-DDM in the LC group based on BIC alone does not reflect a robust or meaningful difference in the underlying decision strategy. Rather, they suggest that both models capture LC behaviour similarly well, and that the inclusion of influence-based mentalising does not impair model performance in this group. The small sample size of the LC group (N = 16) may also contribute to greater uncertainty in model comparison metrics. Additionally, the heterogeneity of lesion locations in the LC group may have led to more variable strategies that are better captured by simpler belief-tracking mechanisms, rather than the more complex higher-order reasoning implemented in the influence model. Future studies with

larger LC samples and more homogeneous lesion profiles may help clarify whether this pattern reflects a true cognitive distinction or sample-specific variability. Taken together, these findings support the use of the Influence-DDM as a common computational framework across groups.

Model validation confirms that influence-DDM captures both choice probability and RT patterns

Model fitting identified the influence-DDM model as the best-fitting model of participants' behaviour, with strong parameter recovery supporting the robustness of the estimated values. To further validate the model, we conducted posterior predictive checks to assess how well the fitted model could reproduce observed behavioural patterns. Participants' choices (probability of choosing work) were accurately simulated, with the model correctly predicting a heightened likelihood of selecting 'work' when there are greater disparities in choice values (work – shirk) (Figure S5A) and yielding predicted P(Work) rates that strongly correlated with the actual data (Figure S5C, $r(120) = 0.90$, $p < 0.001$). The model also reproduced RT patterns, showing that choices with more pronounced relative value differences were faster (Figure S5B) and showed a robust correlation between simulated and observed RTs (Figure S5D, $r(120) = 0.98$, $p < 0.001$). Additional posterior predictive plots separated by group are shown in Figure S5E-S5F.

Neuropsychological measures and cognitive control analyses

Group comparisons on general cognitive measures showed that the mPFC lesion group scored lower than healthy controls on the Raven Standard Progressive Matrices (RSPM), while not differing significantly from the lesion control group. A similar pattern was observed for global cognitive functioning as assessed by the Addenbrooke's Cognitive Examination (ACE-III), which was administered to lesion patients and an age-matched subset of healthy controls (>40 years). In contrast, performance on the Trail Making Test (Parts A and B), which indexes visual attention and non-social processing speed, did not differ significantly between the three groups. This pattern is consistent with expectations in neurological samples and indicates that, while patients differed from healthy controls on some general cognitive measures, the two lesion groups were broadly matched, particularly on measures most relevant to response speed (see Table S1).

To assess whether the observed group differences in response time (RT) could be explained by general cognitive ability or non-social processing speed, we conducted an additional control analysis including both RSPM scores and Trail Making Test performance as covariates in the RT models. The main effect of group on RT remained significant, and the pattern and magnitude of group differences were unchanged (Table S9). Inclusion of these covariates did not alter any other key findings: there were still no group differences in overall payoff (Table S10), and group comparisons of model-derived parameters yielded the same pattern of results as in the primary analyses (Table S11). Together, these results indicate that the slower decision-making observed in mPFC patients cannot be explained by differences in general cognitive ability or non-social processing speed alone, and that the main behavioural and computational conclusions remain robust after accounting for these factors.

Correlations and collinearity between model parameters

To assess the degree of dependence between model parameters, we examined pairwise correlations among all five parameters of the Influence-DDM, including the two mentalising parameters (influence play κ and fictitious play η) and the three decision-related DDM parameters (decision boundary α, drift rate temperature d, and non-decision time τ). Spearman correlation coefficients were computed across participants and are shown in Figure S8.

Overall, correlations between parameters were low to modest, indicating that the parameters captured largely distinct aspects of behaviour. To further assess potential multicollinearity among the decision-related parameters, we conducted variance inflation factor (VIF) analyses focusing on the three DDM parameters. Specifically, each DDM parameter was regressed on the remaining two parameters, and VIFs were computed from these models. All VIF values were close to 1 (all VIFs $\approx$ 1.01), well below conventional thresholds for concern, indicating negligible multicollinearity.

Together, these analyses confirm that the five model parameters are largely independent and that subsequent analyses treating these parameters separately are unlikely to be confounded by shared variance.

Drift rate and non-decision time parameters are comparable across groups

Group comparisons of parameters from the best-fitting influence-DDM model revealed a significant difference in the decision boundary α. Here, we report the results for the remaining two DDM parameters: drift rate temperature d and non-decision time τ. These parameters did not differ significantly between the mPFC lesion group, lesion controls (LC), and healthy controls (HC). For drift rate temperature d (Figure S9A), the overall group effect was not significant ($F(2,119) = 2.67$, $p = 0.073$, $\eta^2 = 0.043$, $BF_{01} = 1.13$), and none of the pairwise comparisons reached significance (mPFC vs HC: diff [95% CI] = -0.01 [-0.02 0.01], $p = 0.146$, $BF_{01} = 1.13$; LC vs HC: diff [95% CI] = -0.01 [-0.02 0.01], $p = 0.194$, BF01 = 1.36; LC vs mPFC: diff [95% CI] = -0.01 [-0.02 0.01], $p = 0.959$, BF01 = 2.90). Similarly, for non-decision time τ (Figure S9B), there was no evidence of group differences ($F(2,119) = 0.72$, $p = 0.488$, $\eta^2 = 0.012$, $BF_{01} = 6.16$), and all pairwise contrasts showed no significant differences (mPFC vs HC: diff [95% CI] = 0.01 [-0.04 0.06], $p = 0.873$, $BF_{01} = 4.08$; LC vs HC: diff [95% CI] = -0.03 [-0.10 0.04], $p = 0.613$, BF01 = 2.45; LC vs mPFC: diff [95% CI] = -0.04 [-0.11 0.04], $p = 0.457$, BF01 = 1.97). These results suggest that all three groups processed perceptual information with comparable efficiency and were similarly sensitive to value-based evidence during choice.

Decision boundary, payoff, and simulation analyses

In the comparisons of model-derived parameters, the mPFC lesion patients exhibited higher decision boundaries, indicating a more cautious evidence accumulation process before making the final decision. In principle, such increased caution could lead to better task performance by reducing costly errors and thereby increasing payoff. However, in the observed data, the decision boundary was not significantly associated with payoff across any of the groups (Figure S10).

To assess whether the decision boundary can, in principle, influence task performance, we conducted simulation analyses in which the decision boundary parameter (α) was systematically varied across three levels (low, medium, and high). These boundary levels were defined relative to each participant's fitted decision boundary, using a fixed offset to generate lower and higher boundary values within a plausible parameter range. All other model parameters were held constant at each individual's fitted values. For each boundary

level, choices were simulated across trials, and expected payoff was calculated from the simulated behaviour.

These simulations revealed that higher decision boundaries led to modest increases in payoff, consistent with a more cautious decision strategy that reduces decision errors (Table S12). Quantitatively, increasing the boundary from the lowest to the highest level produced an overall improvement of approximately 2.1% in accumulated expected payoff. This pattern demonstrates that the decision boundary can influence payoff when manipulated in isolation under controlled conditions. By contrast, in the empirical data, boundary differences did not translate into systematic differences in payoff, likely because task performance reflects the joint influence of multiple interacting model parameters rather than the decision boundary alone. Together, the simulation and empirical analyses clarify that the decision boundary primarily captures differences in decision dynamics, rather than differences in overall task performance.

Response time patterns reflect second-order belief updating and strategic deliberation in mPFC patients

Having established that greater engagement in influence play was associated with improved task performance (Figure 4B), we next tested whether second-order belief updates were related to participants' response times (RTs). Specifically, we examine whether the group differences we observed in RTs reflected mentalising-related processes captured by the computational model. In this analysis, we used second-order belief updates derived from the winning influence-DDM model (corresponding to the second-order prediction error, δ^{Q}_{t-1}, as defined in Formula S3), and investigated whether trials with larger belief updates were associated with faster or slower responses.

Importantly, this analysis linked the belief update and RT from the same trial, meaning it assessed whether participants responded faster or slower on trials where they ultimately experienced larger belief updating. Updates were categorised into high and low groups based on their absolute magnitude using a median split. A robust linear mixed-effects model, predicting RT from update magnitude (Low vs. High), group (mPFC, HC, LC), previous trial results (Success vs. Failure), and their interactions.

The model revealed a significant interaction between update and group (Figure S11A, diff [95% CI] = 0.05 [0.01 0.09], $p = 0.005$), indicating that the HC group showed a significantly smaller RT difference between high and low update trials compared to the mPFC group. Post-hoc comparisons further showed that both mPFC and LC groups responded significantly faster on high-update trials compared to low-update trials (mPFC: z-ratio = 3.338, $p = 0.001$, $BF_{10} = 25$; LC: z-ratio = 2.539, $p = 0.011$, $BF_{10} = 2.27$), whereas the HC group showed no such effect (HC: z-ratio = 0.800, $p = 0.424$, $BF_{01} = 8.02$). To further examine the group differences, we conducted pairwise contrasts on RT differences (low minus high update) between groups (Figure S11B). This analysis further revealed a significant difference between the mPFC and HC groups (z-ratio = 2.306, $p = 0.021$, $BF_{10} = 1.30$), and no difference between the other two comparisons (HC vs LC: z-ratio = -1.938, $p = 0.034$, $BF_{01} = 1.27$; mPFC vs. LC: z-ratio = -0.145, $p = 0.885$, $BF_{01} = 3.28$). Repeating the analysis with continuous belief updates yielded the same qualitative pattern of results as the median-split approach, confirming that the reported effects are not an artefact of binning (Table S15).

Participants with mPFC lesions tended to exhibit larger second-order belief updates on faster-response trials, and their response times varied more strongly between high and low update trials. One possible interpretation is that, within the mPFC group, trials with longer response times were associated with smaller belief updates, suggesting that those choices may have more closely aligned with their second-order predictions (i.e., expectations about how the opponent might interpret their actions). In other words, when mPFC patients spent more time deliberating, they may have engaged in more accurate higher-order mentalising, resulting in decisions that required less belief updating post hoc. While this explanation remains speculative, the pattern raises the possibility that slower decisions in the mPFC group may reflect more deliberate and strategic use of second-order beliefs, potentially compensating for efficiency in social strategic inference.

Lesion volume does not predict model parameters used in VLSM analyses

Before conducting VLSM, we examined whether the severity of brain damage, as indexed by total lesion volume, was associated with the computational model parameters used in

the VLSM analyses. This step was critical to ensure that any observed brain-behaviour associations could not be explained simply by lesion size.

Specifically, we computed the Spearman correlation between lesion volume and each of the five key model parameters (drift rate temperature d, decision boundary α, non-decision time τ, fictitious play η, and influence play κ) across all lesion patients. Results showed none of the model parameters exhibited a significant correlation with lesion volume (Figure S14). This indicates that the model-derived measures of behaviour were not systematically related to overall lesion severity. Accordingly, we proceeded with VLSM analyses under the assumption that regional lesion effects on model parameters could be meaningfully localised, rather than being confounded by general damage extent.

Control analyses excluding patients with temporo-parietal junction damage

Our lesion control group contained 3 patients with extensive damage to the right TPJ. We therefore conducted an additional analysis excluding these 3 lesion control (LC) participants. This exclusion yielded a reduced LC subgroup (LC-E, n = 13).

As summarised in Table S1, comparisons across the three groups (HC, mPFC, LC-E) showed no significant differences in age and gender. The task performance, calculated by the payoff accumulation, also remained similar between groups, with Bayes factors providing substantial evidence in favour of the null hypothesis ($F_{(2,116)}$ = 0.29, p = 0.750, BF_{01} = 7.39). Similar to our main results, the mPFC group continued to show significantly slower response times than the HC group (diff [95% CI] = -0.23 [-0.40 -0.06], p = 0.005). However, the previously observed difference between mPFC and LC groups was attenuated; the mPFC group did not significantly differ from the LC-E group (diff [95% CI] = -0.23 [-0.50 0.03], p = 0.097), with a Bayes factor suggesting anecdotal evidence for no difference (BF_{01} = 1.13), which is not surprising given the reduced power of the additional analyses with a small LC group (Table S13). All modelling results from the DDM model remained the same (Figure S12). The influence play parameter, indexing higher-order mentalising, was again positively associated with task payoff.

Excluding unilateral lesions confirms preserved mentalising and slower response following bilateral mPFC damage

We also conducted a second control analysis that tested whether lesion location (bilateral vs. unilateral) accounted for our results. Particularly, the possibility that the preserved mentalising ability observed in mPFC lesion patients might be supported by compensatory mechanisms in the contralateral (intact) mPFC. We re-ran our analyses, including only those patients with bilateral or partially bilateral mPFC lesions (mPFC-B, N=17), excluding all unilateral cases.

We observed the same pattern as our original findings (Table S14). There were no significant group differences in payoff ($F_{(2,103)} = 0.22$, $p = 0.807$, $BF_{01} = 6.92$). Specifically, the mPFC-B group showed comparable performance to both HC and LC, suggesting that their ability to optimise strategic choices remained intact (HC vs mPFC-B: diff [95% CI] = 0.51 [-1.35 2.38], $p = 0.790$, $BF_{01} = 2.99$; LC vs mPFC-B: diff [95% CI] = 0.38 [-2.03 2.80], $p = 0.926$, $BF_{01} = 2.84$). Most importantly, the RT effect persisted. The mPFC-B group was significantly slower than both the HC group (diff [95% CI] = -0.28 [-0.50 -0.07], $p = 0.006$) and the LC group (diff [95% CI] = -0.30 [-0.58 -0.03], $p = 0.026$), with no difference between HC and LC (diff [95% CI] = -0.02 [-0.24 0.19], $p = 0.962$, $BF_{01} = 3.56$). Consistent with this, comparisons of the winning model parameters across groups yielded similar results to the main analysis (Figure S13). These results indicate that the retained mentalising and altered decision speed in the mPFC group are unlikely to be explained by compensation from an intact hemisphere.

Restricted the VLSM analysis to the focal mPFC lesion group

To further examine whether variability in lesion location within the mPFC itself was associated with model-derived results, we conducted an additional VLSM analysis restricted to the focal mPFC lesion group. This analysis specifically tests whether differences in damage distribution within the mPFC predict variability in model-derived parameters, independent of lesions outside this region.

Using the same preprocessing and statistical procedures as in the whole-brain analysis (rank transformation, permutation-based VLSM with TFCE), we examined associations

between voxel-wise damage and all five model parameters within the mPFC lesion group only. This analysis was restricted to voxels within the mPFC mask and therefore should be interpreted as a within-region analysis.

Within the mPFC-only analysis, no significant associations were observed for the higher-order mentalising parameter, influence play κ. In contrast, the simpler strategy, first-order belief-updating, fictitious play parameter η showed a significant association with damage to orbitofrontal cortex (OFC) within the mPFC regions (Figure S15A & S15B; $p < 0.01$; peak MNI coordinate [11, 24, -22], cluster size k = 245; peak MNI coordinate [10, 37, -25], cluster size k = 58). For decision-related DDM parameters, within the mPFC, damage to the orbitofrontal cortex (OFC) and extending to anterior prefrontal cortex (aPFC) was associated with higher drift rate temperature d(Figure S15C & S15D; $p < 0.01$; peak MNI coordinate [9, 62, 6], cluster size k = 1,275; peak MNI coordinate [1, 41, -16], cluster size k = 1,192) and non-decision time τ (Figure S15E & S15F; $p < 0.01$; peak MNI coordinate [2, 61, -1], cluster size k = 47), while no significant associations were observed for decision boundary α after stringent correction.

Notably, an association between fictitious play and damage to lateral orbital regions emerged only in the mPFC-only VLSM analysis and not in the conservative whole-brain analysis. This pattern suggests that first-order belief updating may depend on more fine-grained subregional variation within the mPFC [29], rather than reflecting a robust voxel-level effect detectable across heterogeneous lesion sites. Importantly, no associations were observed between any lesion region and the influence play parameter κ across either the whole-brain or mPFC-only analyses. This absence of lesion–symptom mapping effects for influence play κ is consistent with the behavioural findings of preserved higher-order mentalising following mPFC damage and suggests that social strategic reasoning can be maintained despite focal disruption to mPFC subregions.

In contrast, associations between decision boundary and mPFC damage were observed in the whole-brain VLSM analysis but were not consistently detected in the mPFC-only analysis. This difference likely reflects the distinct goals of the two approaches. The whole-brain analysis provides a conservative test of voxel-level lesion–behaviour associations beyond the general effects of having a brain lesion, whereas the mPFC-only analysis

examines relative variability within an already focal lesion group. Taken together, these findings support a robust role of mPFC damage in altered decision thresholds during strategic interactions, while suggesting that subregional variability within the mPFC may play a more limited role for this parameter.

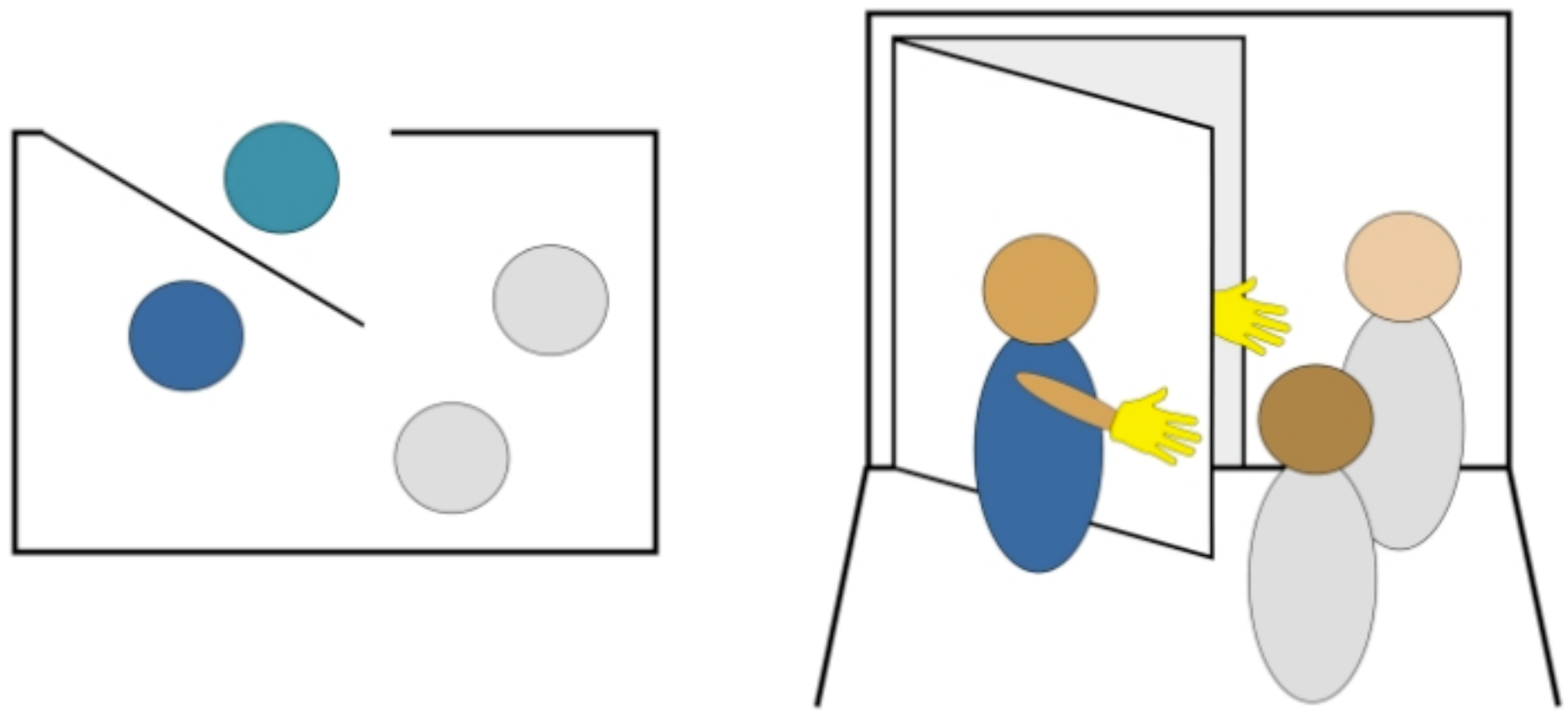

Figure S1. Participants took part in a role-assignment procedure to ensure they believed they were interacting with a real other person. Anonymously, the participant (dark blue) was introduced to a confederate researcher (light blue) by two experimenters (grey). Left panel: the positioning of the individuals from above. The participant and confederate researcher were positioned on either side of a door, preventing direct visual contact while allowing them to observe both experimenters. Right panel: the participant and confederate wore rubber gloves and were instructed to wave at each other while maintaining silence. Prior to the introduction, the experimenter informed the participants that they would be assigned the role of 'employer' or 'employee' in the inspection game (see Figure 2A for the game design). In reality, the participant was consistently assigned the role of 'employee'. Figure adapted from Jo et al. (2021)[4], licensed under the Creative Commons Attribution 4.0 International License (CC BY 4.0).

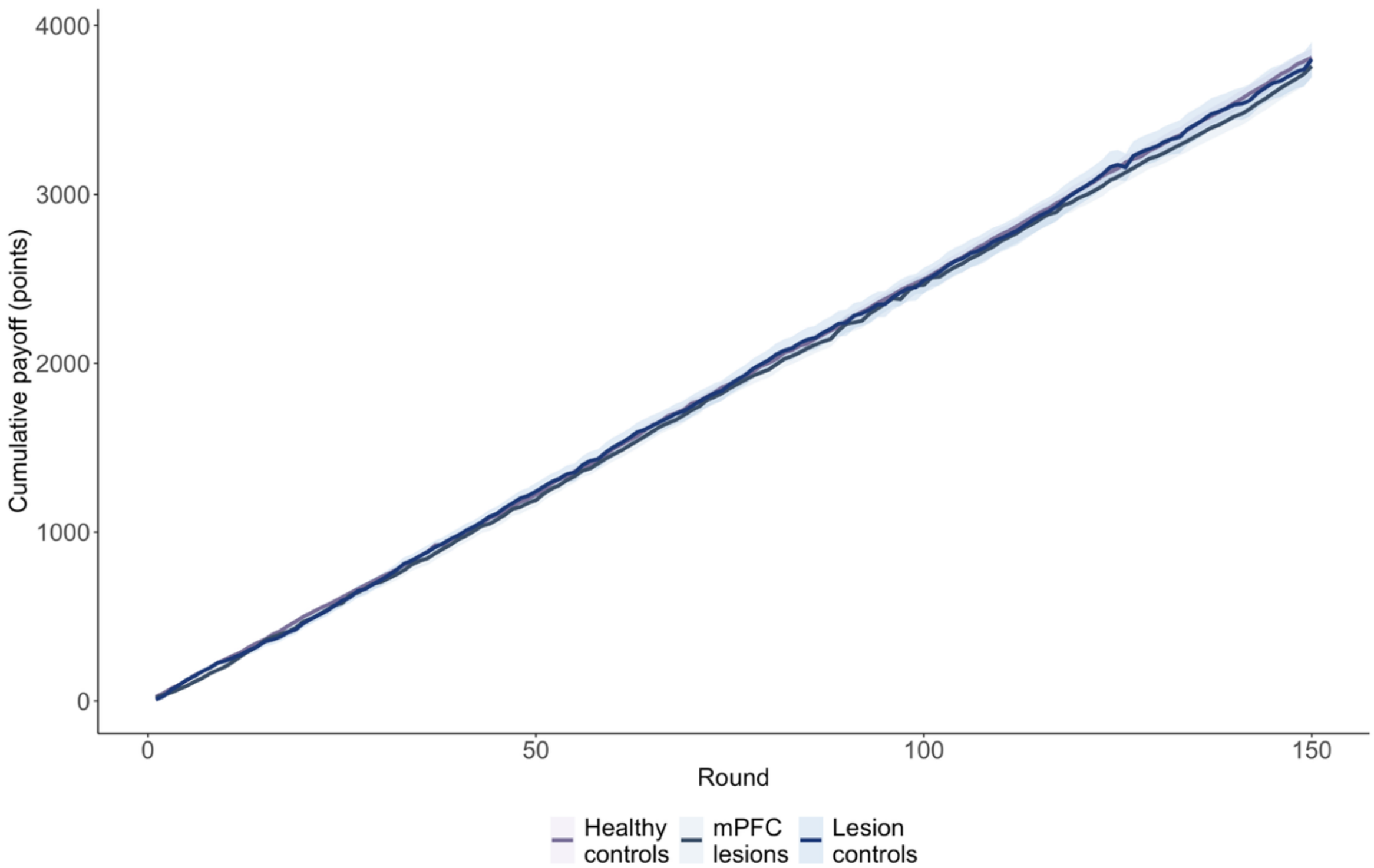

Figure S2. Cumulative payoff over rounds for each group. Mean cumulative payoff across rounds is shown separately for healthy controls, mPFC lesion patients, and lesion control participants. Shaded areas represent ±1 SEM. The trajectories show largely overlapping patterns across groups, consistent with the absence of group differences in overall payoff reported in the main text (see Figure 2C for the main payoff analysis). HC (n = 73), mPFC (n = 33), LC (n = 16).

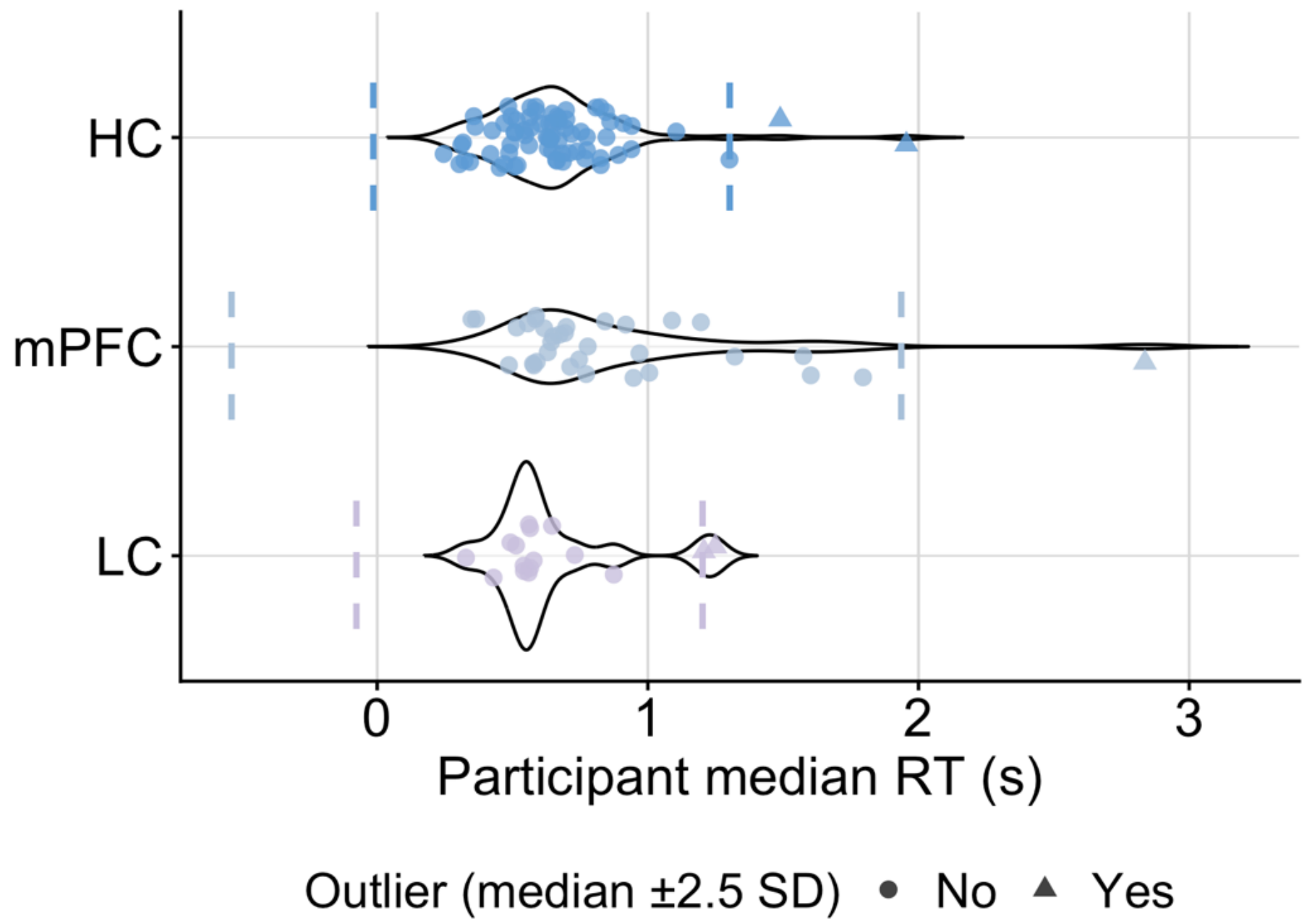

Figure S3. Distribution of participants' median response times across groups. Violin plots show the distribution of median response times (RTs) for individual participants in each group. Circles indicate participants whose median RT fell within a conservative ±2.5 SD range relative to the group median, whereas triangles indicate participants whose median RT exceeded this criterion. Vertical dashed lines denote the corresponding cut-off values (group median ±2.5 SD) calculated separately for each group. A small number of participants exceeded a conservative 2.5 SD criterion within their respective groups; however, these individuals were present across all groups (see Figure 2D for the main RT analysis). HC: healthy control group (n = 73); mPFC: mPFC lesion patients (n = 33); LC: lesion control patients (n = 16).

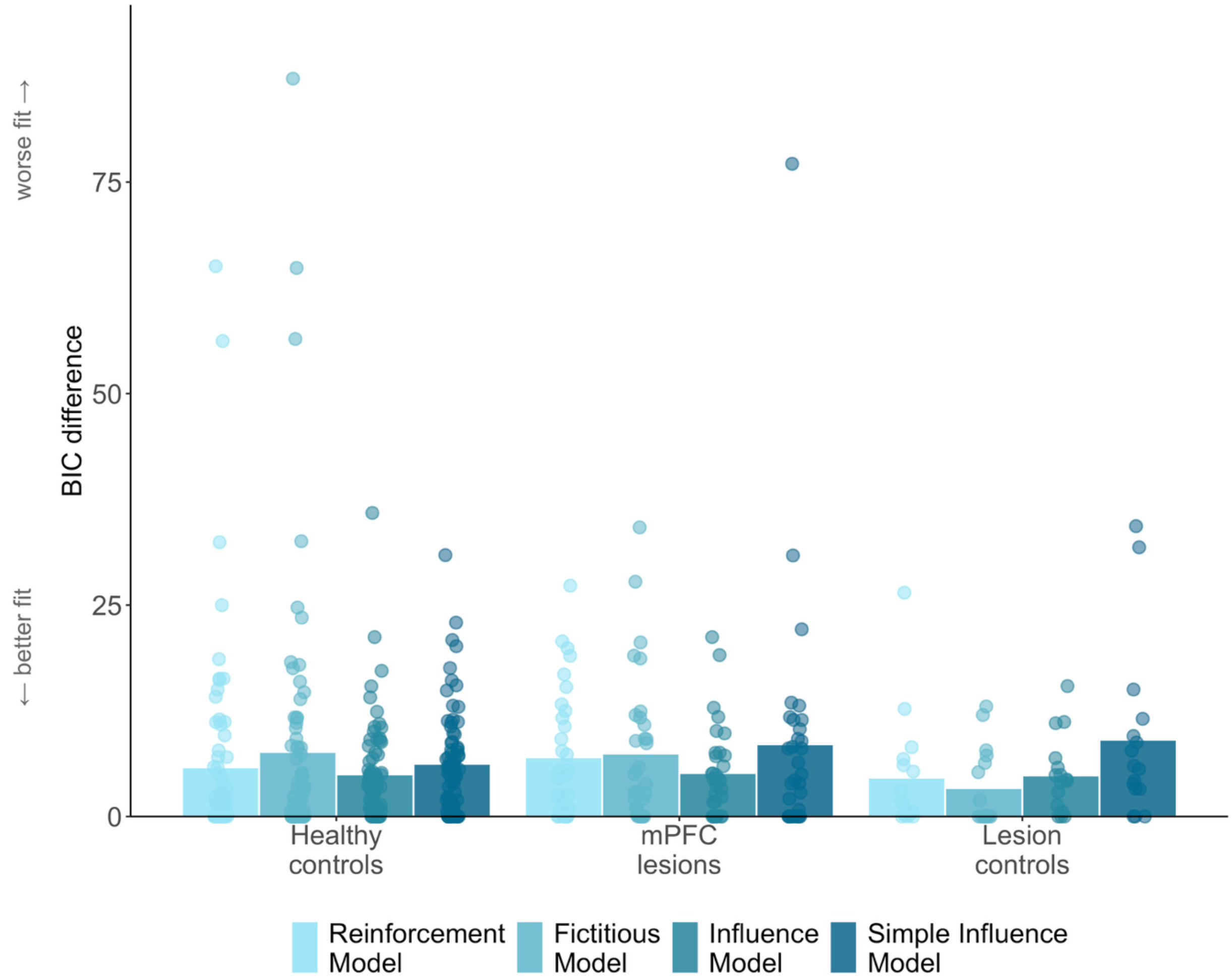

Figure S4. Relative BIC differences for model comparison across groups with individual data (coloured dots). Model comparison using BIC across groups indicated that the influence-DDM model generally provided the best fit for both the healthy control group and the mPFC lesion group (see also Figure 3A & 3C), whereas the fictitious play model tended to provide the best fit for the lesion control group. For each participant, relative BIC difference values were computed by subtracting the lowest BIC value among all candidate models from that participant's BIC values. Therefore, the model with the lowest BIC for a given participant was assigned a relative BIC difference of 0, and larger relative BIC differences indicated worse model fit. Because the best-fitting model could vary across individuals, the zero point in the relative BIC scale did not necessarily correspond to the same model across participants. The distinct pattern observed in the lesion control group might be related to its smaller sample size (n=16); HC ($n = 73$), mPFC ($n = 33$).

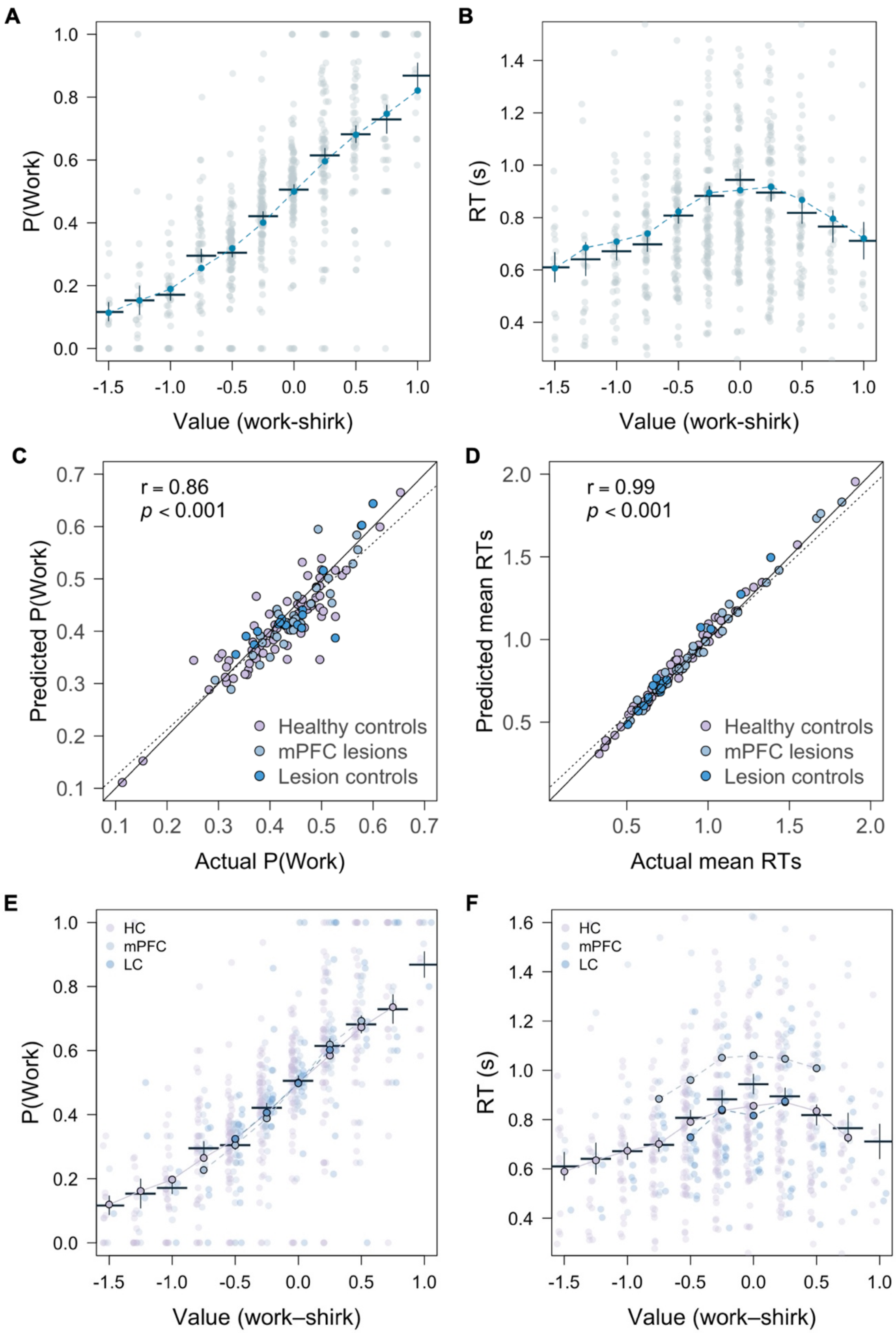

Figure S5. Model accurately reproduces choice and RT patterns across participants.

The posterior predictive check of the winning influence mode shows that the model can accurately predict (A) the participant's choice and (B) their response time (RT). Transparent light blue dots represent binned trial-level behavioural data averaged within participants, with the black line indicating the group-level average across participants and their error bars (error bars indicate ±1 SEM). Solid points show the group-level average of the model-simulated data across participants. (C) The predictions of the fitted influence learning model for each participant show a robust positive correlation with the observed probabilities of choosing work, P(Work) (two-sided Spearman correlation, $\rho(120) = 0.859$, 95% CI [0.77, 0.92], $p = 1.11 \times 10^{-36}$), and (D) the response times (RTs) (two-sided Spearman correlation, $\rho(120) = 0.988$, 95% CI [0.98, 0.99], $p = 8.93 \times 10^{-99}$). (see Figure 3B for parameter recovery results). (E-F) The same posterior predictive checks as (A-B) shown separately for each group (HC, mPFC, LC), with group membership indicated by colour as shown in the figure. p = p-value. HC (n = 73), mPFC (n = 33), LC (n = 16).

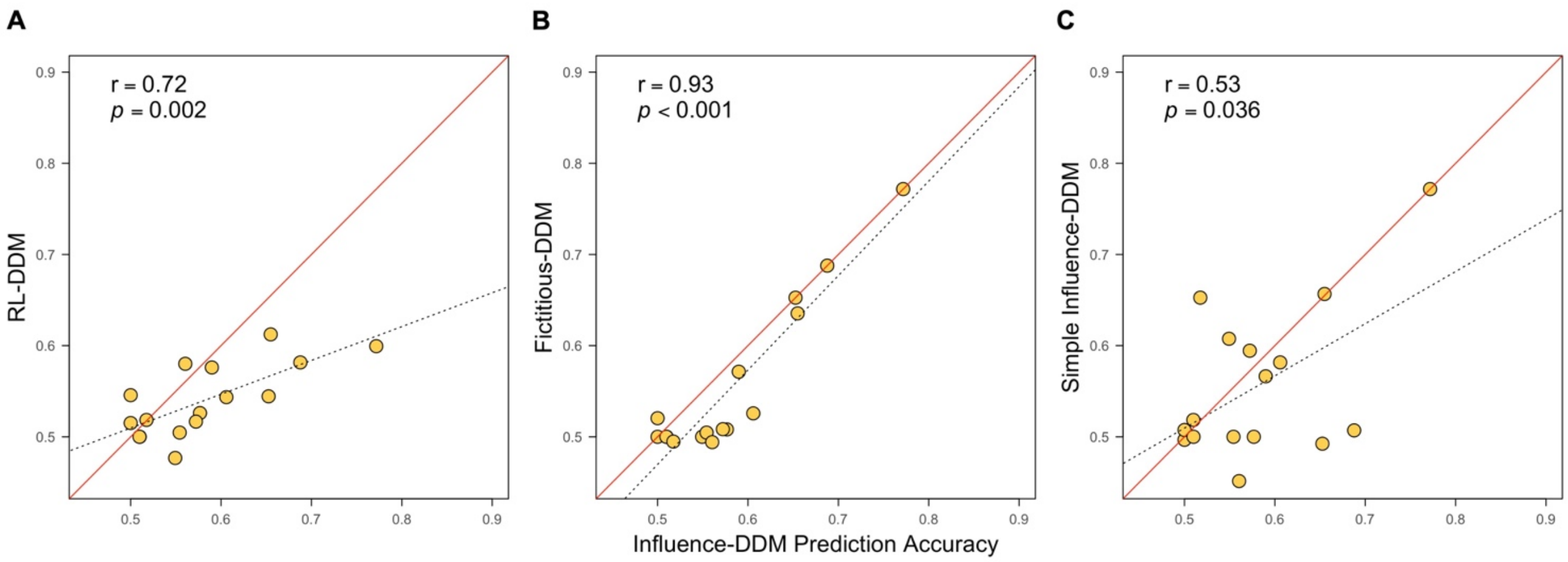

Figure S6. Predictive accuracy comparison between alternative models and the influence-DDM in the lesion control (LC) group (*n* = 16)**.** Scatter plots show participants' trial-by-trial predictive accuracy of the influence-DDM (x-axis) compared with (A) the reinforcement-DDM (two-sided Pearson correlation, $r_{(14)}$ = 0.716, 95% CI [0.34, 0.89], *p* = 0.002), (B) the fictitious-DDM (two-sided Pearson correlation, $r_{(14)}$ = 0.932, 95% CI [0.81, 0.98], *p* = 1.43 x 10^{-7}), and (C) the simple influence-DDM in the lesion control group (two-sided Pearson correlation, $r_{(14)}$ = 0.526, 95% CI [0.04, 0.81], *p* = 0.036) (See Figure 3C & S4 for the BIC difference of each group). Each point represents an individual participant. The red diagonal line indicates equal predictive accuracy between the two models being compared. The black dotted line indicates the best-fitting linear regression line between the predictive accuracies of the two models across participants. Points below the diagonal indicate higher predictive accuracy for the influence-DDM. Pearson correlation coefficients (r) and corresponding p-values are reported in each panel.

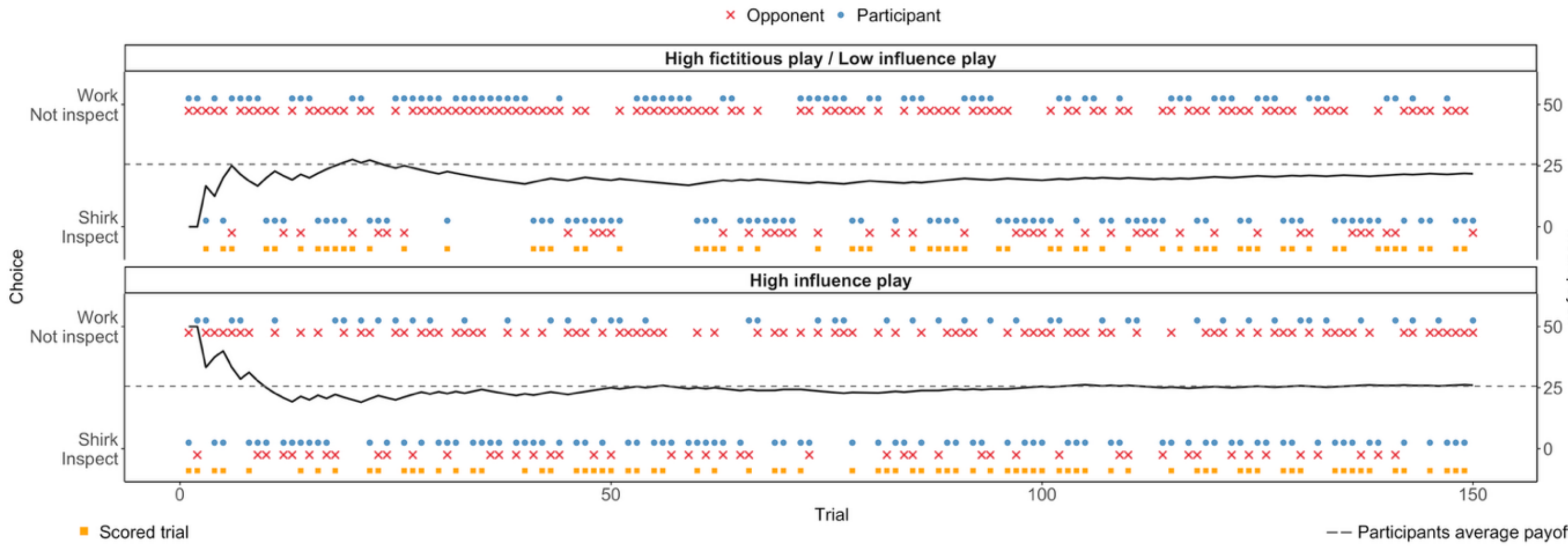

Figure S7. Illustrative example trial sequences showing different patterns of fictitious play and influence play of fitted influence-DDM. Example sequences of rounds are shown for two healthy control participants whose behaviour was characterised by high fictitious play but low influence play (top panel) and by high influence play (bottom panel), based on model-derived parameter estimates (see Figure 3 for main model fitting results). These examples are provided for illustrative purposes to aid intuition about how different mentalising strategies unfold across rounds and are not intended for statistical comparison. Note that higher influence plays leads to more alternation in choices. The x-axis represents trial number, illustrating the temporal sequence of decisions across the task. For each trial, blue circles indicate the participant's choices and red crosses indicate the opponent's choices (work vs. shirk / inspect vs. not inspect, as labelled on the y-axis). Yellow squares indicate trials on which the participant received a payoff, providing a visual marker of rewarded outcomes. The black line represents the participant's running average payoff, while the dashed grey line indicates the mean payoff across participants.

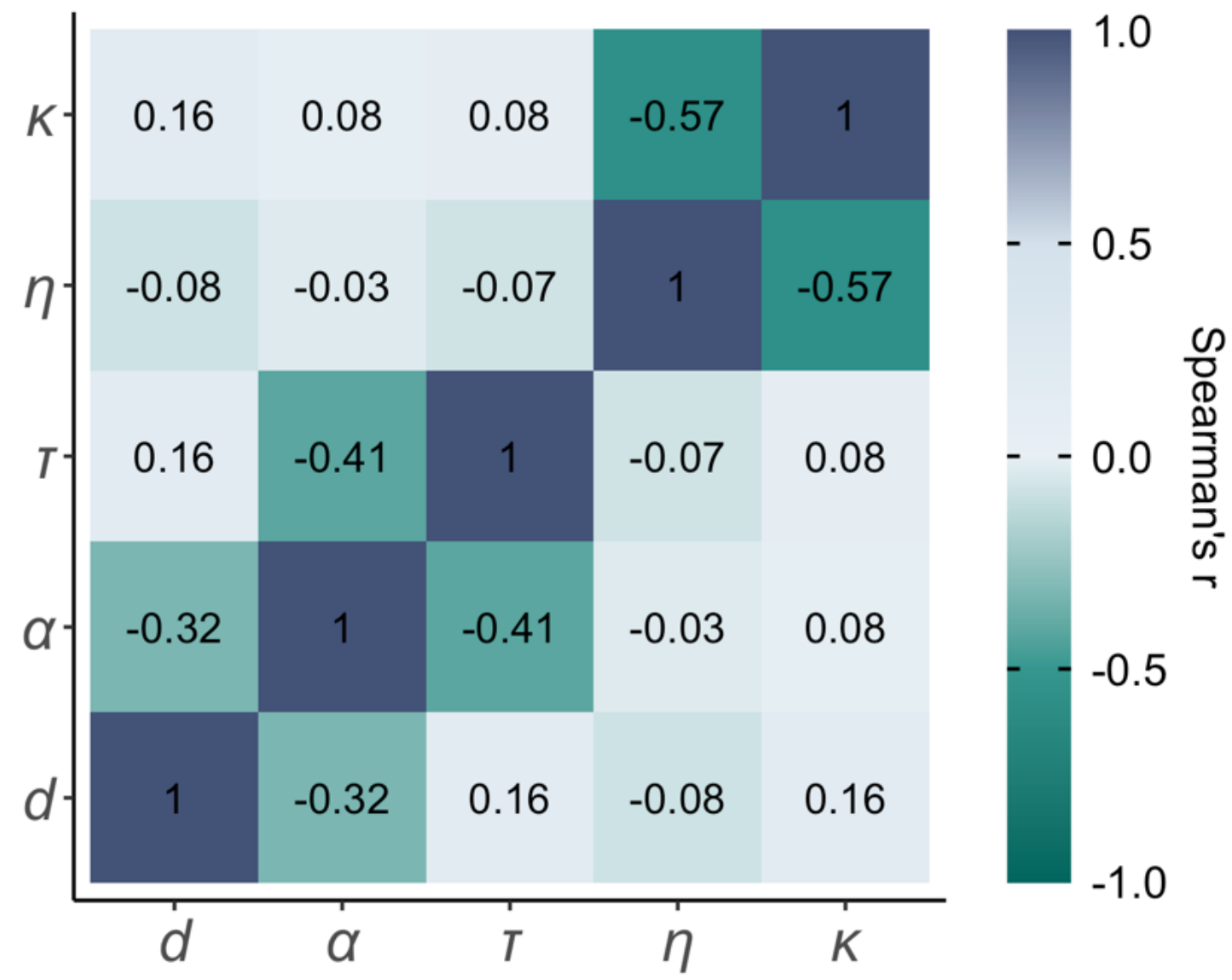

Figure S8. Correlation matrix of model parameters. Heatmap showing pairwise Spearman correlations between the five model parameters: drift rate temperature (d), decision boundary (α), non-decision time (τ), fictitious play (η), and influence play (κ) (see Figure 3 for main model fitting results). Values indicate correlation coefficients, with colour intensity reflecting correlation strength. Overall, correlations were low to modest, indicating that the parameters capture largely independent aspects of behaviour. Multicollinearity among the three DDM parameters was further assessed using variance inflation factors.

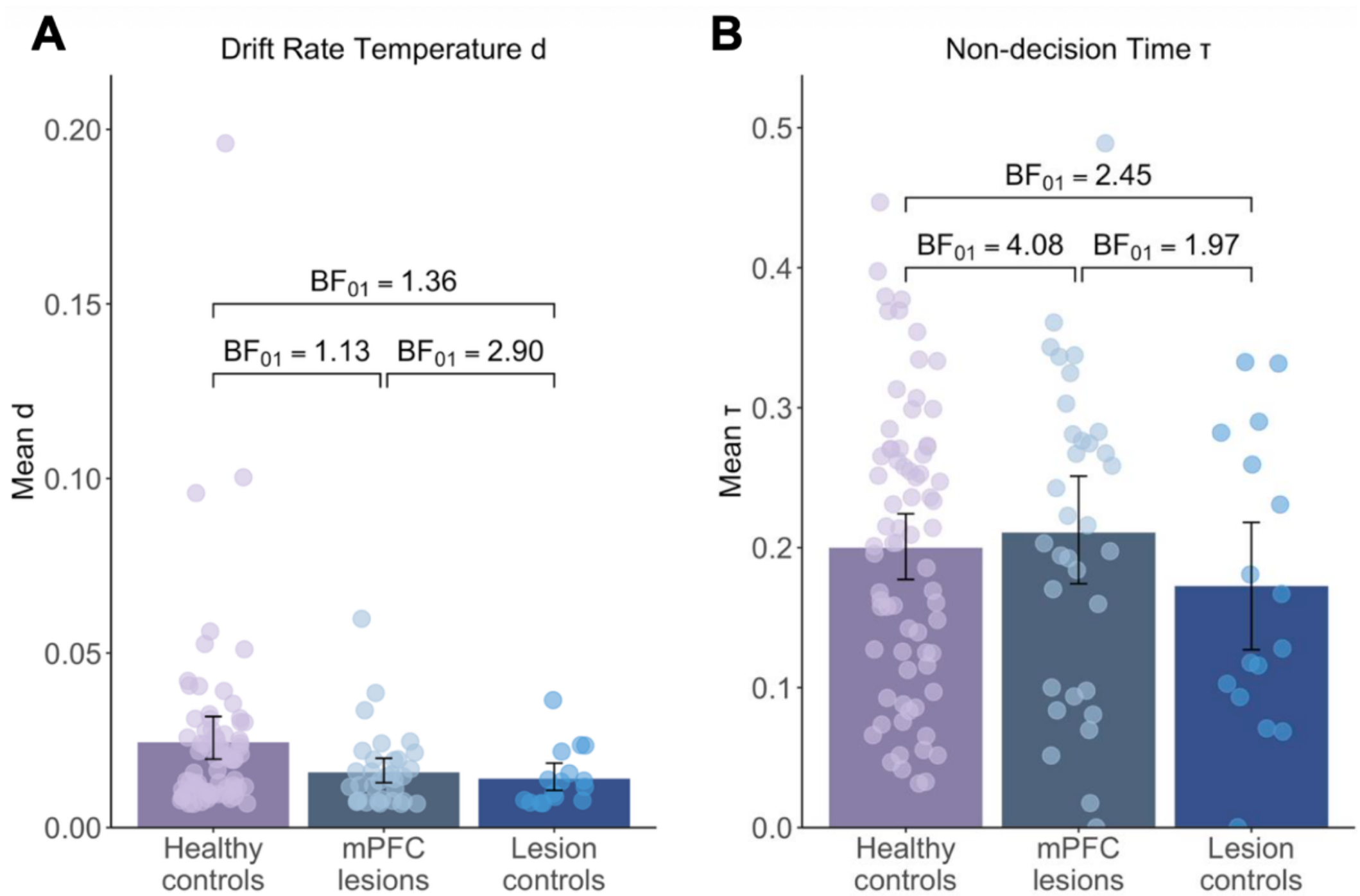

Figure S9. Patients with mPFC damage showed no significant difference in the decision process other than mentalising and evidence accumulation. (A) The drift rate from the winning model was comparable between groups. (B) The non-decision time τ was comparable between groups (see Figure 4 for main interest parameters). Group differences were assessed using a one-way ANOVA (two-sided) followed by Tukey-adjusted post hoc comparisons, together with Bayesian ANOVA and Bayesian t-tests. Each coloured dot represents an individual participant (HC: n = 73; mPFC: n = 33; LC: n = 16). Bars indicate group means, and error bars represent 95% bootstrap confidence intervals. p = p-value, BF_{01} = Bayes factor for null hypothesis.

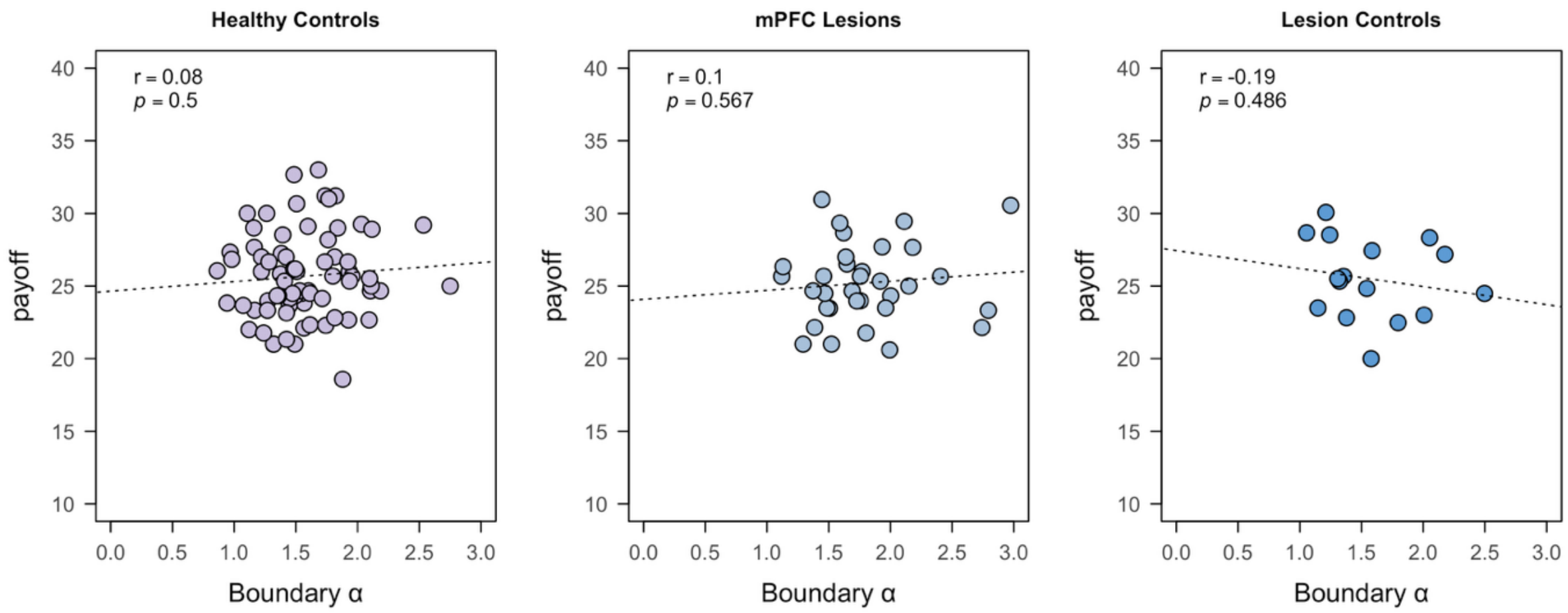

Figure S10. Relationship between fitted decision boundary and payoff across groups. Scatter plots show the relationship between fitted decision boundary (α) and payoff for (left) healthy controls (two-sided Pearson correlation, $r_{(71)}$ = 0.801, 95% CI [-0.15, 0.31], p = 0.500), (middle) mPFC lesion patients (two-sided Pearson correlation, $r_{(31)}$ = 0.103, 95% CI [-0.25, 0.43], p = 0.567), and (right) lesion control participants (two-sided Pearson correlation, $r_{(14)}$ = -0.188, 95% CI [-0.63, 0.34], p = 0.486). Each point represents an individual participant (HC: n = 73; mPFC: n = 33; LC: n = 16). Pearson correlation coefficients (r) and corresponding p-values are shown in each panel. No significant association between the decision boundary and payoff was observed in any group (see Figure 4D for decision boundary comparisons across groups).

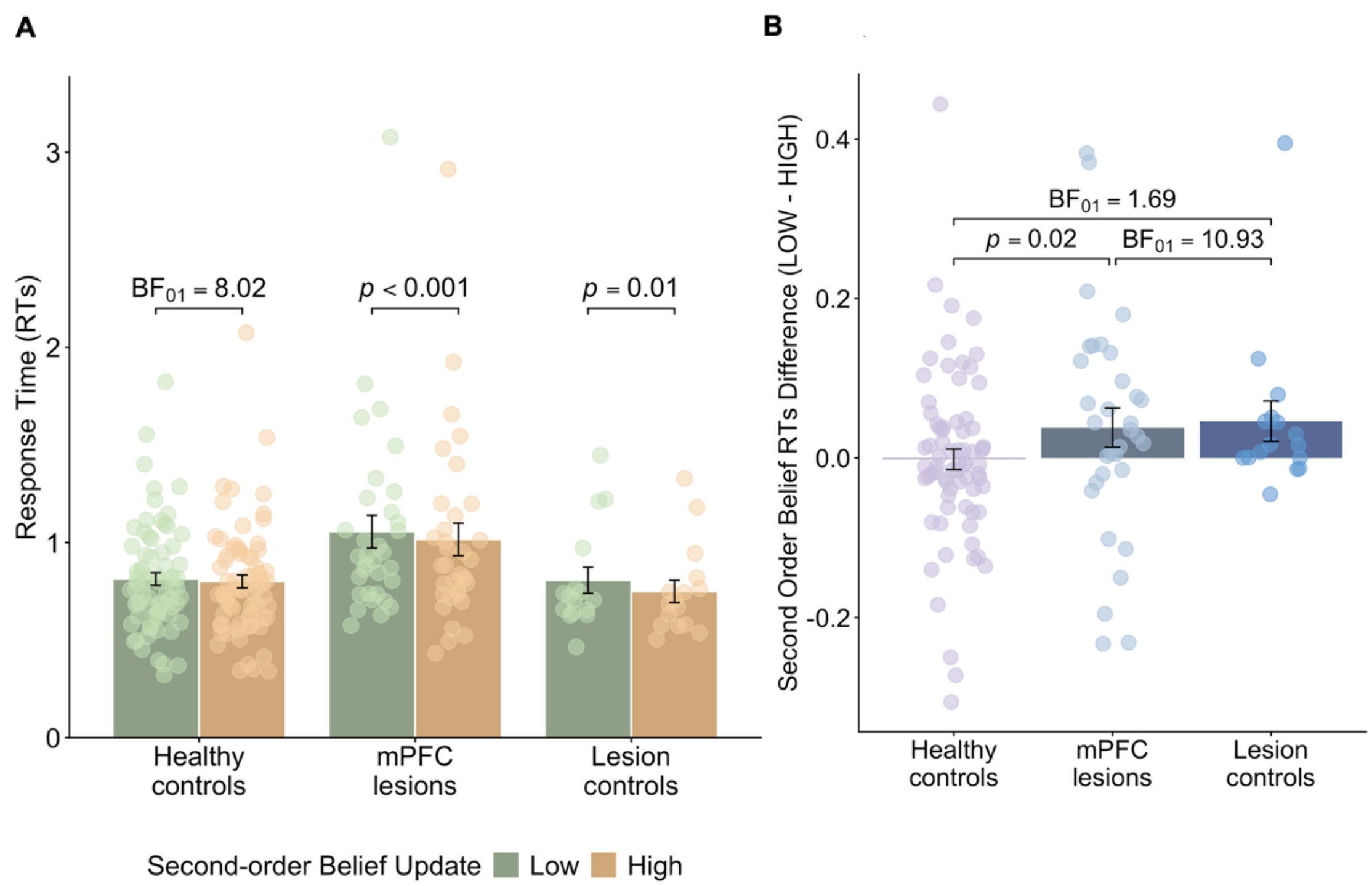

Figure S11. Response times across second-order belief update conditions and each group's response times difference. Statistical comparisons were performed using robust linear mixed-effects models predicting response time from second-order belief update magnitude (high vs low), group, previous trial outcome (success vs failure), and their interactions, with participant included as a random intercept. (A) Response times (RTs) for each update condition (high and low) across groups. The mPFC and LC groups showed significantly faster RTs in high compared to low update trials (mPFC: $z = -3.34$, $p = 0.0008$, $BF_{01} = 0.04$; LC: $z = -2.54$, $p = .011$, $BF_{01} = 0.44$), whereas the HC group showed no significant difference ($z = -0.80$, $p = 0.424$, $BF_{01} = 8.02$). (B) RT differences between high and low update (low update minus high update) across groups. Pairwise comparisons of update-related RT differences showed a larger low–high RT difference in the mPFC compared with HC group ($z = 2.306$, $p = 0.021$, $BF_{01} = 0.77$). The mPFC–LC comparison provided evidence for no difference ($z = -0.145$, $p = 0.885$, $BF_{01} = 10.93$), whereas the HC–LC comparison was inconclusive ($z = -1.938$, $p = 0.053$, $BF_{01} = 1.69$). Each coloured dot represents an individual subject (HC: $n = 73$; mPFC: $n = 33$; LC: $n = 16$). Bars show group means; error bars denote ±1 standard error of the mean (SEM). p = p-value, BF_{01} = Bayes factor for null hypothesis. Bayes factors were approximated from the z-statistics using a BIC approximation.

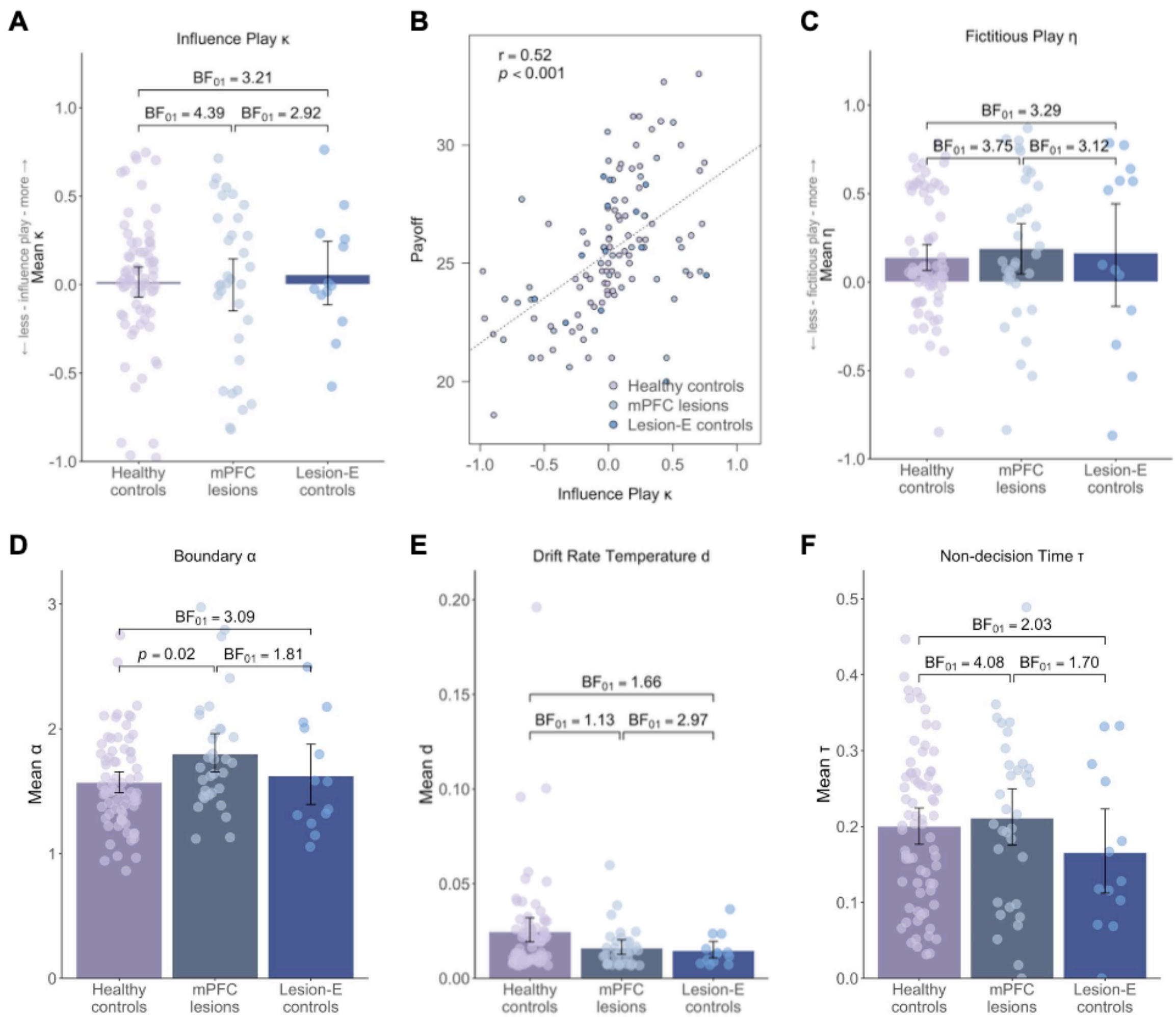

Figure S12. Group comparisons of the winning model parameters remained consistent with the main analysis, even after excluding lesion control participants with TPJ damage. Panels show (A) influence play κ ($F_{(2,116)}$ = 0.12, p = 0.888, η^2 = 0.002, BF_{01} = 8.96; mPFC vs HC: diff [95% CI] = -0.02 [-0.22 0.17], p = 0.955, BF_{01} = 4.39; LC vs HC: diff [95% CI] = 0.04 [-0.24 0.32], p = 0.945, BF_{01} = 3.21; LC vs mPFC: diff [95% CI] = 0.06 [-0.24 0.36], p = 0.881, BF_{01} = 2.92), (B) its correlation with task payoff (two-sided Pearson correlation, $r_{(117)}$ = 0.519, 95% CI [0.37, 0.64], p = 1.47 × 10^{-9}), (C) fictitious play η ($F_{(2,116)}$ = 0.20, p = 0.819, η^2 = 0.003, BF_{01} = 8.22; mPFC vs HC: diff [95% CI] = 0.05 [-0.14 0.24], p = 0.806, BF_{01} = 3.75; LC vs HC: diff [95% CI] = 0.03 [-0.25 0.30], p = 0.972, BF_{01} = 3.29; LC vs mPFC: diff [95% CI] = -0.02 [-0.32 0.27], p = 0.980, BF_{01} = 3.12), (D) decision boundary α ($F_{(2,116)}$ = 3.71, p = 0.027, η^2 = 0.060, BF_{01} = 0.45; mPFC vs HC: diff [95% CI] = 0.23 [0.03 0.43], p = 0.020, BF_{01} = 0.17; LC vs HC: diff [95% CI] = 0.05 [-0.23 0.34], p = 0.899, BF_{01} = 3.09; LC vs mPFC: diff [95% CI] = -0.17 [-0.48 0.14], p = 0.377,

BF_{01} = 1.81), (E) drift rate temperature d ($F_{(2,116)}$ = 0.22, p = 0.097, η^2 = 0.039, BF_{01} = 1.35; mPFC vs HC: diff [95% CI] = -0.01 [-0.02 0.01], p = 0.152, BF_{01} = 1.13; LC vs HC: diff [95% CI] = -0.01 [-0.03 0.01], p = 0.292, BF_{01} = 1.66; LC vs mPFC: diff [95% CI] = -0.01 [-0.02 0.02], p = 0.981, BF_{01} = 2.97), and (F) non-decision time τ ($F_{(2,116)}$ = 0.88, p = 0.420, η^2 = 0.015, BF_{01} = 5.28; mPFC vs HC: diff [95% CI] = 0.01 [-0.04 0.06], p = 0.874, BF_{01} = 4.08; LC vs HC: diff [95% CI] = -0.04 [-0.11 0.04], p = 0.521, BF_{01} = 2.03; LC vs mPFC: diff [95% CI] = -0.05 [-0.13 0.04], p = 0.386, BF_{01} = 1.70) (see Figure 4 and Figure S4 for main analyses). Each coloured dot represents an individual subject (HC: n = 73; mPFC: n = 33; LC: n = 13). Bars indicate group means, and error bars represent 95% bootstrap confidence intervals. p = p-value, BF_{01} = Bayes factor for null hypothesis. Group differences in continuous variables were assessed using one-way analysis of variance (ANOVA) followed by Tukey's honestly significant difference (HSD) post hoc tests with adjustment for multiple comparisons. All statistical tests were two-sided.

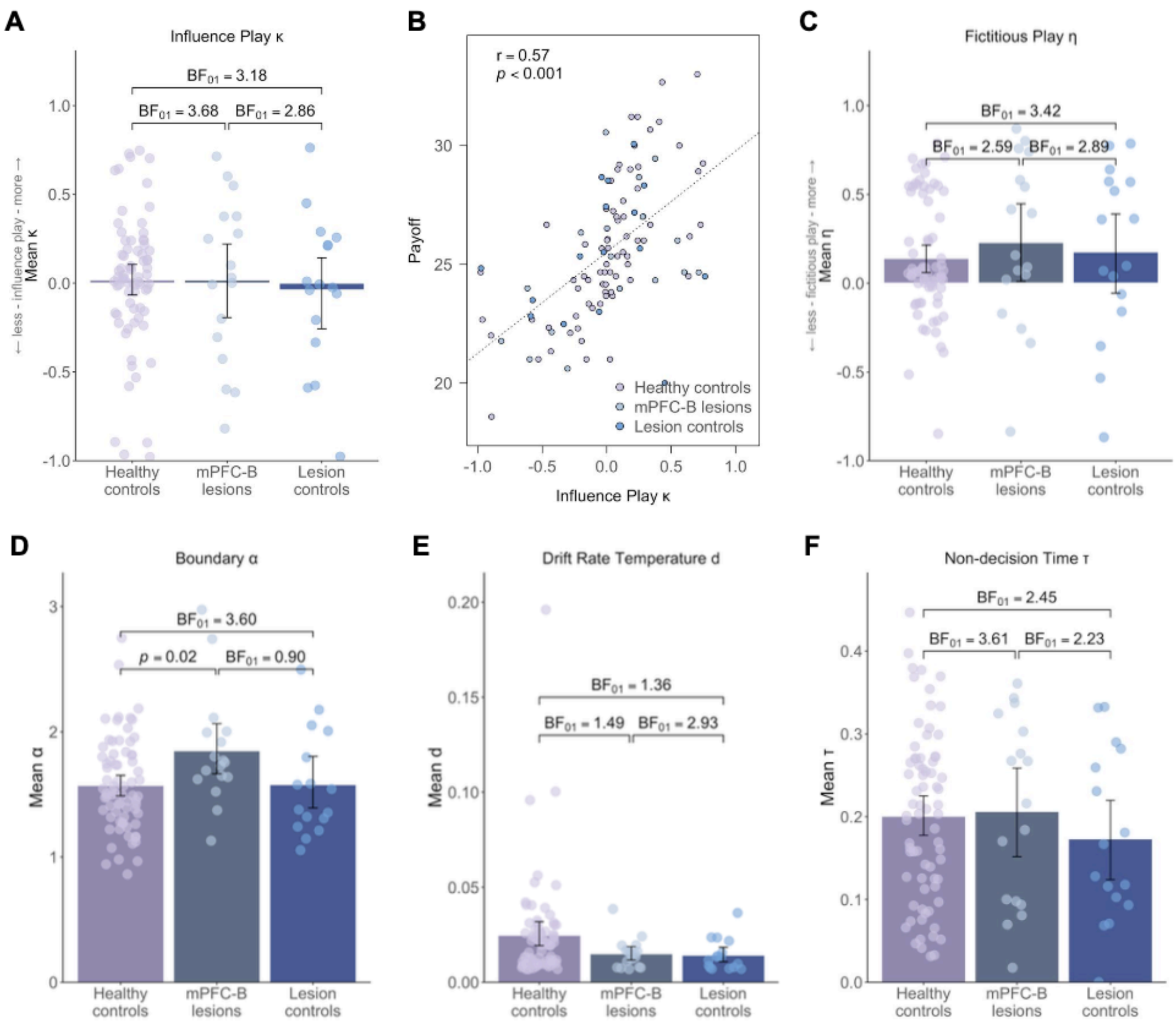

Figure S13. Restricting the mPFC group to bilateral lesions yielded model parameter results consistent with the main analysis. Panels show (A) influence play κ ($F_{(2,103)}$ = 0.15, p = 0.864, η^2 = 0.003, BF_{01} = 7.63; mPFC vs HC: diff [95% CI] = -0.01 [-0.25 0.25], p = 1.000, BF_{01} = 3.68; LC vs HC: diff [95% CI] = -0.06 [-0.32 0.20], p = 0.855, BF_{01} = 3.18; LC vs mPFC: diff [95% CI] = -0.06 [-0.38 0.27], p = 0.910, BF_{01} = 2.86), (B) its correlation with task payoff (two-sided Pearson correlation, $r_{(104)}$ = 0.570, 95% CI [0.43, 0.69], p = 1.87 × 10^{-10}), (C) fictitious play η ($F_{(2,103)}$ = 0.40, p = 0.674, η^2 = 0.008, BF_{01} = 6.21; mPFC vs HC: diff [95% CI] = 0.09 [-0.16 0.33], p = 0.660, BF_{01} = 2.59; LC vs HC: diff [95% CI] = 0.04 [-0.21 0.29], p = 0.937, BF_{01} = 3.42; LC vs mPFC: diff [95% CI] = -0.05 [-0.37 0.26], p = 0.916, BF_{01} = 2.89), (D) decision boundary α ($F_{(2,103)}$ = 3.63, p = 0.030, η^2 = 0.066, BF_{01} = 0.55; mPFC vs HC: diff [95% CI] = 0.28 [0.03 0.52], p = 0.025, BF_{01} = 0.19; LC vs HC: diff [95% CI] = 0.01 [-0.25 0.26], p = 0.998, BF_{01} = 3.60; LC vs mPFC: diff [95% CI] = -0.27 [-0.59 0.05], p = 0.115, BF_{01} = 0.90), (E) drift rate temperature d ($F_{(2,103)}$ = 2.23,

p = 0.112, η^2 = 0.042, BF_{01} = 1.39; mPFC vs HC: diff [95% CI] = -0.01 [-0.02 0.01], p = 0.256, BF_{01} = 1.49; LC vs HC: diff [95% CI] = -0.01 [-0.03 0.01], p = 0.224, BF_{01} = 1.36; LC vs mPFC: diff [95% CI] = -0.01 [-0.02 0.02], p = 0.995, BF_{01} = 2.93), and (F) non-decision time τ ($F_{(2,103)}$ = 0.53, p = 0.588, η^2 = 0.015, BF_{01} = 5.81; mPFC vs HC: diff [95% CI] = 0.01 [-0.06 0.07], p = 0.976, BF_{01} = 3.61; LC vs HC: diff [95% CI] = -0.03 [-0.10 0.04], p = 0.607, BF_{01} = 2.45; LC vs mPFC: diff [95% CI] = -0.03 [-0.12 0.05], p = 0.629, BF_{01} = 2.23) (see Figure 4 and Figure S4 for main analyses). Each coloured dot represents an individual subject (HC: n = 73; mPFC: n = 17; LC: n = 16). Bars indicate group means, and error bars represent 95% bootstrap confidence intervals. p = p-value, BF_{01} = Bayes factor for null hypothesis. Group differences in continuous variables were assessed using one-way analysis of variance (ANOVA) followed by Tukey's honestly significant difference (HSD) post hoc tests with adjustment for multiple comparisons. All statistical tests were two-sided.

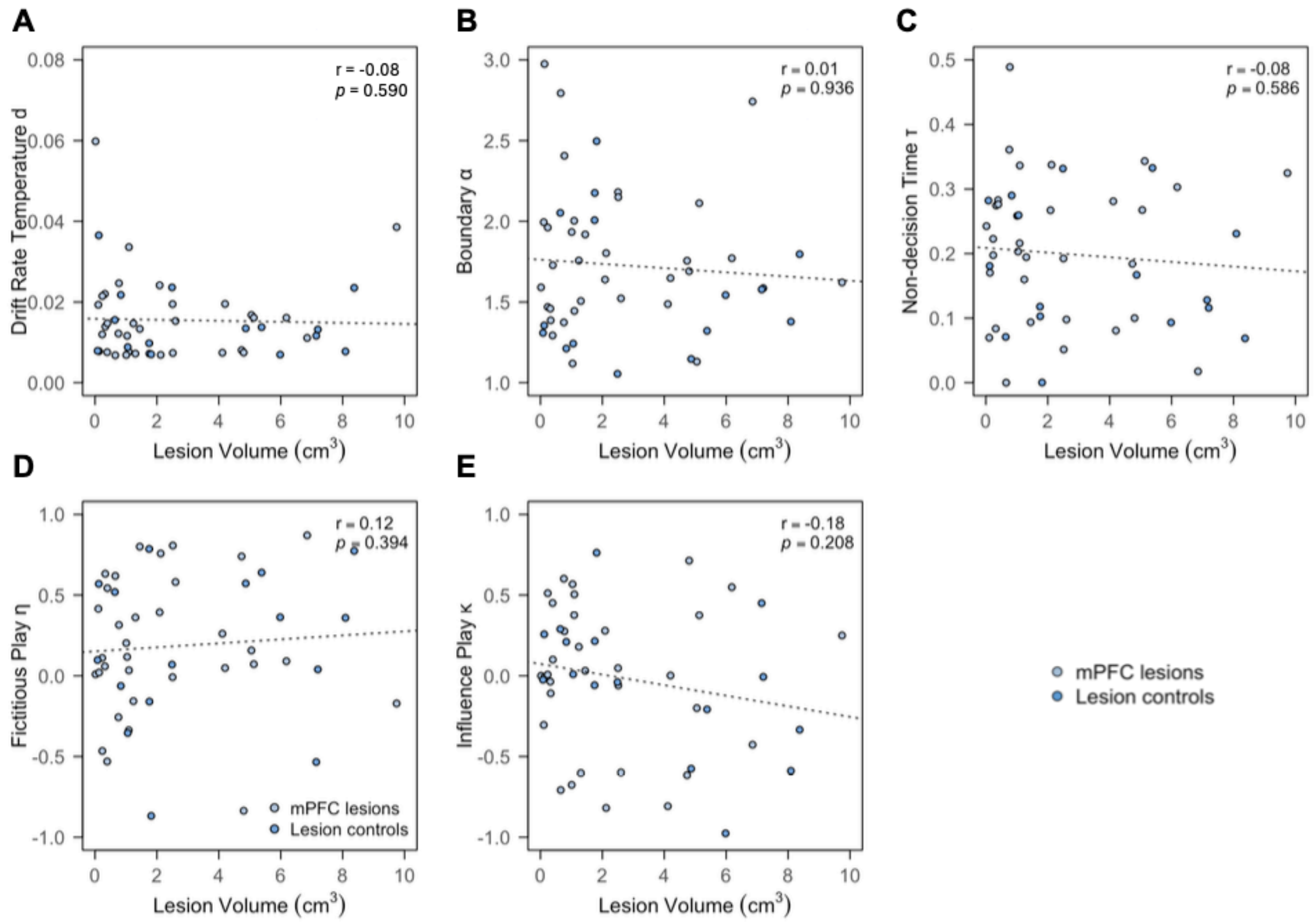

Figure S14. The Patients' lesion volume was not correlated with the influence-DDM model parameters used in VLSM analysis. There was no significant correlation between the overall lesion volume and any VLSM variables, (A) drift rate temperature d, (B) decision boundary α, (C) non-decision time τ, (D) fictitious play η and (E) influence play κ, in either of the two lesion patient groups. The results suggest that the model parameters were not directly affected by merely how severe the lesion is, and that the subsequent VLSM analysis could be a reasonable analysis to find out whether the specific lesion area is responsive to the model parameters (see Figure 5 for VLSM analysis results). Each coloured dot represents an individual subject (mPFC: *n* = 33; LC: *n* = 16). Pearson correlation (two-sided) coefficients (r) and corresponding p-values are reported in each panel.

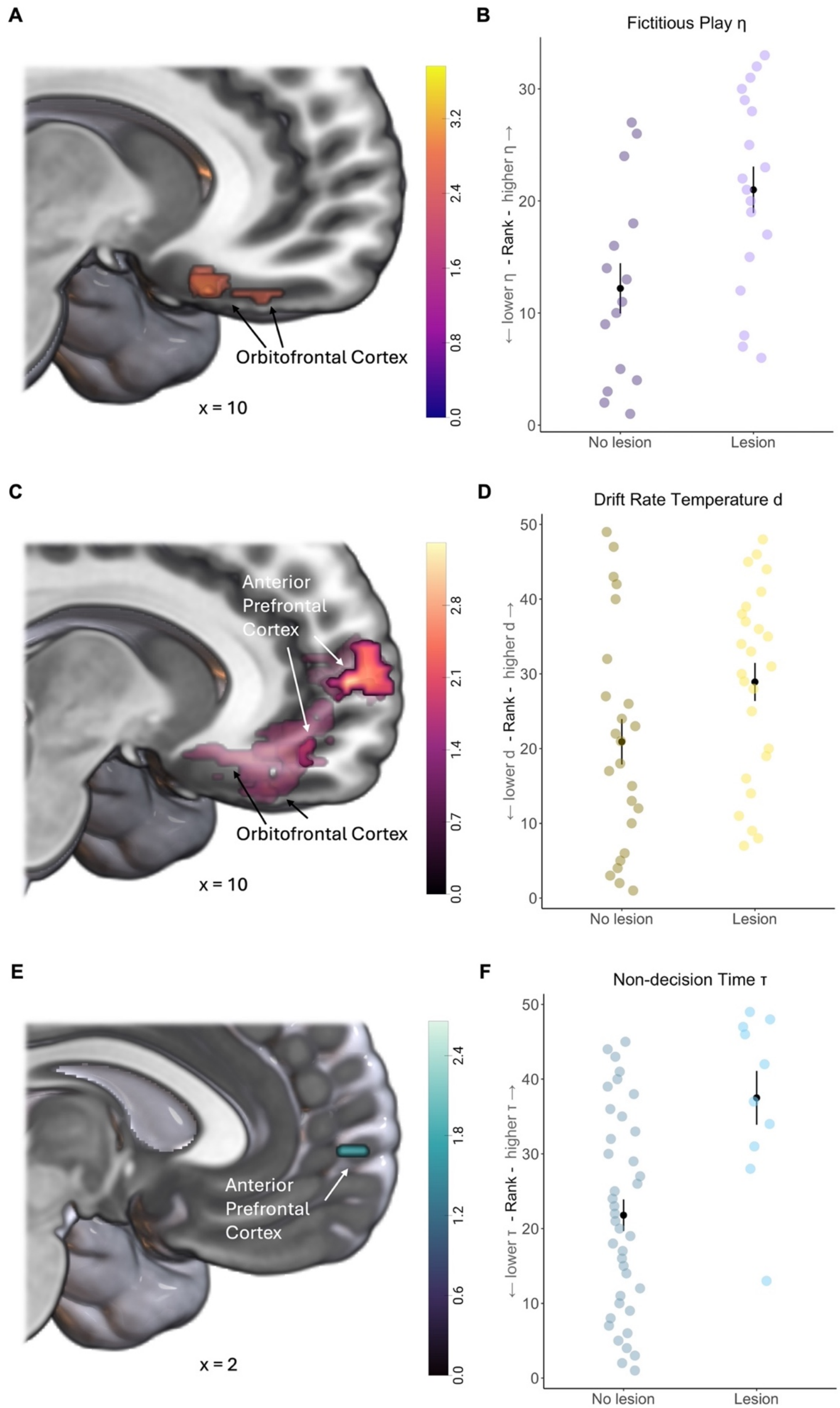
A
B
Fictitious Play η
Orbitofrontal Cortex
x = 10
← lower η - Rank - higher η →
No lesion
Lesion
C
D
Drift Rate Temperature d
Anterior Prefrontal Cortex
Orbitofrontal Cortex
x = 10
← lower d - Rank - higher d →
No lesion
Lesion
E
F
Non-decision Time τ
Anterior Prefrontal Cortex
x = 2
← lower τ - Rank - higher τ →
No lesion
Lesion

Figure S15. Damage to the orbitofrontal cortex (OFC) was associated with the first-order belief-updating, and damage to the orbitofrontal cortex (OFC) and anterior prefrontal cortex (aPFC) were associated with the DDM parameters. Permutation-based, within-mPFC, non-parametric voxel-based lesion-symptom mapping (VLSM) results of (A) fictitious play η , (C) drift rate temperature d, and (E) non-decision time τ. The ranked (B) fictitious play η (no lesion: $n = 15$; lesion: $n = 18$), (D) drift rate temperature d (no lesion: $n = 7$; lesion: $n = 26$), and (F) non-decision time τ (no lesion: $n = 18$; lesion: $n = 15$) contrasts of participants with or without damage in the areas identified by the mPFC-only VLSM analysis (see Figure 5 for whole brain analysis). $p < 0.01$ (the colour bar represents the t-value of the analysis). Note: Panels (B), (D), and (F) are for illustrative purposes only and display the ranked difference in model parameter contrasts between participants with vs. without lesions in the ROI defined by a whole-brain contrast. Each coloured dot represents an individual participant with mPFC lesion ($n = 33$). Black dots indicate group rank means, and error bars indicate mean ± SEM.

Table S1. Summary of demographic variables across groups

Variable	HC (*n* = 73) mean [SD]	mPFC (*n* = 33) mean [SD]	LC (*n* = 16) mean [SD]	Statistics	HC vs mPFC *p*-value	HC vs LC *p*-value	LC vs mPFC *p*-value
Age	60.53 [18.69]	56.88 [10.71]	56.06 [11.43]	$F_{(2,119)}$=1.18, p=0.415, η^2=0.01	0.526	0.574	0.985
Gender	47.9% M	48.5% M	31.3% M	$\chi^2_{(2)}$=1.59, p=0.451, Cramer's V=0.11	1.000	0.348	0.404
Edu	3.32 [0.84]	2.94 [1.11]	3.31 [1.25]	$F_{(2,116)}$=1.81, p=0.168, η^2=0.03	0.156	0.999	0.425
AMI	1.20 [0.45]	1.43 [0.60]	1.38 [0.63]	$F_{(2,116)}$=2.28, p=0.107, η^2=0.04	0.116	0.461	0.946
BDI	6.38 [7.27]	10.09 [7.56]	14.00 [11.20]	$F_{(2,116)}$=6.97, p=0.001, η^2=0.11	0.077	0.002	0.249
Trail A	28.57 [13.28]	26.99 [10.02]	30.82 [14.60]	$F_{(2,117)}$=0.49, p=0.617, η^2=0.01	0.831	0.798	0.592
Trail B	61.72 [25.88]	77.19 [56.50]	73.89 [34.66]	$F_{(2,117)}$=2.17, p=0.119, η^2=0.04	0.133	0.465	0.955
Raven	4.95 [2.36]	3.52 [2.51]	3.33 [2.41]	$F_{(2,116)}$=5.42, p=0.006, η^2=0.09	0.018	0.052	0.968
ACE	95.72 [3.91]	90.61 [8.55]	91.75 [6.35]	$F_{(2,104)}$=8.43, p=0.0004, η^2=0.14	0.001	0.052	0.810

Note. HC: Healthy controls, LC: Lesion controls, SD: standard deviation, Edu: Education scale (1-6), AMI: Apathy Motivation Index, BDI: Beck Depression Inventory, Trail A / B: Trail Making Test Part A / Part B, Raven: Raven Standard Progressive Matrices, ACE: Addenbrooke's Cognitive Examination, M: male. Missing data were excluded from the corresponding analyses. Edu, AMI, and BDI: HC (*n* = 71), mPFC (*n* = 32), LC (*n* = 16); Trail A/B and Raven: HC (*n* = 73), mPFC (*n* = 31), LC (*n* = 16); ACE: HC participants aged >40 years (*n* = 60), mPFC lesion patients (*n* = 31), and lesion controls (*n* = 16). Group differences in continuous variables were assessed using one-way analysis of variance (ANOVA) followed by Tukey's honestly significant difference (HSD) post hoc tests. Gender distributions were compared using chi-square tests. All statistical tests were two-sided.

Table S2. Robust linear mixed-effects model predicting RT after trimming extreme trials.

Parameters	Coefficient (β)	SE	CI low	CI high	t (df=16255)	*p*-value
(Intercept)	0.81	0.04	0.74	0.89	21.39	4.38×10^{-100}
Group (mPFC vs HC)	-0.12	0.05	-0.21	-0.03	-2.68	0.007
Group (mPFC vs LC)	-0.15	0.07	-0.28	-0.02	-2.25	0.025

Note. mPFC: mPFC lesion patients, HC: Healthy controls, LC: Lesion controls. Robust linear mixed-effects models were fitted to trimmed reaction time data. Coefficients represent fixed-effect estimates (β). All statistical tests were two-sided. P-values and confidence intervals (95% CI) were calculated using a Wald t-distribution approximation. No correction for multiple comparisons was applied. HC: $n = 73$; mPFC: $n = 33$; LC: $n = 16$.

Table S3. Robust linear mixed-effects model predicting RT after log-transformed RTs.

**Parameters**	**Coefficient (β)**	**SE**	**CI low**	**CI high**	**t** (df=18198)	***p*-value**
(Intercept)	-0.24	0.06	-0.35	-0.13	-4.33	1.49×10^{-05}
Group (mPFC vs HC)	-0.18	0.07	-0.31	-0.04	-2.59	0.010
Group (mPFC vs LC)	-0.22	0.10	-0.41	-0.02	-2.21	0.027

Note. mPFC: mPFC lesion patients, HC: Healthy controls, LC: Lesion controls. Robust linear mixed-effects models were fitted to log-transformed reaction time data. Coefficients represent fixed-effect estimates (β). All statistical tests were two-sided. P-values and confidence intervals (95% CI) were calculated using a Wald t-distribution approximation. No correction for multiple comparisons was applied. HC: $n = 73$; mPFC: $n = 33$; LC: $n = 16$.

Table S4. Robust linear mixed-effects model predicting RT.

**Parameters**	**Coefficient (β)**	**SE**	**CI low**	**CI high**	**t** (df=18198)	***p*-value**
(Intercept)	0.81	0.04	0.76	0.91	22.02	4.08×10^{-106}
Group (mPFC vs HC)	-0.12	0.05	-0.21	-0.03	-2.68	0.007
Group (mPFC vs LC)	-0.15	0.07	-0.28	-0.02	-2.27	0.024

Note. mPFC: mPFC lesion patients, HC: Healthy controls, LC: Lesion controls. Robust linear mixed-effects models were fitted to reaction time data. Coefficients represent fixed-effect estimates (β). All statistical tests were two-sided. P-values and confidence intervals (95% CI) were calculated using a Wald t-distribution approximation. No correction for multiple comparisons was applied. HC: *n* = 73; mPFC: *n* = 33; LC: *n* = 16.

Table S5. Robust linear mixed-effects model predicting RT excluding extreme participants.

Parameters	Coefficient (β)	SE	CI low	CI high	t (df=17456)	*p*-value
(Intercept)	0.81	0.03	0.75	0.88	23.59	3.59×10^{-121}
Group (mPFC vs HC)	-0.12	0.04	-0.20	-0.03	-2.79	0.005
Group (mPFC vs LC)	-0.18	0.06	-0.30	-0.05	-2.84	0.005

Note. mPFC: mPFC lesion patients, HC: Healthy controls, LC: Lesion controls. Robust linear mixed-effects models were fitted to reaction time data without participants whose median RT exceeded a conservative 2.5 SD criterion within their respective groups. Coefficients represent fixed-effect estimates (β). All statistical tests were two-sided. P-values and confidence intervals (95% CI) were calculated using a Wald t-distribution approximation. No correction for multiple comparisons was applied. HC: n = 71; mPFC: n = 32; LC: n = 14.

Table S6. Trial-level distributional comparisons of RT between groups.

Comparisons	KS D	KS *p* (Holm)	Energy statistic	Energy *p* (Holm)
mPFC vs HC	0.139	1.94×10^{-56}	93.17	0.0015
mPFC vs LC	0.149	6.97×10^{-31}	80.48	0.0015
HC vs LC	0.034	0.024	2.11	0.010

Note. mPFC: mPFC lesion patients, HC: Healthy controls, LC: Lesion controls. KS D values indicate the maximum difference between cumulative distribution functions in Kolmogorov–Smirnov tests. Energy statistics were obtained from permutation-based two-sample energy tests. Reported p values are Holm-adjusted for multiple comparisons. HC: n = 73; mPFC: n = 33; LC: n = 16.

Table S7. Robust linear mixed-effects model examining trial-wise changes in RT and group differences in task adaptation.

**Parameters**	**Coefficient (β)**	**SE**	**CI low**	**CI high**	***t** (df=18195)*	***p*-value**
(Intercept)	0.83	0.04	0.76	0.91	22.06	2.10×10^{-106}
Group (mPFC vs HC)	–0.12	0.05	–0.21	–0.03	–2.67	0.008
Group (mPFC vs LC)	–0.15	0.07	–0.28	–0.02	–2.26	0.024
Trial N	–0.05	0.01	–0.06	–0.04	–9.24	2.66×10^{-20}
Group (mPFC vs HC) × Trial N	-0.0007	0.006	–0.01	0.01	–0.11	0.911
Group (mPFC vs LC) × Trial N	–0.01	0.01	–0.03	0.00	–1.55	0.122

Note. mPFC: mPFC lesion patients, HC: Healthy controls, LC: Lesion controls. Trial N: Trial number. Robust linear mixed-effects models were fitted to examine trial-wise changes in reaction time and group differences in task adaptation. Coefficients are reported as fixed-effect estimates (β) with standard errors (SE) and 95% confidence intervals (CI). All hypothesis tests were two-sided. P-values and confidence intervals were calculated using a Wald t-distribution approximation. No correction for multiple comparisons was applied. HC: n = 73; mPFC: n = 33; LC: n = 16.

Table S8. Robust linear mixed-effects model comparing RTs between practice and main task phases.

Parameters	Coefficient (β)	SE	CI low	CI high	*t* (*df*=19225)	*p*-value
(Intercept)	0.84	0.04	0.77	0.92	21.82	2.82×10^{-104}
Phase (Practice vs Main)	0.88	0.02	0.83	0.92	36.61	1.05×10^{-283}
Group (mPFC vs HC)	−0.12	0.05	−0.21	−0.03	−2.65	0.008
Group (mPFC vs LC)	−0.15	0.07	−0.28	−0.02	−2.26	0.024
Phase × Group (mPFC vs HC)	−0.14	0.03	−0.20	−0.09	−5.00	5.78×10^{-7}
Phase × Group (mPFC vs LC)	−0.08	0.04	−0.16	0.01	−1.80	0.072

Note. mPFC: mPFC lesion patients, HC: Healthy controls, LC: Lesion controls. Robust linear mixed-effects models were fitted to examine reaction time between practice and the main task. Coefficients are reported as fixed-effect estimates (β) with standard errors (SE) and 95% confidence intervals (CI). All hypothesis tests were two-sided. P-values and confidence intervals were calculated using a Wald t-distribution approximation. No correction for multiple comparisons was applied. HC: $n = 73$; mPFC: $n = 33$; LC: $n = 16$.

Table S9. Robust linear mixed-effects model predicting RT with cognitive covariates.

Parameters	Coefficient (β)	SE	CI low	CI high	*t (df=17753)*	*p*-value
(Intercept)	0.74	0.08	0.69	0.90	9.43	4.55×10^{-21}
Group (mPFC vs HC)	-0.10	0.05	-0.19	-0.01	-2.24	0.025
Group (mPFC vs LC)	-0.14	0.06	-0.27	-0.02	-2.28	0.023
Ravens	-0.02	0.01	-0.04	0.00	-2.33	0.020
Trail Making Part A	0.004	0.00	0.00	0.01	2.32	0.020
Trail Making Part B	0.0008	0.00	0.00	0.00	1.26	0.209

Note. mPFC: mPFC lesion patients, HC: Healthy controls, LC: Lesion controls. Three observations were excluded due to missing data. Robust linear mixed-effects models were fitted to examine reaction time with Raven Standard Progressive Matrices and Trail Making Test Parts A and B scores as cognitive covariates. Coefficients are reported as fixed-effect estimates (β) with standard errors (SE) and 95% confidence intervals (CI). All hypothesis tests were two-sided. P-values and confidence intervals were calculated using a Wald t-distribution approximation. No correction for multiple comparisons was applied. HC: $n = 73$; mPFC: $n = 31$; LC: $n = 16$.

Table S10. Control analyses of group differences in mean payoff (ANCOVA).

Effect	df	Sum Sq	Mean Sq	F	partial η^2	p-value
Group	2	8.4	4.210	0.532	0.01	0.589
Ravens	1	5.7	5.699	0.720	0.01	0.398
Trail Making Part A	1	27.2	27.170	3.434	0.03	0.067
Trail Making Part B	1	14.3	14.270	1.803	0.02	0.182
Residual	113	894.2	7.913			

Note. Group differences in mean payoff were assessed using analysis of covariance (ANCOVA), with Raven Standard Progressive Matrices and Trail Making Test Parts A and B scores included as covariates. All hypothesis tests were two-sided. No correction for multiple comparisons was applied. Three observations were excluded due to missing data. HC: n = 73; mPFC: n = 31; LC: n = 16.

Table S11. ANCOVA results for model-derived parameters controlling cognitive measurements.

Effect	df	Sum Sq	Mean Sq	*F*	*partial η²*	*p*-value
Decision boundary						
Group	2	0.844	0.422	3.227	0.05	0.043
Ravens	1	1.057	1.057	8.074	0.07	0.005
Trail Making Part A	1	1.053	1.053	8.047	0.07	0.005
Trail Making Part B	1	0.202	0.203	1.547	0.01	0.216
Residual	113	14.787	0.131			
Drift rate temperature						
Group	2	0.002	0.001	2.630	0.04	0.077
Ravens	1	0.002	0.002	4.905	0.04	0.030
Trail Making Part A	1	0.002	0.002	4.560	0.04	0.035
Trail Making Part B	1	0.000	0.000	0.016	0.0001	0.900
Residual	113	0.052	0.000			
Non-decision time						
Group	2	0.012	0.006	0.520	0.009	0.596
Ravens	1	0.015	0.015	1.328	0.01	0.252
Trail Making Part A	1	0.015	0.014	1.397	0.01	0.240
Trail Making Part B	1	0.014	0.011	1.253	0.01	0.265
Residual	113	1.250				
Fictitious play						
Group	2	0.034	0.017	0.124	0.002	0.884
Ravens	1	0.018	0.018	0.132	0.001	0.717
Trail Making Part A	1	0.166	0.166	1.209	0.01	0.274
Trail Making Part B	1	0.576	0.576	4.198	0.04	0.043
Residual	113	15.492	0.137			
Influence play						
Group	2	0.018	0.001	0.058	0.001	0.944
Ravens	1	0.071	0.071	0.455	0.004	0.502
Trail Making Part A	1	0.059	0.059	0.375	0.003	0.542
Trail Making Part B	1	0.623	0.623	2.968	0.03	0.049
Residual	113	17.734	0.157			

Note. Group differences in decision boundary, drift rate temperature, non-decision time, fictitious play, and influence play were assessed using separate analyses of covariance (ANCOVAs), with Raven Standard Progressive Matrices and Trail Making Test Parts A and B scores included as covariates. All hypothesis tests were two-sided. No correction for multiple comparisons was applied. Three observations were excluded due to missing data. HC: $n = 73$; mPFC: $n = 31$; LC: $n = 16$.

Table S12. Linear mixed-effects model predicting payoff from decision boundary α.

**Fixed Effects**	**Coefficient (β)**	**SE**	**95% CI**	**df**	***t***	***p*-value**
(Intercept)	26.04	0.20	[25.65, 26.43]	132.16	131.26	7.16×10^{-142}
Medium α vs Low α	-0.32	0.07	[-0.46, -0.18]	242	-4.47	1.23×10^{-5}
Medium α vs High α	0.22	0.07	[0.08, 0.36]	242	3.08	0.002

Note. Payoff values obtained from model simulations in which the decision boundary parameter (α) was systematically varied across three levels (low, medium [fitted value], high), while all other parameters were held constant at their fitted values. Estimates are reported relative to the medium α condition. Effects were analysed using a two-sided linear mixed-effects model with participant as a random intercept. Confidence intervals were calculated using the Wald method. $N = 122$.

Table S13. Summary of behavioural results, excluding 3 TPJ lesion patients in the LC group

**Variable**	**HC (*n* = 73)** mean [SD]	**mPFC (*n* = 33)** mean [SD]	**LC-E (*n* = 13)** mean [SD]	**Statistics**	**HC vs mPFC** *p*-value (BF_{01})	**HC vs LC-E** *p*-value (BF_{01})	**LC-E vs mPFC** *p*-value (BF_{01})
Age	60.53 [18.69]	56.88 [10.71]	56.77 [11.48]	$F_{(2,116)}$=0.73, p=0.483, η^2=0.013	0.531 (BF_{01} = 2.81)	0.721 (BF_{01} = 2.55)	1.000 (BF_{01} = 3.26)
Gender	47.9% M	48.5% M	30.8% M	$\chi^2_{(2)}$=1.40, p=0.496, Cramer's V=0.109	1.000 (BF_{01} = 3.90)	0.399 (BF_{01} = 1.52)	0.447 (BF_{01} = 1.51)
Payoff (credits)	25.67 [2.96]	25.21 [2.70]	25.45 [2.72]	$F_{(2,116)}$=0.29, p=0.75, η^2=0.005	0.731 (BF_{01} = 3.47)	0.965 (BF_{01} = 3.23)	0.966 (BF_{01} = 3.10)
RT (s)	0.80 [0.27]	1.03 [0.48]	0.80 [0.26]	$F_{(2,116)}$=5.47, p=0.005, η^2=0.086	0.005 (BF_{01} = 0.12)	1.000 (BF_{01} = 3.37)	0.097 (BF_{01} = 1.13)

Note. HC: Healthy controls, LC-E: Lesion controls without TPJ lesion patients (*N*=13), SD: standard deviation, BF_{01}: Bayes factors of the null hypothesis. M: male. Group differences in continuous variables were assessed using one-way analysis of variance (ANOVA) followed by Tukey's honestly significant difference (HSD) post hoc tests with adjustment for multiple comparisons. Gender distributions were compared using Pearson's chi-square tests; pairwise comparisons were further corrected using Holm's method for multiple comparisons (all adjusted p-values = 1.000). All statistical tests were two-sided.

Table S14. Summary of behavioural results, excluding unilateral mPFC lesion patients

Variable	**HC (*n* = 73)** mean [SD]	**mPFC-B (*n* = 17)** mean [SD]	**LC-E (*n* = 16)** mean [SD]	**Statistics**	**HC vs mPFC-B** *p*-value (BF_{01})	**HC vs LC** *p*-value (BF_{01})	**LC vs mPFC-B** *p*-value (BF_{01})
Age	60.53 [18.69]	58.06 [11.14]	56.06 [11.43]	$F_{(2,103)}$=0.54, p=0.587, η^2=0.010	0.848 (BF_{01} = 2.81)	0.601 (BF_{01} = 2.56)	0.938 (BF_{01} = 3.26)
Gender	47.9% M	52.9% M	31.3% M	$\chi^2_{(2)}$=1.84, p=0.399, Cramer's V=0.132	0.919 (BF_{01} = 2.90)	0.348 (BF_{01} = 1.52)	0.364 (BF_{01} = 1.17)
Payoff (credits)	25.67 [2.96]	25.15 [2.82]	25.54 [2.80]	$F_{(2,103)}$=0.22, p=0.807, η^2=0.004	0.790 (BF_{01} = 2.99)	0.985 (BF_{01} = 3.53)	0.926 (BF_{01} = 2.84)
RT (s)	0.80 [0.27]	1.08 [0.57]	0.77 [0.24]	$F_{(2,103)}$=5.31, p=0.006, η^2=0.093	0.006 (BF_{01} = 0.10)	0.962 (BF_{01} = 3.56)	0.026 (BF_{01} = 0.85)

Note. HC: Healthy controls, mPFC-B: Bilateral mPFC lesion patients (*n* = 17), LC: Lesion controls, SD: standard deviation, BF_{01}: Bayes factors of the null hypothesis. M: male. Group differences in continuous variables were assessed using one-way analysis of variance (ANOVA) followed by Tukey's honestly significant difference (HSD) post hoc tests with adjustment for multiple comparisons. Gender distributions were compared using Pearson's chi-square tests; pairwise comparisons were further corrected using Holm's method for multiple comparisons (all adjusted p-values = 1.000). All statistical tests were two-sided.

Table S15. Robust linear mixed-effects model predicting RT from continuous second-order belief updates magnitude

**Parameters**	**Coefficient (β)**	**SE**	**CI low**	**CI high**	***t*** *(df=18113)*	***p*-value**
(Intercept)	0.83	0.04	0.76	0.91	22.21	7.18×10^{-108}
Group (mPFC vs HC)	–0.12	0.05	–0.21	–0.03	–2.68	0.007
Group (mPFC vs LC)	–0.15	0.07	–0.28	–0.02	–2.28	0.023
Belief updates	–0.02	0.01	–0.03	–0.01	–4.30	1.74×10^{-5}
Group (mPFC vs HC) × Belief updates	–0.02	0.01	0.00	0.03	2.63	0.009
Group (mPFC vs LC) × Belief updates	–0.01	0.01	–0.01	0.02	0.71	0.480

Note. mPFC: mPFC lesion patients, HC: Healthy controls, LC: Lesion controls. Reaction time (RT) was modelled using a two-sided robust linear mixed-effects model with participant as a random intercept. The model included group, continuous second-order belief update magnitude, and their interaction as fixed effects. Coefficients represent fixed-effect estimates (β). P-values and confidence intervals (95% CI) were calculated using a Wald t-distribution approximation. No correction for multiple comparisons was applied. HC: *n* = 73; mPFC: *n* = 33; LC: *n* = 16.

Table S16. Sensitivity analyses for primary Bayesian ANOVAs across alternative prior widths

**Analysis (BF_{01})**	**Narrow (0.25)**	**Medium (0.5)**	**Wide (1)**	**Conclusion**
Payoff	3.36	7.98	25.10	Support for H_0 across priors
Fictitious Play	3.56	8.62	27.39	Support for H_0 across priors
Influence Play	3.78	9.24	29.43	Support for H_0 across priors

Note. $BF_{01} > 1$ indicates evidence in favour of the null hypothesis. Sensitivity analyses were conducted using alternative prior widths (rscale = 0.25, 0.5, and 1). Conclusions were considered robust when the direction of evidence remained unchanged across prior specifications.

Table S17. One-sample tests comparing subjective strategy ratings against the midpoint value.

Strategy	mean	$t_{(df)}$	P value	d	CI	BF_{10}	BF_{01}
HC							
Play past	5.60	$t_{(72)}$=2.45	0.008	0.287	[5.11, 6.09]	4.15	0.24
Reactive	6.25	$t_{(72)}$=5.22	8.25E-07	0.611	[5.77, 6.72]	1.88E+04	5.32E-05
Influence	6.86	$t_{(72)}$=6.00	3.61E-08	0.702	[6.24, 7.48]	3.61E+05	2.77E-06
Tracking	6.90	$t_{(72)}$=8.19	3.44E-12	0.958	[6.44, 7.37]	2.55E+09	3.92E-10
Adaptive	6.50	$t_{(15)}$=2.22	0.021	0.554	[5.06, 7.94]	3.31	0.30
mPFC							
Play past	5.84	$t_{(31)}$=2.26	0.015	0.400	[5.08, 6.61]	3.37	0.30
Reactive	6.50	$t_{(31)}$=4.24	9.27E-05	0.750	[5.78, 7.22]	2.93E+02	3.41E-03
Influence	6.06	$t_{(31)}$=2.66	0.006	0.471	[5.25, 6.88]	7.41	0.13
Tracking	7.19	$t_{(31)}$=5.57	2.08E-06	0.985	[6.39, 7.99]	9.37E+03	1.07E-04
Adaptive	7.28	$t_{(31)}$=6.20	3.52E-07	1.095	[6.53, 8.03]	4.86E+04	2.06E-05
LC							
Play past	4.81	$t_{(15)}$=-0.26	0.6	-0.065	[3.27, 6.36]	0.21	4.69
Reactive	5.56	$t_{(15)}$=0.94	0.181	0.235	[4.29, 6.84]	0.60	1.66
Influence	5.56	$t_{(15)}$=1.03	0.16	0.257	[4.40, 6.73]	0.66	1.50
Tracking	6.88	$t_{(15)}$=2.88	0.006	0.720	[5.49, 8.26]	9.70	0.10
Adaptive	6.50	$t_{(15)}$=2.22	0.021	0.554	[5.06, 7.94]	3.31	0.30

Note. Strategies were assessed using the following statements: Play past (I played an option that worked in the past), Reactive (I felt my opponent was reacting to how I was playing), Influence (I tried to influence my opponent's choice), Tracking (I tried to keep track of how my opponent played), and Adaptive (I tried to adjust my behaviour based on the opponent's choices).. Subjective strategy ratings were compared against the midpoint value of 5 using one-sided one-sample t-tests (alternative hypothesis: mean > 5). Mean values represent participants' self-reported strategy use ratings on a 1–10 scale (1 = never, 10 = always). The t statistic, degrees of freedom (df), and p values are reported from the one-sided tests. Cohen's d was calculated as the standardised mean difference from the midpoint value. Confidence intervals (95% CI) are two-sided confidence intervals for the estimated mean. Bayesian evidence was quantified using one-sided Bayesian one-sample t-tests, with BF_{10} representing evidence for the alternative hypothesis and BF_{01} representing evidence for the null hypothesis. HC: Healthy controls (*n* = 73), mPFC: mPFC lesion patients(*n* = 32), LC: Lesion controls (*n* = 16).

Table S18. Group differences in self-reported strategy use during the inspection game.

Strategy	$F_{(df1, df2)}$	p value	η^2	BF_{10}	BF_{01}
Play past	$F_{(2,118)}$=1.18	0.311	0.020	0.23	4.38
Reactive	$F_{(2,118)}$=1.1	0.337	0.018	0.22	4.65
Influence	$F_{(2,118)}$=2.38	0.097	0.039	0.68	1.48
Tracking	$F_{(2,118)}$=0.22	0.806	0.004	0.16	8.65
Adaptive	$F_{(2,118)}$=0.73	0.483	0.012	0.17	6.01

Note. One-way analyses of variance (ANOVAs) were conducted to examine group differences in participants' self-reported strategy use during the inspection game. Participants rated the extent to which they used each strategy on a 10-point scale (1 = never, 10 = always). ANOVAs were conducted using a two-sided test. Test statistics are reported as F-statistics with corresponding degrees of freedom. Effect sizes are reported as eta squared (η^2). Bayesian ANOVA results are reported as BF_{10} (evidence for the alternative hypothesis) and BF_{01} (evidence for the null hypothesis). BF_{01} was calculated as the reciprocal of BF_{10}. Strategies were assessed using the following statements: Play past (I played an option that worked in the past), Reactive (I felt my opponent was reacting to how I was playing), Influence (I tried to influence my opponent's choice), Tracking (I tried to keep track of how my opponent played), and Adaptive (I tried to adjust my behaviour based on the opponent's choices). HC: Healthy controls (*n* = 73), mPFC: mPFC lesion patients(*n* = 32), LC: Lesion controls (*n* = 16).

Table S19. Pairwise Bayesian comparisons of self-reported strategy use.

Strategy	Comparisons	BF_{10}	BF_{01}
Play past	HC vs mPFC	0.25	3.96
	mPFC vs LC	0.66	1.52
	HC vs LC	0.54	1.87
Reactive	HC vs mPFC	0.26	3.87
	mPFC vs LC	0.68	1.47
	HC vs LC	0.49	2.04
Influence	HC vs mPFC	0.58	1.71
	mPFC vs LC	0.37	2.68
	HC vs LC	1.09	0.92
Tracking	HC vs mPFC	0.27	3.75
	mPFC vs LC	0.32	3.08
	HC vs LC	0.28	3.60
Adaptive	HC vs mPFC	0.28	3.52
	mPFC vs LC	0.49	2.04
	HC vs LC	0.36	2.82

Note. Pairwise Bayesian independent-samples t-tests were conducted between participant groups. BF_{10} represents evidence for group differences, whereas BF_{01} represents evidence for the null hypothesis. BF_{01} values were calculated as the reciprocal of BF_{10}. Strategies were assessed using the following statements: Play past (I played an option that worked in the past), Reactive (I felt my opponent was reacting to how I was playing), Influence (I tried to influence my opponent's choice), Tracking (I tried to keep track of how my opponent played), and Adaptive (I tried to adjust my behaviour based on the opponent's choices). HC: Healthy controls (n = 73), mPFC: mPFC lesion patients(n = 32), LC: Lesion controls (n = 16).

Supplement References

1. Crockett, M. J. *et al.* Dissociable Effects of Serotonin and Dopamine on the Valuation of Harm in Moral Decision Making. *Curr. Biol.* **25**, 1852–1859 (2015).
2. Lockwood, P. L. *et al.* Aging Increases Prosocial Motivation for Effort. *Psychol. Sci.* **32**, 668–681 (2021).
3. Lockwood, P. L. *et al.* Distinct neural representations for prosocial and self-benefiting effort. *Curr. Biol.* **32**, 4172-4185.e7 (2022).
4. Cutler, J. *et al.* Ageing is associated with disrupted reinforcement learning whilst learning to help others is preserved. *Nat. Commun.* **12**, 4440 (2021).
5. Ang, Y.-S., Lockwood, P., Apps, M. A. J., Muhammed, K. & Husain, M. Distinct Subtypes of Apathy Revealed by the Apathy Motivation Index. *PLoS ONE* **12**, e0169938 (2017).
6. BECK, A. T., WARD, C. H., MENDELSON, M., MOCK, J. & ERBAUGH, J. An Inventory for Measuring Depression. *Arch. Gen. Psychiatry* **4**, 561–571 (1961).
7. Reitan, R. M. The relation of the Trail Making Test to organic brain damage. *J. Consult. Psychol.* **19**, 393–394 (1955).
8. RAVEN, J. C. Mental tests used in genetic,The performance of related indiviuals on tests mainly educative and mainly reproductive. *MSC ThesisUniv Lond.* https://cir.nii.ac.jp/crid/1572824499124445056 (1936).
9. Hsieh, S., Schubert, S., Hoon, C., Mioshi, E. & Hodges, J. R. Validation of the Addenbrooke's Cognitive Examination III in Frontotemporal Dementia and Alzheimer's Disease. *Dement. Geriatr. Cogn. Disord.* **36**, 242–250 (2013).
10. R Core Team. R: A Language and Environment for Statistical Computing. R Foundation for Statistical Computing, Vienna, Austria (2024).

11. Posit team. RStudio: Integrated Development Environment for R. Posit Software. PBC, Boston, MA. (2024).
12. Wickham, H. *Ggplot2: Elegant Graphics for Data Analysis*. (Springer International Publishing, New York, 2016).
13. CRAN: Package dplyr. https://cran.r-project.org/web/packages/dplyr/index.html.
14. Wickham, H. *et al.* Welcome to the Tidyverse. *J. Open Source Softw.* **4**, 1686 (2019).
15. Morey, R. D. *et al.* BayesFactor: Computation of Bayes Factors for Common Designs. (2024).
16. Koller, M. robustlmm: An R Package for Robust Estimation of Linear Mixed-Effects Models. *J. Stat. Softw.* **75**, 1–24 (2016).
17. Lenth, R. V. *et al.* emmeans: Estimated Marginal Means, aka Least-Squares Means. (2025).
18. Daniel, F., Corporation, M., Weston, S. & Tenenbaum, D. doParallel: Foreach Parallel Adaptor for the 'parallel' Package. (2022).
19. Daniel, F., Ooi, H., Calaway, R., Microsoft & Weston, S. foreach: Provides Foreach Looping Construct. (2022).
20. Wabersich, D. & Vandekerckhove, J. The RWiener Package: an R Package Providing Distribution Functions for the Wiener Diffusion Model. *R J.* **6**, 49 (2014).
21. Wilson, R. C. & Collins, A. G. Ten simple rules for the computational modeling of behavioral data. *eLife* **8**, e49547 (2019).
22. Smith, S. M. *et al.* Advances in functional and structural MR image analysis and implementation as FSL. *NeuroImage* **23**, S208–S219 (2004).
23. Baldo, J. V. *et al.* Voxel-based lesion analysis of brain regions underlying reading and writing. *Neuropsychologia* **115**, 51–59 (2018).

24. Smith, S. M. & Nichols, T. E. Threshold-free cluster enhancement: Addressing problems of smoothing, threshold dependence and localisation in cluster inference. *NeuroImage* **44**, 83–98 (2009).

25. Mirman, D. *et al.* Corrections for multiple comparisons in voxel-based lesion-symptom mapping. *Neuropsychologia* **115**, 112–123 (2018).

26. Mai, J. K., Majtanik, M. & Paxinos, G. *Atlas of the Human Brain*. (Academic Press, 2015).

27. Marozzi, M. Nonparametric Simultaneous Tests for Location and Scale Testing: A Comparison of Several Methods. *Commun. Stat. - Simul. Comput.* **42**, 1298–1317 (2013).

28. Székely, G. J. & Rizzo, M. L. Energy statistics: A class of statistics based on distances. *J. Stat. Plan. Inference* **143**, 1249–1272 (2013).

29. Hampton, A. N., Bossaerts, P. & O'Doherty, J. P. Neural correlates of mentalizing-related computations during strategic interactions in humans. *Proc. Natl. Acad. Sci.* **105**, 6741–6746 (2008).
