# Supplementary material for: Preserved higher-order mentalising during strategic interactions after medial prefrontal cortex damage
Source: Nat Commun. 2026 Oct 10;17:11514. doi: 10.1038/s41467-026-77235-3 (PMC13745589; doi:10.1038/s41467-026-77235-3)
Supplement: Supplementary file 2 [file 41467_2026_77235_MOESM2_ESM.pdf]

nature portfolio

Corresponding author(s): Yi-Chen Hsieh

Last updated by author(s): Jul 9, 2026

Reporting Summary

Nature Portfolio wishes to improve the reproducibility of the work that we publish. This form provides structure for consistency and transparency in reporting. For further information on Nature Portfolio policies, see our Editorial Policies and the Editorial Policy Checklist.

Statistics

For all statistical analyses, confirm that the following items are present in the figure legend, table legend, main text, or Methods section.

n/a	Confirmed	
☐	☒	The exact sample size (*n*) for each experimental group/condition, given as a discrete number and unit of measurement
☐	☒	A statement on whether measurements were taken from distinct samples or whether the same sample was measured repeatedly
☐	☒	The statistical test(s) used AND whether they are one- or two-sided<br>*Only common tests should be described solely by name; describe more complex techniques in the Methods section.*
☐	☒	A description of all covariates tested
☐	☒	A description of any assumptions or corrections, such as tests of normality and adjustment for multiple comparisons
☐	☒	A full description of the statistical parameters including central tendency (e.g. means) or other basic estimates (e.g. regression coefficient) AND variation (e.g. standard deviation) or associated estimates of uncertainty (e.g. confidence intervals)
☐	☒	For null hypothesis testing, the test statistic (e.g. *F*, *t*, *r*) with confidence intervals, effect sizes, degrees of freedom and *P* value noted<br>*Give P values as exact values whenever suitable.*
☐	☒	For Bayesian analysis, information on the choice of priors and Markov chain Monte Carlo settings
☐	☒	For hierarchical and complex designs, identification of the appropriate level for tests and full reporting of outcomes
☐	☒	Estimates of effect sizes (e.g. Cohen's *d*, Pearson's *r*), indicating how they were calculated

Our web collection on statistics for biologists contains articles on many of the points above.

Software and code

Policy information about availability of computer code

Data collection	We used MATLAB 2012a (MathWorks) together with the Cogent 2000 v125 toolbox (www.vislab.ucl.ac.uk/Cogent/) to present the task stimuli and record behavioural responses.
Data analysis	Analyses were conducted in R (v4.3.1) using RStudio v2024.09.1+394 and Positron (2026.02.1). Packages included dplyr, lme4, emmeans, tidyverse, robustlmm, BayesFactor, RWiener, doParallel, foreach, stats, and ggplot2. Custom R scripts were developed for data preprocessing, model fitting, and plotting. Matlab was used to create lesion mapping used files. MRIcroGL (Version 15.7.7) was used to visualize the brain images.

For manuscripts utilizing custom algorithms or software that are central to the research but not yet described in published literature, software must be made available to editors and reviewers. We strongly encourage code deposition in a community repository (e.g. GitHub). See the Nature Portfolio guidelines for submitting code & software for further information.

Data

Policy information about availability of data

All manuscripts must include a data availability statement. This statement should provide the following information, where applicable:

- Accession codes, unique identifiers, or web links for publicly available datasets
- A description of any restrictions on data availability
- For clinical datasets or third party data, please ensure that the statement adheres to our policy

Data Availability
The behavioural, questionnaire, computational modelling, and lesion-symptom mapping data generated in this study have been deposited in the Open Science Framework (OSF) repository under accession code https://doi.org/10.17605/OSF.IO/AX85S.
The processed behavioural and neuropsychological data used for all analyses are publicly available in the OSF repository. Lesion-symptom mapping input files and permutation-based statistical outputs are also provided to enable full reproducibility of the reported analyses.
Raw neuroimaging and clinical data are not publicly available due to participant privacy and ethical restrictions under institutional data protection policies. All data required to reproduce the results reported in this study are available in the OSF repository under the same accession code.

Code Availability
All analysis code used in this study has been deposited in the Open Science Framework (OSF) repository under accession code https://doi.org/10.17605/OSF.IO/AX85S

Research involving human participants, their data, or biological material

Policy information about studies with human participants or human data. See also policy information about sex, gender (identity/presentation), and sexual orientation and race, ethnicity and racism.

Reporting on sex and gender	Biological sex (male/female) was recorded based on self-report. The groups (mPFC lesion: n = 33, 17 females; lesion control: n = 16, 16 females; healthy control, n = 73, 38 females) were balanced for sex, with no significant differences in the number of male and female participants between groups. Sex was not a focus of the study and analyses were not stratified by sex.
Reporting on race, ethnicity, or other socially relevant groupings	No race or ethnicity variables were collected, as they were not relevant to the study aims.
Population characteristics	Participants included patients with focal mPFC lesions (aetiologies: subarachnoid haemorrhage due to ruptured aneurysms, frontal meningioma resection, and ischaemic stroke), lesion controls with non-mPFC focal lesions, and healthy controls matched on age, sex, and education. Basic demographic variables and clinical details are reported in the manuscript.
Recruitment	Participants were recruited from a database of 453 neurological patients, university subject databases, and the community.
Ethics oversight	All participants provided written consent. The study was ethically approved by the Oxford University Medical Sciences Inter Divisional Research Ethics Committee and National Health Service Ethics.

Note that full information on the approval of the study protocol must also be provided in the manuscript.

Field-specific reporting

Please select the one below that is the best fit for your research. If you are not sure, read the appropriate sections before making your selection.

☐ Life sciences ☒ Behavioural & social sciences ☐ Ecological, evolutionary & environmental sciences

For a reference copy of the document with all sections, see nature.com/documents/nr-reporting-summary-flat.pdf

Behavioural & social sciences study design

All studies must disclose on these points even when the disclosure is negative.

Study description	Quantitative experimental study with between-group comparisons (lesion vs. lesion control vs. healthy control), using a repeated-trial social decision-making task (Inspection Game).
Research sample	The final sample consisted of 33 mPFC lesion patients, 16 lesion controls, and 73 healthy controls. Groups were matched on age, sex, and education.
Sampling strategy	The sample size was determined by the availability of suitable patients in our clinical recruitment pool. All patients with focal mPFC or non-mPFC lesions who met the inclusion criteria during the recruitment period were invited to participate. The final sample is relatively large for lesion studies, particularly the mPFC group, and is consistent with sample sizes reported in previous cognitive neuroscience lesion research. This sample is therefore sufficient to address the study aims.

Data collection	Data were collected using a computer-based task programmed in MATLAB 2012a (MathWorks) with the Cogent 2000 v125 toolbox (www.vislab.ucl.ac.uk/Cogent/). Participants performed a repeated-trial social decision-making task in which they believed they were interacting with another player.
Timing	Data were collected between May 2017 and October 2019. All patients were tested at least 24 months post-lesion.
Data exclusions	Participants with incomplete task data, excessive missing trials (>20%), or technical errors during data collection were set as exclusion criteria. However, no participants met these criteria and no data were removed on this basis. One participant did not complete the post-task strategy questionnaire and was therefore excluded from the corresponding analyses only.
Non-participation	No participants dropped out after consenting to participate.
Randomization	Participants were assigned to groups based on lesion location or healthy volunteer status; randomization was not applicable.

Reporting for specific materials, systems and methods

We require information from authors about some types of materials, experimental systems and methods used in many studies. Here, indicate whether each material, system or method listed is relevant to your study. If you are not sure if a list item applies to your research, read the appropriate section before selecting a response.

Materials & experimental systems

n/a	Involved in the study
☒	☐ Antibodies
☒	☐ Eukaryotic cell lines
☒	☐ Palaeontology and archaeology
☒	☐ Animals and other organisms
☒	☐ Clinical data
☒	☐ Dual use research of concern
☒	☐ Plants

Methods

n/a	Involved in the study
☒	☐ ChIP-seq
☒	☐ Flow cytometry
☐	☒ MRI-based neuroimaging

Plants

Seed stocks	*Report on the source of all seed stocks or other plant material used. If applicable, state the seed stock centre and catalogue number. If plant specimens were collected from the field, describe the collection location, date and sampling procedures.*
Novel plant genotypes	*Describe the methods by which all novel plant genotypes were produced. This includes those generated by transgenic approaches, gene editing, chemical/radiation-based mutagenesis and hybridization. For transgenic lines, describe the transformation method, the number of independent lines analyzed and the generation upon which experiments were performed. For gene-edited lines, describe the editor used, the endogenous sequence targeted for editing, the targeting guide RNA sequence (if applicable) and how the editor was applied.*
Authentication	*Describe any authentication procedures for each seed stock used or novel genotype generated. Describe any experiments used to assess the effect of a mutation and, where applicable, how potential secondary effects (e.g. second site T-DNA insertions, mosiacism, off-target gene editing) were examined.*

Magnetic resonance imaging

Experimental design

Design type	Structural MRI for lesion identification and mapping.
Design specifications	Not applicable (no task-based MRI; structural scans only for lesion delineation).
Behavioral performance measures	Not applicable (behavioural data were collected separately outside of MRI).

Acquisition

Imaging type(s)	Structural MRI; CT (for 2 patients with MRI contraindications)
Field strength	3 Tesla.
Sequence & imaging parameters	1 mm isotropic T1-weighted FSPGR MRI; 6 mm axial T2 PROPELLER sequence.
Area of acquisition	Whole brain.

Diffusion MRI ☐ Used ☒ Not used

Preprocessing

Preprocessing software	FSL v5.5 (http://fsl.fmrib.ox.ac.uk/fsl). Lesions were manually traced by a neurologist on structural scans.
Normalization	Structural scans normalized to MNI152 space.
Normalization template	MNI152 template.
Noise and artifact removal	Not applicable (structural MRI only).
Volume censoring	Not applicable.

Statistical modeling & inference

Model type and settings	Voxel-based lesion–symptom mapping (VLSM) analysis; lesion overlap maps were generated to visualise the spatial distribution of lesions.
Effect(s) tested	Association between lesion location and behavioural task model fitting parameters.

Specify type of analysis: ☐ Whole brain ☐ ROI-based ☒ Both

Anatomical location(s): Significant clusters were identified using binarised t-value masks and labelled with BioImage Suite (https://www.bioimagesuite.org) and the Atlas of the Human Brain, 4th edition, in MNI space.

Statistic type for inference (See Eklund et al. 2016)	Nonparametric permutation testing with threshold-free cluster enhancement (TFCE; 5000 permutations, default settings).
Correction	Bonferroni correction for multiple comparisons: $p < 0.01$ for all computational model-derived parameters.

Models & analysis

n/a	Involved in the study
☒	☐ Functional and/or effective connectivity
☒	☐ Graph analysis
☒	☐ Multivariate modeling or predictive analysis
