# Supplementary material for: Preserved higher-order mentalising during strategic interactions after medial prefrontal cortex damage
Source: Nat Commun. 2026 Oct 10;17:11514. doi: 10.1038/s41467-026-77235-3 (PMC13745589; doi:10.1038/s41467-026-77235-3)
Supplement: Supplementary file 3 [file 41467_2026_77235_MOESM3_ESM.pdf]

nature portfolio

Peer Review File

Preserved Higher-order Mentalising During Strategic Interactions After Medial Prefrontal Cortex Damage

Corresponding Author: Ms Yi-Chen Hsieh

This file contains all reviewer reports in order by version, followed by all author rebuttals in order by version.

Version 0:

Reviewer comments:

Reviewer #1

(Remarks to the Author)
Hsieh et al. use an established social interaction task to test if patients with lesions in MPFC regions or in control regions show altered social decision-making. Overall, their results do not support altered choice behaviour (using standard statistical methods and computational models). But RTs in the group with MPFC lesions tend to be longer, which is mirrored in the decision boundary parameter in the winning drift diffusion model variant.
In my view, the article addresses an important, timely question with well-suited experimental and analytical procedures. Please find my suggestions for improvement below. Sometimes crucial information seems to be buried in the supplementary materials.
1. RTs: Given that the group difference relies largely on RTs, it would be important to see a few more details:
a. Could you show some plots with the distribution of RTs per participant and describe these in general? It should be shown that the effect is not driven by some extreme values (i.e., neither extreme individuals nor extreme trials).
b. Did you exclude any extremely short or long RTs?
c. Often RTs are log-transformed. Are there any specific reasons for not doing this here?
d. In general, it would be good to use LMEs here. If I understood correctly from the supplementary information, you did this for analysing the effects of 2nd order beliefs on RTs. Why not for the main analyses?
e. Did the RTs become faster over the course of the experiments? This is often the case. Could it be that the group with MPFC lesions did not "adapt" to the task as swiftly as the controls?
f. Relatedly: Did the groups differ in the time they needed to understand the task? This time for the instruction is usually not measured but maybe some participants had to repeat the training session and some might have much longer RTs in the training sessions.
g. On page 13 you write: "Our behavioural analysis showed that mPFC damage was in general associated with preserved mentalising but slower decision-making, due to higher boundaries for the evidence accumulation process during strategic interactions." This causal language is not warranted at this point in the manuscript.
h. Related to 1d: Why did you use a median split? I can understand that this is easier to plot (cf. Fig. S5). Do the results hold when using the full range of (absolute) updates?
i. I find Fig. S3A & 3B quite informative (both for RTs and for choices). However, here it is unclear which data points are plotted exactly. It could be helpful to make these plots separately for the three groups.
2. Control questionnaires and tasks:
a. Did participants perform some sort of non-social control task? If so, did you look into RTs?
b. You write: "After the series of tasks was done (three other experimental tasks were reported elsewhere), participants completed a range of questionnaires (see below and Supplementary Experimental Procedures)" Which ones? You don't give any citations for the "reported elsewhere."
c. You write: "Fluid intelligence was measured using a shortened version of the Raven Standard Progressive Matrices (RSPM) 119. The Addenbrooke's Cognitive Examination (ACE-III) was used to assess global cognitive functioning across attention, memory, fluency, language, and visuospatial domains in participants over 40 and in both lesion groups 120 (see Supplementary Experimental Procedures for the full descriptions of the assessments)." However, the groups differed in these! You don't mention this anywhere, which seems like hiding a crucial difference! In contrast, the absence of differences for other tests is explicitly mentioned: "In addition, the mPFC patients did not differ significantly from the healthy or lesion control groups in the neuropsychological test assessing cognitive ability and visual attention (Trail Making Test; Part A ps > 0.62, Part B ps > 0.12)."
3. Modelling:

a. The DDM could be explained a bit more in the main text; especially the role of the RTs in the DDM
b. In the supplementary materials it becomes clear that the "Influence-DDM best fits the mPFC and HC groups, while the LC group favours simpler fictitious-DDM." This has to be explicitly mentioned in the main text and should be carefully discussed.
c. Drift rate temperature and non-decision time did not differ between the groups. It should be clearer why it is still warranted to perform VSLM.
d. Relatedly: How correlated are they with each other and with the decision boundary? That is, how independent are the analyses showing the considerably overlapping regions in Fig. 5?
e. Do simulations with different decision boundaries show that the payoffs remain the same?
4. Discussion / introduction: It would be good to discuss the current results a bit more with reference to …
a. … other lesion studies that address overall cognitive deficits (or their absence)
b. … possible compensation "mechanisms" via the short- or long-term recruitment of other brain regions (possibly via plastic changes after the lesion).
5. Minor: I find it helpful to link all supplementary figures to the relevant main figures and vice versa.

(Remarks on code availability)

Reviewer #2

(Remarks to the Author)
This article presents a novel and sophisticated study with valuable insights into mentalizing capacities in patients with lesions in the medial prefrontal cortex (mPFC). The authors have performed and described the lesion location as well as behavioral modeling in a very comprehensive manner, which is a significant strength of the manuscript. The core finding—that mPFC damage impacts the speed rather than the capacity for mentalizing during social interactions—offers a nuanced understanding of the mPFC's role in social cognition and is a critical contribution to the field. The use of a computational model combining learning and drift-diffusion processes to dissociate various underlying mechanisms is particularly commendable, as is the consistency between behavioral findings and self-reports of mentalizing strategy use in mPFC patients. This methodological rigor, employing state-of-the-art computational modeling and voxel-based lesion-symptom mapping (VLSM) on a valuable cohort of patients with focal brain lesions, represents a significant advancement.

However, despite these strengths, several aspects of the manuscript could be clarified or strengthened. The introduction, for instance, dedicates a significant portion to summarizing the study, which feels repetitive after reading the abstract. This space could be more effectively utilized to provide a more comprehensive theoretical background on the general consequences of focal brain lesions, or to offer a deeper justification for the specific choices made regarding lesion and control groups in the context of prior mentalizing research.

A central point of concern revolves around the sample size and how it is presented. While the mPFC lesion sample of N=33 is indeed remarkably large for a patient study, the manuscript frequently describes it as "large" and combines it with the "other lesion" control group (N=16) to form an even larger sample (33+16) in certain analyses. The primary value of this paper lies in the homogeneity and focal nature of the mPFC lesion sample, and combining it with a heterogeneous "other lesion" group in analyses where precise lesion localization is paramount risks diluting the findings. This practice appears to undermine the very advantage gained by focusing on specific focal lesions and may artificially inflate statistical power rather than genuinely enhancing the reliability or specificity of the results. This issue becomes particularly salient for the subregion analyses (VLSM). To truly identify mPFC subregions causally implicated in the observed effects, this analysis should ideally be restricted solely to the focal mPFC lesion group. Including the "other lesion" sample, which by definition should not have mPFC lesions, cannot logically contribute to finding specific mPFC subregions and merely serves to increase the statistical N, potentially masking true regional effects within the mPFC itself.

Furthermore, the choice and characterization of the lesion control group (N=16) are somewhat unclear. This group is rather small and appears to have a heterogeneous lesion profile, concentrated predominantly in right-lateralized temporal cortex regions as suggested by Figure 1B. It is challenging to argue that this group effectively serves as a general control for the "presence of a lesion" without risking confounding factors related to the specific brain regions affected. This raises questions about its added value over the healthy control group, particularly in distinguishing effects specific to mPFC damage from more general lesion-related deficits or from effects related to specific non-mPFC lesion sites. A more detailed justification for its specific utility as a control for mPFC lesions is needed.

Regarding the assessment of "conserved cognitive abilities," the manuscript primarily mentions the Trail Making Test Part A (TMT-A) as a control measure. While TMT-A assesses visual attention, it is not a comprehensive measure of general cognitive abilities and does not cover crucial domains such as memory, logical reasoning, or language. For a study focusing on mentalizing, having a broader battery of non-social cognitive reasoning measures would be vital to ensure that any observed preserved abilities or deficits are indeed socially specific and not simply attributable to more general cognitive impairments or preservation. The claims of preserved performance would be substantially strengthened if other cognitive domains were explicitly controlled for.

Finally, concerns arise about the ecological validity of the experimental paradigm. The description of the "presentation" of the confederate as an anonymous, physically met "other player" who is actually an algorithm feels highly artificial and unnatural. This approach, while controlled, might differ significantly from more ecologically valid social interactions, such as video-mediated interactions. Moreover, despite its computational sophistication, the paradigm, in its simplicity, might be more accurately conceptualized as a prediction error paradigm or strategic learning task rather than a nuanced

representation of complex "mentalizing" in its broader sense. Clarifying the precise aspects of mentalizing that the paradigm is intended to capture would be beneficial for interpreting the "preserved mentalizing" claims.

Despite these criticisms, this reviewer believes the study presents important new data and innovative analytical approaches to a challenging research question. Addressing the points raised, particularly by refining the control group comparisons, clarifying the precise scope of "mentalizing" within the chosen paradigm, and strengthening the cognitive control measures, would significantly enhance the manuscript's conclusions and overall impact for Nature Communications.

(Remarks on code availability)

Reviewer #3

(Remarks to the Author)
I co-reviewed this manuscript with one of the reviewers who provided the listed reports. This is part of the Nature Communications initiative to facilitate training in peer review and to provide appropriate recognition for Early Career Researchers who co-review manuscripts.

(Remarks on code availability)

Reviewer #4

(Remarks to the Author)
Review of NCOMMS-25-61816

In the manuscript under review, Hsieh et al. examined whether strategic mentalization was altered or intact in a relatively large sample of patients with mPFC damage. Previous work in healthy adults suggests that the mPFC is involved in social computations, specifically strategic social reasoning and theory of mind. The authors compared strategic game play in a previously validated social coordination game, the inspection task, in mPFC lesion patients, lesion control patients, and matched healthy controls.

The strengths of this paper are 1) the large patient sample size, 2) the validated task, and 3) the computational model that allows the author to disentangle latest cognitive contributions to overall performance. Notably, the authors have made the data and code available on OSF, which definitely increases the value of the contribution. The OSF site could be improved by README files in each folder that explain the data columns in each csv and .R file.

The results of the study are compelling: the authors find no evidence of altered k-level reasoning in mPFC lesion patients compared to lesion and healthy controls. However, medial prefrontal damage was significantly related to increased boundaries (caution), drift rate (i.e., evidence accumulation/conflict), and non-decision time (perceptual and motor processing).

I have only minor comments and suggestions.

Please show more of the behavioral data, in addition to overall reward and RT, and prior to the model fit. For example, it would be useful to illustrate a sequence of rounds compatible with high fictious play but low influence; versus a sequence of rounds well fit by high influence. I would also like to see a plot of cumulative reward over rounds for each group, rather than just the overall average sum.

The model comparison figure in the supplement, figure S2, does a great job of showing relative consistency in model performance across patient groups. I think this is a nicer plot to include in the main manuscript, in Fig. 3. instead of collapsed across groups. Including it in the main text may help convince readers that the influence parameters improve model performance across patient groups. Is it possible to include individual participants' data in the BIC plots, as points overlaid on the overall fits?

The methods section includes the statement "three other experimental tasks were reported elsewhere." Please add citations, because these other data could be relevant for understanding the overall cognitive profile of these groups. It would also help to know, for reference, whether this patient group showed deficits on any other standard decision making task. If these patients are not impaired in anything, that would confuse the interpretation of the lack of impairment in k-level reasoning.

Were these analyses confirmatory or exploratory? These claims would be particularly strong if preregistered. If some part of the analysis was exploratory, or if other analyses were attempted in addition to those reported, that would be useful to know in interpreting these results.

Finally, there is one matter of rhetoric. The authors name their finding "preserved mentalising". While this broad generalization, treating mentalising as a single construct, is consistent with the vast majority of the prior literature, personally I think it's unhelpful. Recent meta-analyses suggest that existing "mentalising" tasks do not have strong construct validity (Yeung et al 2024) fwiw. The k-level parameter of influence play in this game captures one narrow aspect of mentalising / theory of mind. Without in any way diminishing the importance of this result, the claim is too general. Prior fMRI evidence of

MPFC involvement in social reasoning would not necessarily have identified k-level in the influence game as the most likely role of MPFC within mentalising. MPFC seems to be particularly recruited for aspects of mentalising connected to valence, value and self-relevance — understanding others' emotions, valuing certain others, considering what others value in the self. It may be possible to engage in influence play in these games without relying on the aspects of mentalising that particularly require MPFC.

(Remarks on code availability)
The OSF site could be improved by README files in each folder that explain the data columns in each csv and .R file.

Version 1:

Reviewer comments:

Reviewer #1

(Remarks to the Author)
I would like to thank the authors for addressing all my comments. Congratulations to this fine piece of work, which will have a considerably influence on the literature.

(Remarks on code availability)

Reviewer #2

(Remarks to the Author)
The authors have done a fantastic job in revising this manuscript and I just want to mention one further point.

Thank you for elaborating on the tests done to probe general cognitive deficits in the participants. Given how crucial language capabilities are, I would still think it would have been important to also include a test there and would encourage the authors to mention this in the limitations.

(Remarks on code availability)

Reviewer #3

(Remarks to the Author)
I co-reviewed this manuscript with one of the reviewers who provided the listed reports. This is part of the Nature Communications initiative to facilitate training in peer review and to provide appropriate recognition for Early Career Researchers who co-review manuscripts.

(Remarks on code availability)

Reviewer #4

(Remarks to the Author)
I am satisfied that the authors have addressed my review and the suggestions and questions of the author reviewers, with their extensive thoughtful responses. I recommend publication.

(Remarks on code availability)

Open Access This Peer Review File is licensed under a Creative Commons Attribution 4.0 International License, which permits use, sharing, adaptation, distribution and reproduction in any medium or format, as long as you give appropriate credit to the original author(s) and the source, provide a link to the Creative Commons license, and indicate if changes were made.
In cases where reviewers are anonymous, credit should be given to 'Anonymous Referee' and the source.
The images or other third party material in this Peer Review File are included in the article's Creative Commons license, unless indicated otherwise in a credit line to the material. If material is not included in the article's Creative Commons license and your intended use is not permitted by statutory regulation or exceeds the permitted use, you will need to obtain permission directly from the copyright holder.
To view a copy of this license, visit https://creativecommons.org/licenses/by/4.0/

REVIEWER COMMENTS

Reviewer #1 (Remarks to the Author):

Hsieh et al. use an established social interaction task to test if patients with lesions in MPFC regions or in control regions show altered social decision-making. Overall, their results do not support altered choice behaviour (using standard statistical methods and computational models). But RTs in the group with MPFC lesions tend to be longer, which is mirrored in the decision boundary parameter in the winning drift diffusion model variant.
In my view, the article addresses an important, timely question with well-suited experimental and analytical procedures. Please find my suggestions for improvement below. Sometimes crucial information seems to be buried in the supplementary materials.

Response: Thank you very much for your thoughtful and constructive feedback on our manuscript. We greatly appreciate your positive evaluation of our work, and we are grateful for the time you invested in reviewing it. Your comments have helped us to further clarify our analyses and improve the presentation of key information, allowing us to better communicate our findings and their implications.

R1.1. RTs: Given that the group difference relies largely on RTs, it would be important to see a few more details:

R1.1.a. Could you show some plots with the distribution of RTs per participant and describe these in general? It should be shown that the effect is not driven by some extreme values (i.e., neither extreme individuals nor extreme trials).
R1.1.b. Did you exclude any extremely short or long RTs?
R1.1.c. Often RTs are log-transformed. Are there any specific reasons for not doing this here?
R1.1.d. In general, it would be good to use LMEs here. If I understood correctly from the supplementary information, you did this for analysing the effects of 2nd order beliefs on RTs. Why not for the main analyses?

Response: Thank you for these suggestions. We agree that further analysis of the RT group differences would be helpful.

First, we did not initially exclude extremely short or long response times (**R1.1.b**). However, we agree that evaluating the impact of long or short RTs is valuable. We therefore conducted additional robustness analyses applying a trimming procedure, excluding trials below the 5th and above the 95th percentiles of each participant's RT distribution (Berger & Kiefer, 2021; Ratcliff, 1993). Importantly, the pattern of RT results remained unchanged after trimming. In addition, we re-analysed the RT data using robust linear mixed-effects models (**R1.1.d**), which explicitly account for within-participant variability via random effects and are less sensitive to the influence of extreme observations at the trial level. These analyses yielded convergent results, further indicating that the observed group differences are not driven by extreme RTs. Regarding the log-transformation of RTs (**R1.1.c**), we chose to analyse untransformed RTs to maintain interpretability and consistency with the drift-diffusion modelling framework, which explicitly decomposes RT into additive decision and non-decision components. However, we have now run an additional analysis demonstrating that results remain the same using log-transformed RTs (see details below).

Finally, we now provide an additional plot (see **Figure S3** and below) showing the distribution of RTs at the individual level (**R1.1.a**). Examination of participant-level median RTs revealed that, although a small number of participants exceeded a conservative 2.5 SD criterion within their respective group, these individuals were distributed across all groups rather than being specific to the mPFC group. Excluding these participants in robustness analyses did not

change the pattern of results, indicating that the observed group differences are not driven by extreme individuals. We also conducted distributional analyses at the trial level using permutation-based non-parametric tests to assess whether the effects could be driven by extreme trials rather than reflecting systematic differences (see **Table S6** and below). These analyses indicate that RT differences reflect distribution-level shifts rather than being driven by a small subset of extreme trials.

Taken together, these converging analyses support the robustness of the RT effects and demonstrate that they are not driven by outliers. We have added the results of these analyses to the main manuscript and supplementary materials:

> ***Results:***
> ***Patients with brain damage demonstrate preserved performance but slower response times in strategic social interactions***
> *(…). Additional robustness analyses examining the distribution of response times, censoring trials and individuals with extreme RTs, log-transforming RTs, and using a mixed-effects model are reported in the* ***Supplementary Results*** *(**Table S2-S6**) and confirm that the RT effects are robust. (…).*
>
> ***Supplementary Results:***
> ***Robustness analyses of response time (RT) effects***
> *To assess the robustness of the observed group differences in response times (RTs), we conducted a series of additional analyses addressing the potential influence of extreme trials, extreme individuals, the analytic approach, and distributional assumptions.*
>
> ***Extreme RTs trimming.*** *In the main analyses, RTs were analysed without excluding extreme values. To examine whether the results were driven by unusually short or long RTs, we performed a trimming procedure in which trials below the 5th percentile and above the 95th percentile of each participant's RT distribution were excluded (Ratcliff, 1993). The pattern of group differences in RTs remained unchanged following trimming, indicating that the effects were not driven by extreme trials (see* ***Table S2****).*
>
> ***Log-transformation of RTs.*** *We analysed untransformed RTs in the main analyses to maintain interpretability and consistency with the drift–diffusion modelling framework, which explicitly decomposes RT into decision-related and non-decision components. As a robustness check, we repeated the main analyses using log-transformed RTs. These analyses yielded the same pattern of results as the untransformed analyses, confirming that the findings did not depend on the scale of RT measurement (see* ***Table S3****).*
>
> ***Robust linear mixed-effects modelling.*** *To further account for within-participant variability and to reduce sensitivity to extreme observations, we re-analysed the RT data using robust mixed-effects models with random intercepts for participants that downweigh the influence of extreme trial-level observations. The approach yielded results consistent with the main analyses, demonstrating that the observed group differences in RTs are robust to alternative modelling strategies (see* ***Table S4****).*
>
> ***Distributional analyses at the participant and trial levels.*** *We additionally examined RT distributions at both the participant and trial levels. Inspection of participant-level median RTs revealed that a small number of participants exceeded a conservative 2.5 SD criterion within their respective groups (N: HC = 2, mPFC = 1, LC = 2); however, these individuals were distributed across all groups rather than being specific to the*

*mPFC group (see **Figure S3** for participants' median RT distribution). Excluding these participants from the analyses did not alter the pattern of results (see **Table S5**). At the trial level, we conducted distributional analyses using permutation-based non-parametric tests to assess whether group differences could be attributed to a small subset of extreme RTs (Marozzi, 2013; Székely & Rizzo, 2013). Pairwise comparisons using Kolmogorov–Smirnov tests confirmed distributional differences between the mPFC group and both control groups, whereas differences between HC and LC were comparatively small (mPFC vs HC: D = 0.139, p < 0.001; mPFC vs LC: D = 0.149, p < 0.001; HC vs LC: D = 0.034, p = 0.024). Permutation-based energy tests yielded a similar pattern (see **Table S6**). These analyses indicated that RT differences reflect distribution-level shifts rather than being driven by a limited number of extreme trials.*

Across all robustness analyses—including trimming, transformation, mixed-effects modelling, and distributional assessments—the pattern of RT results remained consistent. Together, these findings demonstrate that the observed RT effects are robust and not driven by extreme trials, extreme individuals, or specific analytic choices.

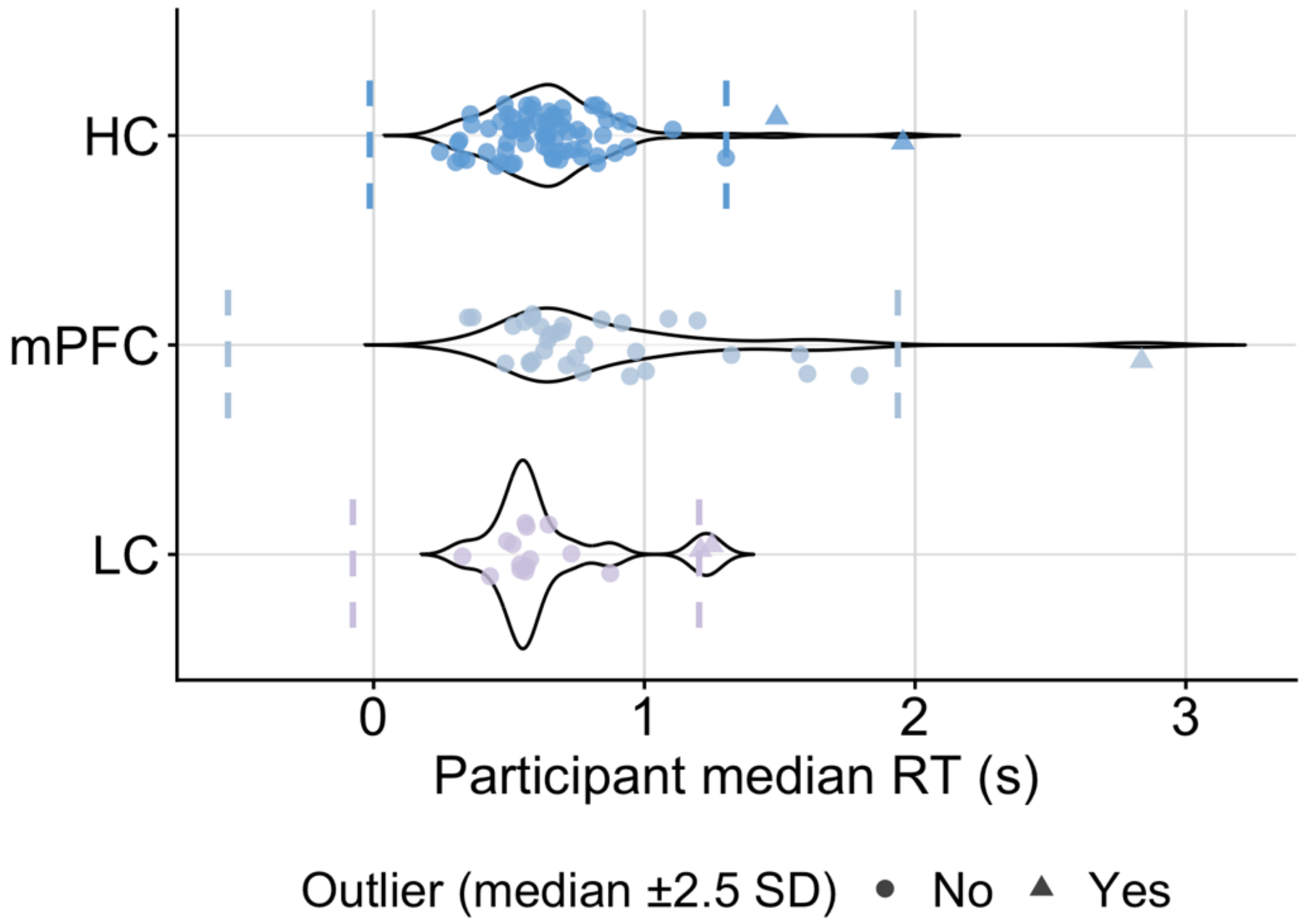

Figure S3. Distribution of participants' median response times across groups. *Violin plots show the distribution of median response times (RTs) for individual participants in each group. Circles indicate participants whose median RT fell within a conservative ±2.5 SD range relative to the group median, whereas triangles indicate participants whose median RT exceeded this criterion. Vertical dashed lines denote the corresponding cut-off values (group median ±2.5 SD) calculated separately for each group. A small number of participants exceeded a conservative 2.5 SD criterion within their respective groups; however, these individuals were present across all groups (see **Figure 2D** for the main RT analysis). HC: healthy control group; mPFC: mPFC lesion patients; LC: lesion control patients.*

Table S2. Robust linear mixed-effects model predicting RT after trimming extreme trials.

Parameters	Coefficient	SE	CI low	CI high	*t*	*p*-value
(Intercept)	0.81	0.04	0.74	0.89	21.39	< .001

Group (mPFC vs HC)	-0.12	0.05	-0.21	-0.03	-2.68	0.007
Group (mPFC vs LC)	-0.15	0.07	-0.28	-0.02	-2.25	0.025

Note. mPFC: mPFC lesion patients, HC: Healthy controls, LC: Lesion controls.

Table S3. Robust linear mixed-effects model predicting RT after log-transformed RTs.

Parameters	Coefficient	SE	CI low	CI high	*t*	*p*-value
(Intercept)	-0.24	0.06	-0.35	-0.13	-4.33	< .001
Group (mPFC vs HC)	-0.18	0.07	-0.31	-0.04	-2.59	0.010
Group (mPFC vs LC)	-0.22	0.10	-0.41	-0.02	-2.21	0.027

Note. mPFC: mPFC lesion patients, HC: Healthy controls, LC: Lesion controls.

Table S4. Robust linear mixed-effects model predicting RT.

Parameters	Coefficient	SE	CI low	CI high	*t*	*p*-value
(Intercept)	0.83	0.04	0.76	0.91	22.02	< .001
Group (mPFC vs HC)	-0.12	0.05	-0.21	-0.03	-2.68	0.007
Group (mPFC vs LC)	-0.15	0.07	-0.28	-0.02	-2.27	0.024

Note. mPFC: mPFC lesion patients, HC: Healthy controls, LC: Lesion controls.

Table S5. Robust linear mixed-effects model predicting RT excluding extreme participants.

Parameters	Coefficient	SE	CI low	CI high	*t*	*p*-value
(Intercept)	0.81	0.03	0.75	0.88	23.59	< .001
Group (mPFC vs HC)	-0.12	0.04	-0.20	-0.03	-2.79	0.005
Group (mPFC vs LC)	-0.18	0.06	-0.30	-0.05	-2.84	0.005

Note. mPFC: mPFC lesion patients, HC: Healthy controls, LC: Lesion controls.

Table S6. Trial-level distributional comparisons of RT between groups.

Comparisons	KS D	KS *p* (Holm)	Energy statistic	Energy *p* (Holm)
mPFC vs HC	0.139	<.001	93.17	0.0015
mPFC vs LC	0.149	<.001	80.48	0.0015
HC vs LC	0.034	0.024	2.11	0.010

Note. mPFC: mPFC lesion patients, HC: Healthy controls, LC: Lesion controls. KS D values indicate the maximum difference between cumulative distribution functions in Kolmogorov–

Smirnov tests. Energy statistics were obtained from permutation-based two-sample energy tests. Reported p values are Holm-adjusted for multiple comparisons.

R1.1.e. Did the RTs become faster over the course of the experiments? This is often the case. Could it be that the group with MPFC lesions did not “adapt” to the task as swiftly as the controls?
R1.1.f. Relatedly: Did the groups differ in the time they needed to understand the task? This time for the instruction is usually not measured but maybe some participants had to repeat the training session and some might have much longer RTs in the training sessions.

Response: Thank you for this insightful question. We have now further examined trial-wise changes in RT using mixed-effects models, including trial progression and its interaction with group. While RTs generally decreased over time across participants, we found no evidence for a group-by-time interaction, indicating that the mPFC lesion group did not adapt more slowly to the task than control groups. We have added these results to the **Supplementary Results** (**Table S7**), and please see below for details.

There were no differences among participants in their understanding of the instructions, either with respect to timing or content. In our task, all participants were required to answer comprehension questions correctly before proceeding to the practice and main task phases. Participants only proceeded once they had answered all comprehension questions correctly, as confirmed by the experimenter. All participants completed the practice tasks consisting of (no more than 10) trials, which were not repeated since it was confirmed that the task was understood before the practice began. We have now included further information in the **Materials and Methods** section.

In addition, we have run further analyses of response times during the practice phase to test if group differences were already present (using robust linear mixed-effects models). RTs were consistently longer during the practice phase than during the main task in all groups ($\beta = 0.88$, SE = 0.02, $t = 36.61$, $p < .001$). Moreover, the mPFC lesion group showed a larger reduction in RT from the practice to the main task compared to healthy controls ($\beta = -0.14$, SE = 0.03, $t = -5.00$, $p < .001$), while not differing significantly from the lesion control group ($\beta = -0.08$, SE = 0.04, $t = -1.80$, $p = .072$; see **Supplementary Results**, **Table S8**). These results suggest that evidence of group differences is present early, but should also be interpreted with caution, given the small number of trials in practice section for RT analyses. We have added these results to the **Supplementary Materials and signposted them in the main manuscript**:

> ***Results:***
> ***Patients with brain damage demonstrate preserved performance but slower response times in strategic social interactions***
> *(…). Complementary analyses of trial-wise RT changes and practice-phase performance indicate that these effects do not reflect slower task adaptation or delayed task understanding (see **Supplementary Results**, **Table S7-S8**).*
>
> ***Materials and Methods:***
> ***Procedure***
> *Participants then read the ‘inspection game’ task instructions from the screen and filled out a comprehension questionnaire. Participants were only allowed to proceed once all comprehension questions were answered correctly, which was verified by the experimenter. They completed a short practice task to familiarise them with the required response time and were told that during this practice, responses were*

randomly chosen by a computer. The practice phase was not repeated, as task understanding was confirmed prior to its onset. (…).

Supplementary Results:

Task adaptation and practice-phase response times

Trial-wise changes in response time. *To examine whether response times (RTs) changed over the course of the experiment, and whether groups differed in their rate of adaptation, we fitted robust linear mixed-effects models including trial progression and its interaction with group. Across participants, RTs decreased reliably over trials, indicating general task adaptation. Crucially, there was no evidence for a group-by-trial interaction, suggesting that participants with mPFC lesions did not adapt more slowly to the task than healthy or lesion control groups (**Table S7**).*

Response times during the practice phase. *We additionally examined RTs during the practice phase to assess whether group differences in RT might reflect differences in early task engagement or familiarisation. RTs were longer during the practice phase than during the main task across all groups, consistent with participants familiarising themselves with the task structure. The mPFC lesion group showed a larger reduction in RT from the practice to the main task compared to healthy controls, while not differing significantly from lesion controls (**Table S8**).*

Together, these analyses indicate that the observed group differences in RT are unlikely to be explained by slower task adaptation or delayed familiarisation in the mPFC group. These findings should, however, be interpreted with caution, given the relatively small number (<10) of practice trials available for RT analysis.

Table S7. Robust linear mixed-effects model examining trial-wise changes in RT and group differences in task adaptation.

Parameters	Coefficient	SE	CI low	CI high	*t*	*p*-value
(Intercept)	0.83	0.04	0.76	0.91	22.06	< .001
Group (mPFC vs HC)	−0.12	0.05	−0.21	−0.03	−2.67	0.008
Group (mPFC vs LC)	−0.15	0.07	−0.28	−0.02	−2.26	0.024
Trial N	−0.05	0.01	−0.06	−0.04	−9.24	< .001
Group (mPFC vs HC) × Trial N	0.000	0.006	−0.01	0.01	−0.11	0.911
Group (mPFC vs LC) × Trial N	−0.01	0.01	−0.03	0.00	−1.55	0.122

Note. mPFC: mPFC lesion patients, HC: Healthy controls, LC: Lesion controls. Trial N: Trial number.

Table S8. Robust linear mixed-effects model comparing RTs between practice and main task phases.

Parameters	Coefficient	SE	CI low	CI high	*t*	*p*-value
(Intercept)	0.84	0.04	0.77	0.92	21.82	< .001

Phase (Practice vs Main)	0.88	0.02	0.83	0.92	36.61	< .001
Group (mPFC vs HC)	−0.12	0.05	−0.21	−0.03	−2.65	0.008
Group (mPFC vs LC)	−0.15	0.07	−0.28	−0.02	−2.26	0.024
Phase × Group (mPFC vs HC)	−0.14	0.03	−0.20	−0.09	−5.00	< .001
Phase × Group (mPFC vs LC)	−0.08	0.04	−0.16	0.01	−1.80	0.072

Note. mPFC: mPFC lesion patients, HC: Healthy controls, LC: Lesion controls.

R1.1.g. On page 13 you write: "Our behavioural analysis showed that mPFC damage was in general associated with preserved mentalising but slower decision-making, due to higher boundaries for the evidence accumulation process during strategic interactions." This causal language is not warranted at this point in the manuscript.

Response: Thank you for this important comment. We have revised the wording to remove causal language and to more accurately reflect the descriptive nature of the behavioural findings:

> ***Results:***
> ***Damage to mPFC subregions impacts on boundary, drift rate temperature, and non-decision time***
> *Our behavioural analysis showed that mPFC damage was associated with preserved mentalising but slower decision-making during strategic interactions, captured in our model by higher boundaries in the evidence accumulation process. (…).*

R1.1.h. Related to 1d: Why did you use a median split? I can understand that this is easier to plot (cf. Fig. S5). Do the results hold when using the full range of (absolute) updates?

Response: Thank you for your query. The median split was indeed used to aid visualisation and to provide an intuitive summary of effects across conditions. We have now also conducted the analyses using the full continuous range of absolute belief updates and found the same pattern of effects. We have added these new results to the **Supplementary Materials**:

> ***Results:***
> ***mPFC damage preserves mentalising and leads to a higher boundary of evidence accumulation***
> *(…). We also ran an additional exploratory analysis to examine how RT latencies reflected belief updating from the computational model between groups. This additional analysis suggested participants with mPFC lesions tended to exhibit larger second-order belief updates on faster-response trials, a pattern that was consistent across alternative model specifications (see **Supplementary Results**, **Figure S11**, and **Table S15**).*

> ***Supplementary Results:***

Response time patterns reflect second-order belief updating and strategic deliberation in mPFC patients

*The model revealed a significant interaction between update and group (…). Repeating the analysis with continuous belief updates yielded the same qualitative pattern of results as the median-split approach, confirming that the reported effects are not an artefact of binning (**Table S15**).*

Table S15. Robust linear mixed-effects model predicting RT from continuous second-order belief updates magnitude

Parameters	Coefficient	SE	CI low	CI high	*t*	*p*-value
(Intercept)	0.83	0.04	0.76	0.91	22.21	< .001
Group (mPFC vs HC)	−0.12	0.05	−0.21	−0.03	−2.68	0.007
Group (mPFC vs LC)	−0.15	0.07	−0.28	−0.02	−2.28	0.023
Belief updates	−0.02	0.01	−0.03	−0.01	−4.30	< .001
Group (mPFC vs HC) × Belief updates	−0.02	0.01	0.00	0.03	2.63	0.009
Group (mPFC vs LC) × Belief updates	−0.01	0.01	−0.01	0.02	0.71	0.480

Note. mPFC: mPFC lesion patients, HC: Healthy controls, LC: Lesion controls.

R1.1.i. I find Fig. S3A & 3B quite informative (both for RTs and for choices). However, here it is unclear which data points are plotted exactly. It could be helpful to make these plots separately for the three groups.

Response: Thank you for this suggestion. We have now added more detailed explanations to the figure captions to make it clearer which data points are plotted, and we have also provided the posterior predictive plots separated by group in the **Supplementary Materials**:

Supplementary Results:
Model validation confirms that influence-DDM captures both choice probability and RT patterns

*(…). Additional posterior predictive plots separated by group are shown in **Figure S5E-S5F**.*

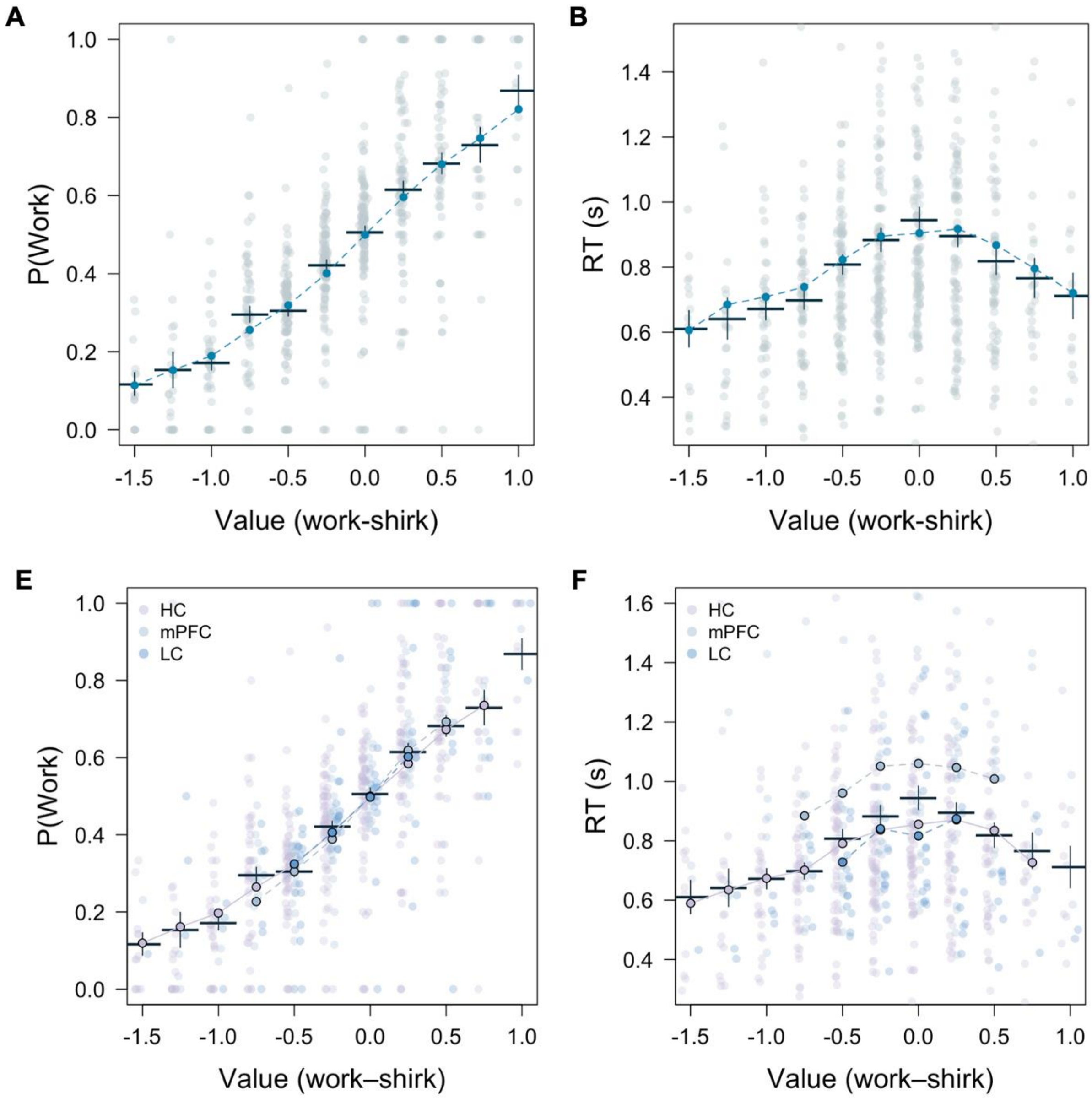

Figure S5. Model accurately reproduces choice and RT patterns across participants. *The posterior predictive check of the winning influence mode shows that the model can accurately predict (A) the participant's choice and (B) their response time (RT). Transparent light blue dots represent binned trial-level behavioural data averaged within participants, with the black line indicating the group-level average across participants and their error bars (error bars indicate ±1 SEM). Solid points show the group-level average of the model-simulated data across participants. (C) The predictions of the fitted influence learning model for each participant show a robust positive correlation with the observed probabilities of choosing 'work', P(Work), and (D) the response times (RTs) (see* ***Figure 3B*** *for parameter recovery results). (E-F) The same posterior predictive checks shown separately for each group (HC, mPFC, LC), with group membership indicated by colour as shown in the figure. p = p-value.*

R1.2. Control questionnaires and tasks:

R1.2.a. Did participants perform some sort of non-social control task? If so, did you look into RTs?

Response: Thank you for this query. Participants completed standard neuropsychological assessments indexing non-social cognitive processes, including the Trail Making Test, Addenbrooke's Cognitive Examination, and the Raven Standard Progressive Matrices, which are reported in the revised manuscript and Supplementary Materials (see also **R1.2.c**). These measures are not trial-based decision-making tasks and therefore do not afford direct RT comparisons with the inspection game. However, to address the possibility that the observed RT effects reflect more general cognitive ability or non-social processing speed, we conducted additional control analyses in which both Raven Standard Progressive Matrices (assessing abstract visuospatial reasoning) and Trail Making Test (visual attention and non-social processing speed) performance were included as covariates in the RT models. Importantly, the pattern of group differences in RT remained unchanged after accounting for these variables. We now clarify this approach in the manuscript and note the absence of a fully matched non-social trial-level task as a limitation:

> ***Results:***
> ***Patients with brain damage demonstrate preserved performance but slower response times in strategic social interactions***
> *(…). Therefore, although mPFC damage was associated with slower response times, performance remained intact. This pattern remained unchanged when controlling for individual differences in general cognitive ability and non-social processing speed, as indexed by Raven Standard Progressive Matrices and Trail Making Test performance (see **Supplementary Results**, **Table S9-S10**). (…).*
>
> ***Discussion:***
> *Despite employing a validated interactive social task and a relatively large lesion sample, several limitations should be acknowledged. (…). Finally, the present approach reflects an inherent trade-off between experimental control and ecological richness. While the controlled and anonymised interaction enabled isolation of social strategic belief-based reasoning, and additional control analyses mitigate concerns about general cognitive impairment, the task does not fully capture the richness of naturalistic social interaction, and standard neuropsychological measures do not provide trial-level non-social benchmarks for directly comparing decision dynamics. Even with these limitations, the present work represents a significant advance beyond traditional ToM paradigms[124–126] by integrating a more grounded interactive task with computational modelling and lesion-symptom mapping to capture dynamic strategy-based social interactions that require mentalisation.*
>
> ***Supplementary Results:***
> ***Neuropsychological measures and cognitive control analyses***
> *Group comparisons on general cognitive measures showed that the mPFC lesion group scored lower than healthy controls on the Raven Standard Progressive Matrices (RSPM), while not differing significantly from the lesion control group. A similar pattern was observed for global cognitive functioning as assessed by the Addenbrooke's Cognitive Examination (ACE-III), which was administered to lesion patients and an age-matched subset of healthy controls (>40 years). In contrast, performance on the Trail Making Test (Parts A and B), which indexes visual attention and non-social processing speed, did not differ significantly between the three groups. This pattern is consistent with expectations in neurological samples and indicates that, while patients differed from healthy controls on some general cognitive measures, the two lesion groups were broadly matched, particularly on measures most relevant to response speed (see **Table S1**).*

*To assess whether the observed group differences in response time (RT) could be explained by general cognitive ability or non-social processing speed, we conducted an additional control analysis including both RSPM scores and Trail Making Test performance as covariates in the RT models. The main effect of group on RT remained significant, and the pattern and magnitude of group differences were unchanged (**Table S9**). Inclusion of these covariates did not alter any other key findings: there were still no group differences in overall payoff (**Table S10**), and group comparisons of model-derived parameters yielded the same pattern of results as in the primary analyses (**Table S11**). Together, these results indicate that the slower decision-making observed in mPFC patients cannot be explained by differences in general cognitive ability or non-social processing speed alone, and that the main behavioural and computational conclusions remain robust after accounting for these factors.*

Table S9. Robust linear mixed-effects model predicting RT with cognitive covariates.

Parameters	Coefficient	SE	CI low	CI high	*t*	*p*-value
(Intercept)	0.74	0.08	0.69	0.90	9.43	<.001
Group (mPFC vs HC)	-0.10	0.05	-0.19	-0.01	-2.24	0.025
Group (mPFC vs LC)	-0.14	0.06	-0.27	-0.02	-2.28	0.023
Ravens	-0.02	0.01	-0.04	0.00	-2.33	0.020
Trail Making Part A	0.00	0.00	0.00	0.01	2.32	0.020
Trail Making Part B	0.00	0.00	0.00	0.00	1.26	0.209

Note. mPFC: mPFC lesion patients, HC: Healthy controls, LC: Lesion controls. Three observations were excluded due to missing data.

Table S10. Control analyses of group differences in mean payoff (ANCOVA).

Effect	df	Sum Sq	Mean Sq	*F*	*p*-value
Group	2	8.4	4.210	0.532	0.589
Ravens	1	5.7	5.699	0.720	0.398
Trail Making Part A	1	27.2	27.170	3.434	0.067
Trail Making Part B	1	14.3	14.270	1.803	0.182
Residual	113	894.2	7.913		

Note. Three observations were excluded due to missing data.

Table S11. ANCOVA results for model-derived parameters controlling cognitive measurements.

Effect	df	Sum Sq	Mean Sq	*F*	*p*-value
Decision boundary					
Group	2	0.844	0.422	3.227	0.043
Ravens	1	1.057	1.057	8.074	0.005
Trail Making Part A	1	1.053	1.053	8.047	0.005
Trail Making Part B	1	0.202	0.203	1.547	0.216

Residual	113	14.787	0.131		
Drift rate temperature					
Group	2	0.002	0.001	2.630	0.077
Ravens	1	0.002	0.002	4.905	0.030
Trail Making Part A	1	0.002	0.002	4.560	0.035
Trail Making Part B	1	0.000	0.000	0.016	0.900
Residual	113	0.052	0.000		
Non-decision time					
Group	2	0.012	0.006	0.520	0.596
Ravens	1	0.015	0.015	1.328	0.252
Trail Making Part A	1	0.015	0.014	1.397	0.240
Trail Making Part B	1	0.014	0.011	1.253	0.265
Residual	113	1.250			
Fictitious play					
Group	2	0.034	0.017	0.124	0.884
Ravens	1	0.018	0.018	0.132	0.717
Trail Making Part A	1	0.166	0.166	1.209	0.274
Trail Making Part B	1	0.576	0.576	4.198	0.043
Residual	113	15.492	0.137		
Influence play					
Group	2	0.018	0.001	0.058	0.944
Ravens	1	0.071	0.071	0.455	0.502
Trail Making Part A	1	0.059	0.059	0.375	0.542
Trail Making Part B	1	0.623	0.623	2.968	0.049
Residual	113	17.734	0.157		

Note. Three observations were excluded due to missing data.

R1.2.b. You write: “After the series of tasks was done (three other experimental tasks were reported elsewhere), participants completed a range of questionnaires (see below and Supplementary Experimental Procedures)” Which ones? You don’t give any citations for the “reported elsewhere.”

Response: Thank you for pointing this out. We have updated the manuscript to include citations for the tasks reported elsewhere:

> ***Materials and Methods:***
> ***Procedure***
> *After the series of tasks was done (three other experimental tasks are reported elsewhere) (Gueguen et al., 2025; Lockwood et al., 2024; Su et al., 2025), participants completed a range of questionnaires (see below and Supplementary Experimental Procedures).*

R1.2.c. You write: “Fluid intelligence was measured using a shortened version of the Raven Standard Progressive Matrices (RSPM) 119. The Addenbrooke’s Cognitive Examination (ACE-III) was used to assess global cognitive functioning across attention, memory, fluency, language, and visuospatial domains in participants over 40 and in both lesion groups 120

(see Supplementary Experimental Procedures for the full descriptions of the assessments)." However, the groups differed in these! You don't mention this anywhere, which seems like hiding a crucial difference! In contrast, the absence of differences for other tests is explicitly mentioned: "In addition, the mPFC patients did not differ significantly from the healthy or lesion control groups in the neuropsychological test assessing cognitive ability and visual attention (Trail Making Test; Part A ps > 0.62, Part B ps > 0.12)."

Response: Thank you for highlighting this important point. We apologise, as we certainly didn't mean to imply that we were hiding any group differences. For these additional assessments, we believe it is most crucial to ensure the two lesion groups are matched on key measures, as due to additional factors, it is not surprising that patients who have experienced a haemorrhage do differ in some respects from healthy controls. This is why, in the main text, we highlighted the measures that were not different between lesion groups and healthy controls, as well as those that were matched between lesion groups, then provided full details in the supplement. We have now revised the main text to explicitly report and discuss the observed group differences in Raven and ACE-III scores. We also clarify the interpretation of these measures in relation to our primary conclusions and note that the key results remain unchanged when accounting for these variables (see the response of **R1.2.a**).

> ***Materials and Methods:***
> ***Participants***
> *The final sample included 122 participants (…). In addition, the mPFC patients did not differ significantly from the healthy or lesion control groups in the neuropsychological test assessing visual attention and processing speed (Trail Making Test; Part A ps > 0.62, Part B ps > 0.12). Participants also completed additional neuropsychological assessments indexing general cognitive ability, including the Raven Standard Progressive Matrices (RSPM) and the Addenbrooke's Cognitive Examination (ACE-III). Group differences were observed on these measures, with lesion patients scoring lower than healthy controls, consistent with broader cognitive consequences of focal brain injury (see **Supplementary Results** and **Table S1**). Importantly, the two lesion groups were broadly comparable on these measures.*

> ***Supplementary Results:***
> ***Neuropsychological measures and cognitive control analyses***
> *Group comparisons on general cognitive measures showed that the mPFC lesion group scored lower than healthy controls on the Raven Standard Progressive Matrices (RSPM), while not differing significantly from the lesion control group. A similar pattern was observed for global cognitive functioning as assessed by the Addenbrooke's Cognitive Examination (ACE-III), which was administered to lesion patients and an age-matched subset of healthy controls (>40 years). In contrast, performance on the Trail Making Test (Parts A and B), which indexes visual attention and non-social processing speed, did not differ significantly between the three groups. This pattern is consistent with expectations in neurological samples and indicates that, while patients differed from healthy controls on some general cognitive measures, the two lesion groups were broadly matched, particularly on measures most relevant to response speed (see **Table S1**).*

R1.3. Modelling:

R1.3.a. The DDM could be explained a bit more in the main text; especially the role of the RTs in the DDM

Response: Thank you for this helpful suggestion. We have revised the main text to provide a clearer explanation of the role of RTs in the drift-diffusion model:

> ***Introduction:***
> *(…). Another key advantage is that these 'online' measures of social cognition, where people make decisions repeatedly over multiple trials, can be combined with computational models of behaviour [28,49–51] to reveal the underlying mechanisms, such as belief updating. Crucially, computational models allow behaviour to be decomposed into latent processes, separating belief-related computations from more general decision dynamics that may also be affected by brain lesions. The participants' specific parameters from these models can then be used with lesion-symptom mapping to identify specific regions that map onto these computational mechanisms [52–54].*
>
> *(…). Given that several studies have linked the mPFC to other cognitive processes such as reaction time latency [55,56], we built a computational model that combined choice (drift-diffusion) and mentalising (belief-updating) processes. In particular, drift–diffusion models (DDM) use both choices and response times to estimate how fast people accumulate evidence during a choice, their threshold for initiating a response, and the time taken for non-decision processes. This allowed us to simultaneously assess a range of latent processes guiding behaviour, thereby providing greater insight into the mechanisms required to successfully interact with others and elucidating the specific behavioural deficits caused by mPFC damage.*
>
> ***Materials and Methods:***
> ***Drift diffusion model (DDM).*** *The drift-diffusion model (DDM) is a cognitive process model that describes the decision-making process between two alternatives by jointly accounting for choices and reaction times [95]. The model assumes that simple decisions between two choices use a sequential sampling framework, with decisions occurring when the accumulated evidence reaches a threshold. Reaction times are therefore informative about the dynamics of evidence accumulation, allowing evidence accumulation speed and response caution to be dissociated. The value of each choice (e.g., the value of choosing 'work'), which was calculated using separate learning models (see below), was scaled by decision noise parameters, drift rate temperature (d):*
>
> $$v_t = d \cdot V_t^{Work}. \quad (1)$$
>
> *The weighted choice values v_t were then input into the DDM model. The decision boundary parameter α in the DDM model captured response caution, with higher values indicating that more evidence was required before committing to a choice. The non-decision time parameter τ was also added to each trial to capture components of response time unrelated to evidence accumulation, such as sensory encoding and motor execution.*

R1.3.b. In the supplementary materials it becomes clear that the "Influence-DDM best fits the mPFC and HC groups, while the LC group favours simpler fictitious-DDM." This has to be explicitly mentioned in the main text and should be carefully discussed.

Response: Thank you for raising this point. The reason we did not include much discussion of this result is that we had not formally compared how much better the fictitious play DDM

was compared to the influence DDM in the LC group. We have now run a new analysis comparing models based on how well they predict participants' choices. Using predictive accuracy, the Influence-DDM actually performs slightly better than the Fictitious-DDM even in the LC group. For this reason, although the fictitious-play DDM has a slightly lower BIC than the influence DDM (ΔBIC < 1.5), we do not consider this a meaningful group difference. We have added this new analysis to the supplementary materials and included additional text in the manuscript explaining this result. In addition, we have included complementary prediction accuracy analyses, which are reported in the Supplementary Materials:

> ***Results:***
> ***Separating mentalising from basic choice mechanisms and perceptual and motor speed using computational modelling***
> *The influence-DDM model was best able to explain behaviour across groups, (…). Although inspection of group-wise BIC values indicated that the lesion control (LC) group showed a marginal preference for the simpler fictitious-DDM, this difference was small and was not supported by predictive accuracy analyses (see* ***Supplementary Results*** *and* ***Figure S6****). (…).*
>
> ***Supplementary Results:***
> ***Influence-DDM best fits the mPFC and HC groups, while the LC group favours simpler fictitious-DDM***
> *As shown in Figure 3C & S4, both the HC group and the mPFC lesion group showed the lowest BIC for the influence-DDM model, suggesting that incorporating second-order belief updating best captured their strategic choices. In contrast, the LC group showed the best fit for the fictitious-DDM model (mean BIC = 282.89), with the influence-DDM yielding a higher BIC value (mean BIC = 284.39). However, this BIC difference was relatively small (ΔBIC < 1.5).*
>
> *To formally assess whether the fictitious-DDM provided a meaningful advantage in the LC group, we conducted additional analyses comparing models based on how well they predicted participants' choices. Predictive accuracy was evaluated by simulating trial-by-trial choices for each participant using their model-derived parameter estimates and comparing these simulated choices with observed choices across trials. Contrary to what might be inferred from the small BIC difference, the influence-DDM showed slightly higher predictive accuracy than the fictitious-DDM even within the LC group (mean difference = 2.74 percentage points ± 0.77 SEM; paired $t_{(15)} = 3.54$, $p = 0.003$;* ***Figure S6A-S6C****; see* ***Figure F6B*** *for the direct comparison between the fictitious-DDM and the influence-DDM).*
>
> *These results indicate that the apparent preference for the fictitious-DDM in the LC group based on BIC alone does not reflect a robust or meaningful difference in the underlying decision strategy. Rather, they suggest that both models capture LC behaviour similarly well, and that the inclusion of influence-based mentalising does not impair model performance in this group. The small sample size of the LC group (N = 16) may also contribute to greater uncertainty in model comparison metrics. Additionally, the heterogeneity of lesion locations in the LC group may have led to more variable strategies that are better captured by simpler belief-tracking mechanisms, rather than the more complex higher-order reasoning implemented in the influence model. Future studies with larger LC samples may help clarify whether this pattern reflects a true cognitive distinction or sample-specific variability. Taken together, these findings support the use of the Influence-DDM as a common computational framework across groups.*

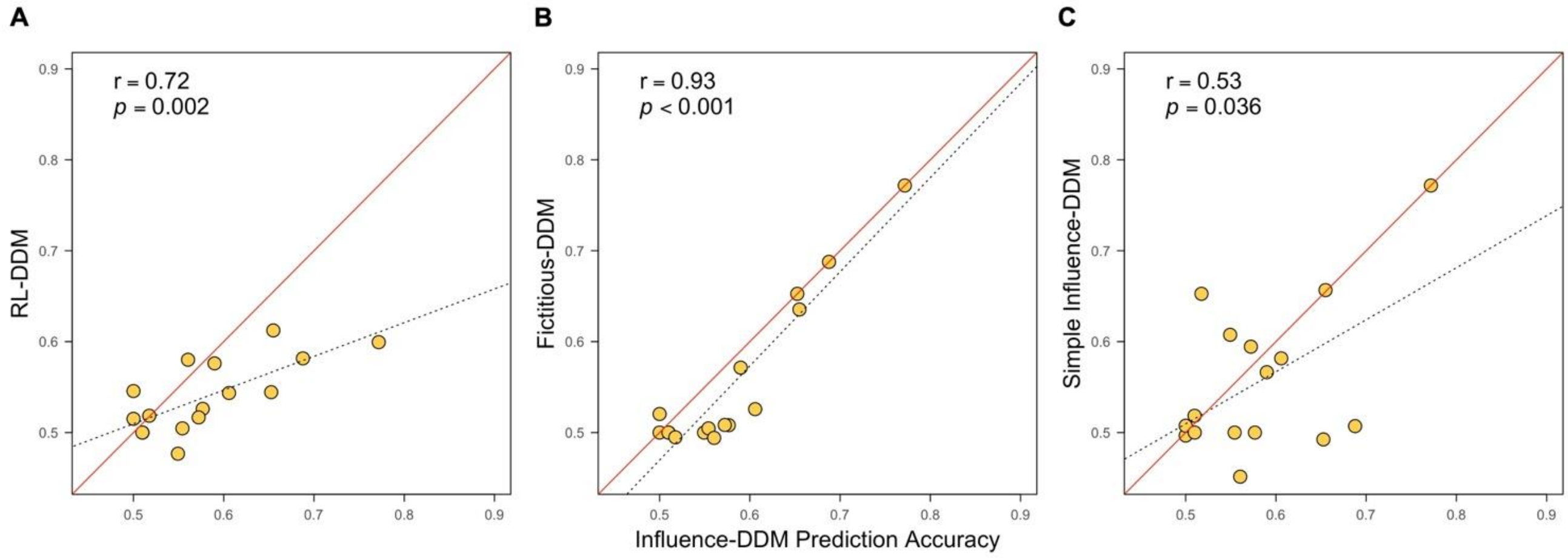

Figure S6. Predictive accuracy comparison between alternative models and the influence-DDM in the lesion control (LC) group. *Scatter plots show participants' trial-by-trial predictive accuracy of the influence-DDM (x-axis) compared with (A) the reinforcement-DDM, (B) the fictitious-DDM, and (C) the simple influence-DDM in the lesion control group (N = 16) (See* ***Figure 3C*** *&* ***S4*** *for the BIC difference of each group). Each point represents an individual participant. The red diagonal line indicates equal predictive accuracy between the two models being compared. The black dotted line indicates the best-fitting linear regression line between the predictive accuracies of the two models across participants. Points below the diagonal indicate higher predictive accuracy for the influence-DDM. Pearson correlation coefficients (r) and corresponding p-values are reported in each panel.*

R1.3.c. Drift rate temperature and non-decision time did not differ between the groups. It should be clearer why it is still warranted to perform VSLM.

Response: Thank you for raising this point. We have clarified in the main text that VLSM was used to assess lesion–parameter relationships at the voxel level, even in the absence of significant group differences, and to capture spatially specific associations that may not be detectable in group comparisons:

Results:
Damage to mPFC subregions impacts on boundary, drift rate temperature, and non-decision time
(…). We next sought to examine whether there were specific regions in the brain where damage was associated with more fine-grained differences in the extent of influence play, fictitious play, or the DDM choice parameters. Given our relatively large sample of brain-lesion patients (N = 49), we employed voxel-based lesion-symptom mapping (VLSM), which tests whether voxel-level damage predicts variability in model-derived parameters across patients, by generating a brain t-statistics map that is independent of group-level differences. The VLSM was conducted using the permutation-based threshold-free cluster enhancement (TFCE) [60,61] *implemented in the FMRIB Software Library (FSL) (see* ***Methods*** *and* ***Supplementary Experimental Procedures****).*

Methods:
We conducted Voxel-based lesion-symptom mapping (VLSM) analyses on the best-fitting parameters of the influence-DDM model: fictitious play η and influence play κ to examine links between specific brain damage and mentalising ability. We also

analysed drift rate temperature d, boundary α, and non-decision time τ, to assess associations with task performance and response latency. The VLSM analyses assess voxel-level associations between lesion location and model-derived parameters and do not rely on the presence of significant group differences in these parameters. This approach therefore allows detection of spatially specific lesion–parameter relationships that may not be evident in group-level comparisons, particularly when effects are confined to smaller subregions or relate to variability within groups.

R1.3.d. Relatedly: How correlated are they with each other and with the decision boundary? That is, how independent are the analyses showing the considerably overlapping regions in Fig. 5?

Response: Thank you for your query. We have now examined correlations between parameters. These showed that the collinearity between parameters was negligible (all VIFs ≈ 1.01). We have added the correlation matrix and VIF results to the Supplementary Materials and updated the results section:

Results:
Separating mentalising from basic choice mechanisms and perceptual and motor speed using computational modelling
*The influence-DDM model had excellent parameter recovery (**Figure 3B**) and could accurately reconstruct participants' choices (see **Supplementary Results** and **Figure S5**). To assess the independence of model parameters, we examined correlations across all five parameters and tested their multicollinearity. These analyses revealed minimal collinearity (all VIFs ≈ 1.01; see **Supplementary Results** and **Figure S8**), indicating that the parameters capture largely independent aspects of behaviour. Therefore, a computational model that incorporated mentalising was best able to describe participants' behaviour across groups, which again supports that mPFC damage does not seem to impair mentalising ability.*

Supplementary Results:
Correlations and collinearity between model parameters
*To assess the degree of dependence between model parameters, we examined pairwise correlations among all five parameters of the Influence-DDM, including the two mentalising parameters (influence play κ and fictitious play η) and the three decision-related DDM parameters (decision boundary α, drift rate temperature d, and non-decision time τ). Spearman correlation coefficients were computed across participants and are shown in **Figure S8**.*

Overall, correlations between parameters were low to modest, indicating that the parameters captured largely distinct aspects of behaviour. To further assess potential multicollinearity among the decision-related parameters, we conducted variance inflation factor (VIF) analyses focusing on the three DDM parameters. Specifically, each DDM parameter was regressed on the remaining two parameters, and VIFs were computed from these models. All VIF values were close to 1 (all VIFs ≈ 1.01), well below conventional thresholds for concern, indicating negligible multicollinearity.

Together, these analyses confirm that the five model parameters are largely independent and that subsequent analyses treating these parameters separately are unlikely to be confounded by shared variance.

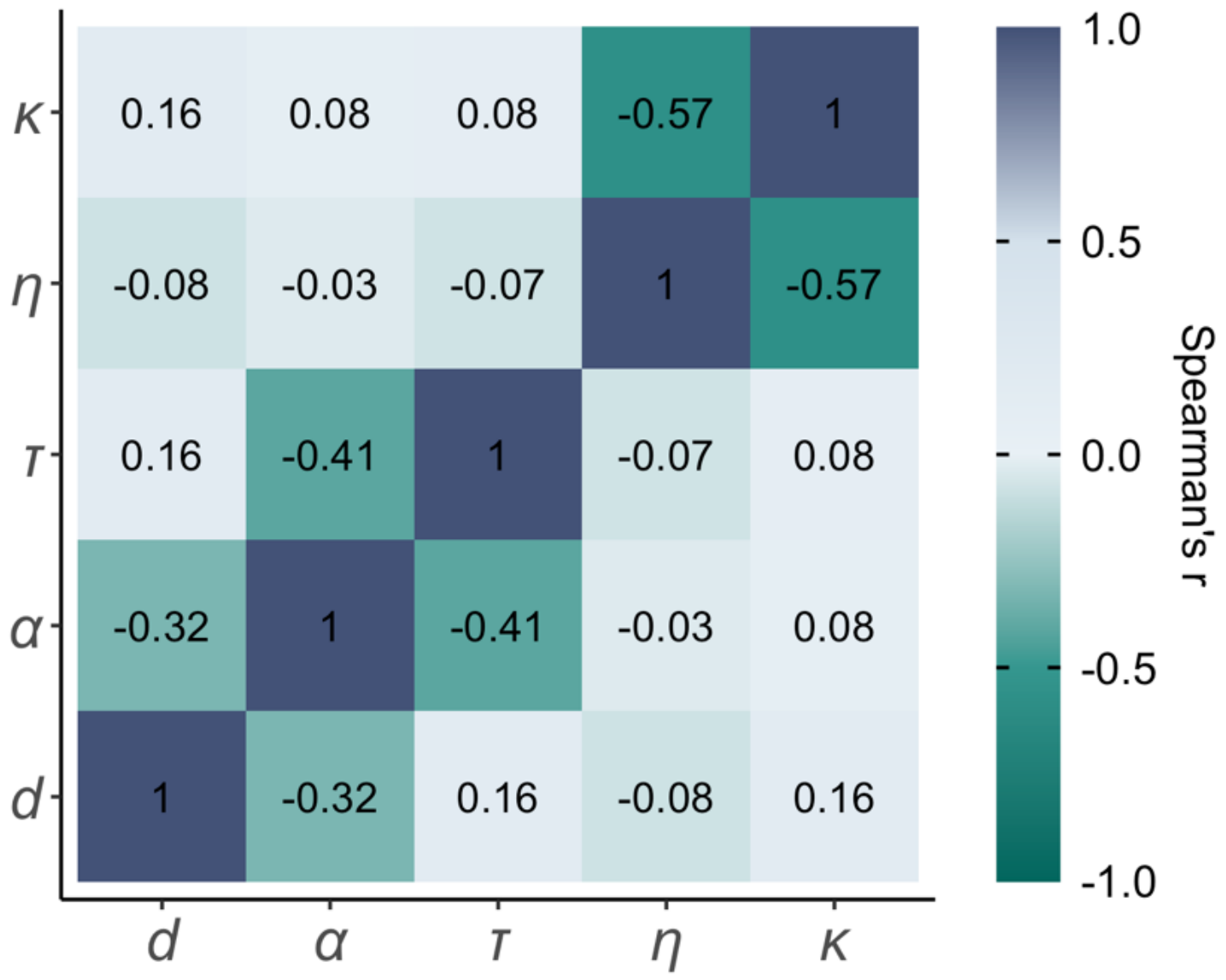

Figure S8. Correlation matrix of model parameters. *Heatmap showing pairwise Spearman correlations between the five model parameters: drift rate temperature (d), decision boundary (α), non-decision time (τ), fictitious play (η), and influence play (κ) (see **Figure 3** for main model fitting results). Values indicate correlation coefficients, with colour intensity reflecting correlation strength. Overall, correlations were low to modest, indicating that the parameters capture largely independent aspects of behaviour. Multicollinearity among the three DDM parameters was further assessed using variance inflation factors (see **Supplementary Results**).*

R1.3.e. Do simulations with different decision boundaries show that the payoffs remain the same?

Response: Thank you for your query. We have now run a simulation analysis where we systematically varied decision boundaries to evaluate the influence on payoff and reported in the **Supplementary Results**. We have also found that the fitted decision boundary parameters were not associated with payoff in the observed data. Additional details are provided in the **Supplementary Results**:

Results:

mPFC damage preserves mentalising and leads to a higher boundary of evidence accumulation

*We also observed no significant difference between the groups in the level of fictitious play η. (…). The other two DDM parameters (drift rate temperature d and non-decision time τ) did not significantly differ between groups (see **Supplementary Results** and **Figure S9**). Although simulations indicated that higher decision boundaries can, in principle, increase payoff, no direct association between fitted decision boundary and payoff was observed in the empirical data, where behaviour reflects the joint influence of multiple model parameters (see **Supplementary Results** and **Figure S10**). These*

findings suggest mPFC patients need more evidence before committing to a decision, aligning with the observed response times differences between the groups, and that all three groups processed the perceptual information similarly and were equally sensitive to evidence for one choice.

Supplementary Results:

Decision boundary, payoff, and simulation analyses

*In the comparisons of model-derived parameters, the mPFC lesion patients exhibited higher decision boundaries, indicating a more cautious evidence accumulation process before making the final decision. In principle, such increased caution could lead to better task performance by reducing costly errors and thereby increasing payoff. However, in the observed data, the decision boundary was not significantly associated with payoff across any of the groups (**Figure S10**).*

To assess whether the decision boundary can, in principle, influence task performance, we conducted simulation analyses in which the decision boundary parameter (α) was systematically varied across three levels (low, medium, and high). These boundary levels were defined relative to each participant's fitted decision boundary, using a fixed offset to generate lower and higher boundary values within a plausible parameter range. All other model parameters were held constant at each individual's fitted values. For each boundary level, choices were simulated across trials, and expected payoff was calculated from the simulated behaviour.

*These simulations revealed that higher decision boundaries led to modest increases in payoff, consistent with a more cautious decision strategy that reduces decision errors (**Table S12**). Quantitatively, increasing the boundary from the lowest to the highest level produced an overall improvement of approximately 2.1% in accumulated expected payoff. This pattern demonstrates that the decision boundary can influence payoff when manipulated in isolation under controlled conditions. By contrast, in the empirical data, boundary differences did not translate into systematic differences in payoff, likely because task performance reflects the joint influence of multiple interacting model parameters rather than the decision boundary alone. Together, the simulation and empirical analyses clarify that the decision boundary primarily captures differences in decision dynamics, rather than differences in overall task performance.*

Supplementary Figures:

A **B** **C**

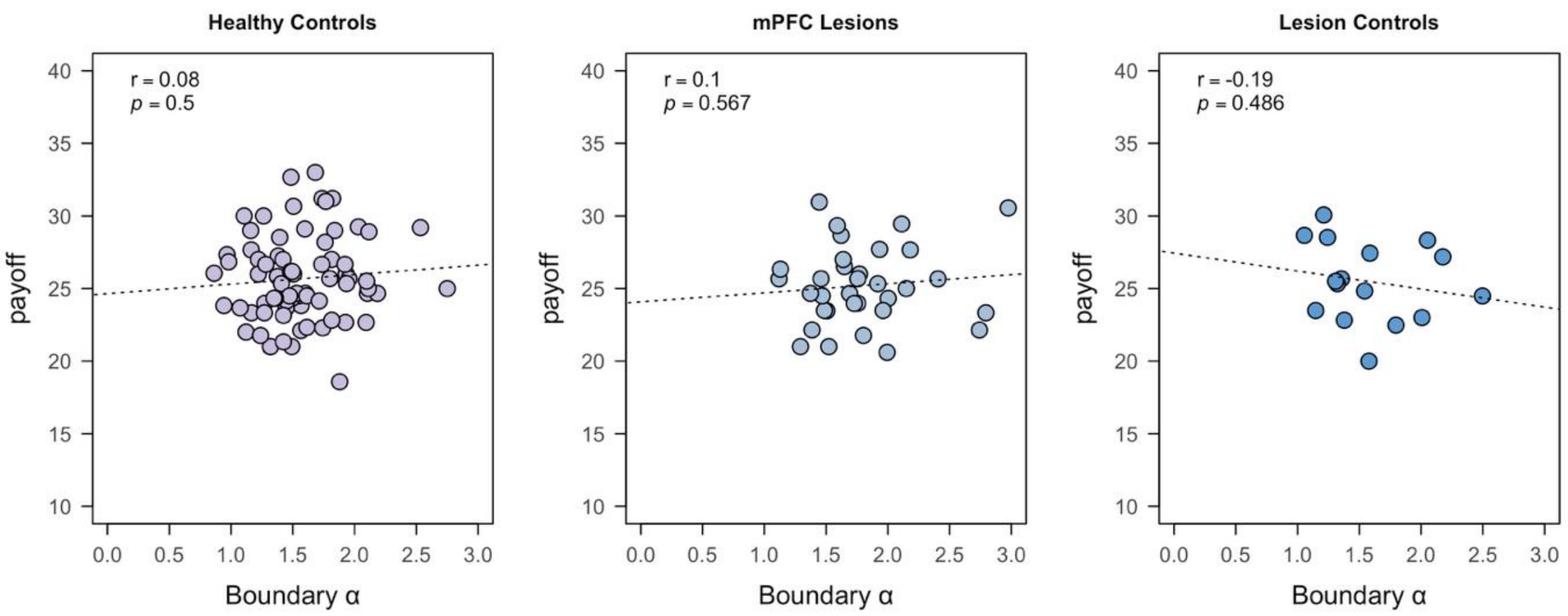

Figures S10. Relationship between fitted decision boundary and payoff across groups. *Scatter plots show the relationship between fitted decision boundary (α) and payoff for (A) healthy controls, (B) mPFC lesion patients, and (C) lesion control participants (right). Each point represents an individual participant. Spearman correlation coefficients (r) and corresponding p-values are shown in each panel. No significant association between the decision boundary and payoff was observed in any group (see* ***Figure 4D*** *for decision boundary comparisons across groups).*

Supplementary Tables:

Table S12. Linear mixed-effects model predicting payoff from decision boundary α.

Fixed Effects	Coefficient	SE	df	*t*	*p*-value
(Intercept)	26.04	0.20	132.16	131.26	< .001
Medium α vs Low α	-0.32	0.07	242	-4.47	< .001
Medium α vs High α	0.22	0.07	242	3.08	0.002

Note. Payoff values obtained from model simulations in which the decision boundary parameter (α) was systematically varied across three levels (low, medium, high), while all other parameters were held constant at their fitted values.

R1.4. Discussion / introduction: It would be good to discuss the current results a bit more with reference to …

R1.4.a. … other lesion studies that address overall cognitive deficits (or their absence)
R1.4.b. … possible compensation "mechanisms" via the short- or long-term recruitment of other brain regions (possibly via plastic changes after the lesion).

Response: Thank you for this helpful suggestion. We have expanded the Introduction and the Discussion to place the findings within the broader lesion literature and to consider potential compensatory processes:

Introduction:
(…). Finally, whereas fMRI studies give relatively high resolution as to which areas of the mPFC are associated with ToM, existing lesion studies have relied on group comparisons with limited ability to identify mPFC subregions that are involved or to disentangle ToM-specific impairments from more general lesion-related effects on cognition[41,42]. New developments in lesion-symptom mapping[43] that can be applied to

larger samples of patients may therefore be necessary to determine whether there are specific regions causally implicated in ToM.

Discussion:
These findings should be interpreted within a multifaceted view of mentalising[78], rather than as evidence that the mPFC is unnecessary for all forms of social cognition. Contemporary accounts increasingly emphasise that mentalising comprises dissociable components—such as belief-based strategic reasoning, affective understanding, valuation of others, and self-referential processing—that may rely on partially distinct neural substrates[79–82]. The present paradigm specifically targets belief-based inference about another agent's likely actions during repeated strategic interaction, a component that may be supported by a distributed social cognition network rather than by the mPFC alone[83–85]. From this perspective, preserved performance following mPFC damage may reflect compensation via other regions within this network, either through long-term plastic reorganisation after the lesion or through the flexible recruitment of intact regions during task performance[86]. This aligns with some studies suggesting that a distributed network, including the temporoparietal junction (TPJ)[22–30], flexibly supports mental state attribution. (…).

R1.5. Minor: I find it helpful to link all supplementary figures to the relevant main figures and vice versa.

Response: Thank you for this helpful suggestion. We have revised the manuscript to ensure that all supplementary figures are clearly linked to the relevant main figures, and vice versa:

Figure 1 legend:
*We therefore analysed all results with and without these patients in the lesion control group, (…) (see **Supplementary Results**, **Figure S12** and **Table S13**). (…) (see **Supplementary Results**, **Figure S13**, and **Table S14**).*

Figure 2 legend:
*For experimental control, (…) (see **Methods** for details and **Figure S1** for role-assignment procedure). (…). See **Figure S2** & **S3** for the accumulated payoff and RTs distribution; see **Table S2-S10** for additional control and robustness analyses.*

Figure S1 legend:
*(…) (see **Figure 2A** for the game design).*

Figure S2 legend:
*(…) (see **Figure 2C** for the main payoff analysis).*

Figure S3 legend:
*(…) (see **Figure 2D** for the main RT analysis).*

Figure 3 legend:
*(B) The winning model showed excellent parameter recovery, as evidenced by the high correlations on the diagonal and the low correlations off the diagonal (see **Supplementary Results** and **Figure S5** for model validation). (C) BIC difference of each group (see **Figure S4** for the plot with individual data points overlaid; see **Figure S6** for additional predictive accuracy comparisons within the LC group). Also see*

Figure S7 *for illustrative trial sequence examples and* ***Figure S8*** *for parameters correlations matrix.*

Figure S4 legend:
Model fitting BIC across groups showed that the best fit was the influence-DDM model (see also ***Figure 3A & 3C****), for both the healthy control group and the mPFC lesion group.*

Figure S5 legend:
(D) the response times (RTs) (see ***Figure 3B*** *for parameter recovery results).*

Figure S6 legend:
(…) (See ***Figure 3C & S4*** *for the BIC difference of each group).*

Figure S7 legend:
(…). based on model-derived parameter estimates (see ***Figure 3*** *for main model fitting results).*

Figure S8 legend:
Heatmap showing pairwise Spearman correlations between the five model parameters (…). (see ***Figure 3*** *for main model fitting results).*

Figure 4 legend:
(D) The mPFC patients had a higher decision boundary than healthy controls, with anecdotal evidence for a difference from lesion controls (see ***Figure S9*** *for the other two DDM parameters,* ***Figure S10*** *for analysis of decision boundary and payoff,* ***Figure S11*** *for exploratory analysis, and* ***Figure S12-13*** *for control analyses).*

Figure S9 legend:
(B) The non-decision time τ was comparable between groups (see ***Figure 4*** *for main interest parameters).*

Figure S10 legend:
(…) (see ***Figure 4D*** *for decision boundary comparisons across groups).*

Figure S12 & S13 legend:
(…). (see ***Figure 4*** *and* ***Figure S4*** *for main analyses).*

Figure 5 legend:
(…). damage in the areas identified by the VLSM analysis (see ***Figure S15*** *for mPFC patients only analysis). (…). See* ***Figure S14*** *for lesion volume correlations.*

Figure S14 legend:
(…). (see ***Figure 5*** *for VLSM analysis results).*

Figure S15 legend:
damage in the areas identified by the mPFC-only VLSM analysis (see ***Figure 5*** *for whole brain analysis).*

Reviewer #2 (Remarks to the Author):

This article presents a novel and sophisticated study with valuable insights into mentalizing capacities in patients with lesions in the medial prefrontal cortex (mPFC). The authors have performed and described the lesion location as well as behavioral modeling in a very comprehensive manner, which is a significant strength of the manuscript. The core finding—that mPFC damage impacts the speed rather than the capacity for mentalizing during social interactions—offers a nuanced understanding of the mPFC's role in social cognition and is a critical contribution to the field. The use of a computational model combining learning and drift-diffusion processes to dissociate various underlying mechanisms is particularly commendable, as is the consistency between behavioral findings and self-reports of mentalizing strategy use in mPFC patients. This methodological rigor, employing state-of-the-art computational modeling and voxel-based lesion-symptom mapping (VLSM) on a valuable cohort of patients with focal brain lesions, represents a significant advancement.

However, despite these strengths, several aspects of the manuscript could be clarified or strengthened.

Response: Thank you very much for your thoughtful and constructive evaluation of our manuscript. We greatly appreciate your positive assessment of the methodological rigour and contribution of our work. Your comments have helped us to further clarify our analyses and strengthen the presentation of our findings, improving the overall clarity and impact of the manuscript.

R2.1. The introduction, for instance, dedicates a significant portion to summarizing the study, which feels repetitive after reading the abstract. This space could be more effectively utilized to provide a more comprehensive theoretical background on the general consequences of focal brain lesions, or to offer a deeper justification for the specific choices made regarding lesion and control groups in the context of prior mentalizing research.

Response: Thank you for this helpful suggestion. We have revised the introduction (please see also **R2.3** response to other introduction revisions).

R2.2. A central point of concern revolves around the sample size and how it is presented. While the mPFC lesion sample of N=33 is indeed remarkably large for a patient study, the manuscript frequently describes it as "large" and combines it with the "other lesion" control group (N=16) to form an even larger sample (33+16) in certain analyses. The primary value of this paper lies in the homogeneity and focal nature of the mPFC lesion sample, and combining it with a heterogeneous "other lesion" group in analyses where precise lesion localization is paramount risks diluting the findings. This practice appears to undermine the very advantage gained by focusing on specific focal lesions and may artificially inflate statistical power rather than genuinely enhancing the reliability or specificity of the results. This issue becomes particularly salient for the subregion analyses (VLSM). To truly identify mPFC subregions causally implicated in the observed effects, this analysis should ideally be restricted solely to the focal mPFC lesion group. Including the "other lesion" sample, which by definition should not have mPFC lesions, cannot logically contribute to finding specific mPFC subregions and merely serves to increase the statistical N, potentially masking true regional effects within the mPFC itself.

Response: Thank you for the opportunity to clarify. In response to this comment, we have revised the manuscript to more clearly explain our lesion-mapping strategy, and we have added an additional analysis focused on focal mPFC lesions.

We retain the whole-brain VLSM, including all lesion patients, as the primary analysis. This approach tests whether damage to specific voxels explains behavioural variability across

patients, over and above the general effects of having a brain lesion. To further increase stringency, we have now unified the previous analysis sets into a single analysis and applied a more conservative correction threshold ($p < 0.01$), correcting across all five parameters simultaneously.

Following the reviewer's suggestion, we have added an additional VLSM analysis restricted to the focal mPFC lesion group. This mPFC-only analysis is now presented in the Supplementary Materials and is explicitly framed as a complementary analysis examining subregional variability within the mPFC. Results from this analysis are reported separately and interpreted cautiously.

We have updated the Methods and Supplementary Materials to clearly distinguish the goals of the all-lesion patients and mPFC-only VLSM analyses. We have also revised the Results to reflect the unified and more stringent thresholding strategy and updated the Discussion accordingly. These changes are described in detail below:

> ***Introduction:***
> *(…). New developments in lesion-symptom mapping[43] that can be applied to larger samples of patients may therefore be necessary to determine whether there are specific regions causally implicated in ToM.*
>
> ***Results:***
> ***Damage to mPFC subregions impacts on boundary, drift rate temperature, and non-decision time***
> *Our behavioural analysis showed that mPFC damage was in general associated with preserved mentalising but slower decision-making during strategic interactions, accompanied by higher boundaries in the evidence accumulation process. We next sought to examine whether there were specific regions in the brain where damage was associated with more fine-grained differences in the extent of influence play, fictitious play, or the DDM choice parameters. Given our relatively large sample of brain-lesion patients (N = 49), we employed voxel-based lesion-symptom mapping (VLSM), which tests whether voxel-level damage predicts variability in model-derived parameters across patients, by generating a brain t-statistics map that is independent of group-level differences. The VLSM was conducted using the permutation-based threshold-free cluster enhancement (TFCE)[63,64] implemented in the FMRIB Software Library (FSL) (see* ***Methods*** *and* ***Supplementary Experimental Procedures****).*
>
> *In this whole-brain analysis, we included all lesion patients (the mPFC lesion group and the lesion control group). This approach tests whether damage to a given voxel accounts for parameter variability beyond the general effects of a brain lesion. To ensure stable voxel-wise estimates, voxels damaged in fewer than five patients were classified as intact rather than lesioned. The analysis, therefore, provides a conservative assessment of voxel-level lesion–behaviour associations across the brain. Using a single unified analysis across all five model parameters (significance was reported at permutation-based TFCE $p < 0.01$, Bonferroni-corrected across five regressors), we first examined strategy-related parameters. Neither the influence play*

nor the fictitious play parameters were significantly correlated with any areas of damage.

*We next examined decision-related DDM parameters. We observed that damage to specific mPFC subregions was associated with a higher boundary α. Lesions involving the ventral anterior cingulate cortex (vACC) compared to damage elsewhere showed this pattern (**Figure 5A-5B**; peak MNI coordinate [10, 24, -4], cluster size k = 2). Damage to the orbitofrontal cortex (OFC), extending to anterior prefrontal cortex (aPFC), resulted in a higher drift rate parameter d compared to damage elsewhere (**Figure 5C-5D**; peak MNI coordinate [10, 62, 7], cluster size k = 1700; peak MNI coordinate [1, 36, -14], cluster size k = 526; peak MNI coordinate [2, 36, -27], cluster size k = 526). Finally, participants with lesions in the vACC also displayed a higher non-decision time τ (**Figure 5E-5F;** peak MNI coordinate [4, 32, -1], cluster size k = 42).*

*To further examine whether variability within the mPFC itself was associated with these parameters, we conducted a complementary VLSM analysis restricted to the focal mPFC lesion group. This mPFC-only analysis tests whether differences in lesion location within the mPFC predict parameter variability, independent of damage outside this region. Results and the discussion from this within-region analysis are presented in the **Supplementary Results** and **Figure S15**.*

Together, these analyses indicate that while higher-order mentalising parameter (influence-play) was not associated with damage to specific mPFC regions, variability in decision-related parameters was linked to damage within the mPFC regions, as revealed by both whole-brain and within-region lesion-mapping approaches. These findings support the mPFC areas as crucial for the latent factors influencing the decision process in general and response times specifically during strategic interactions.

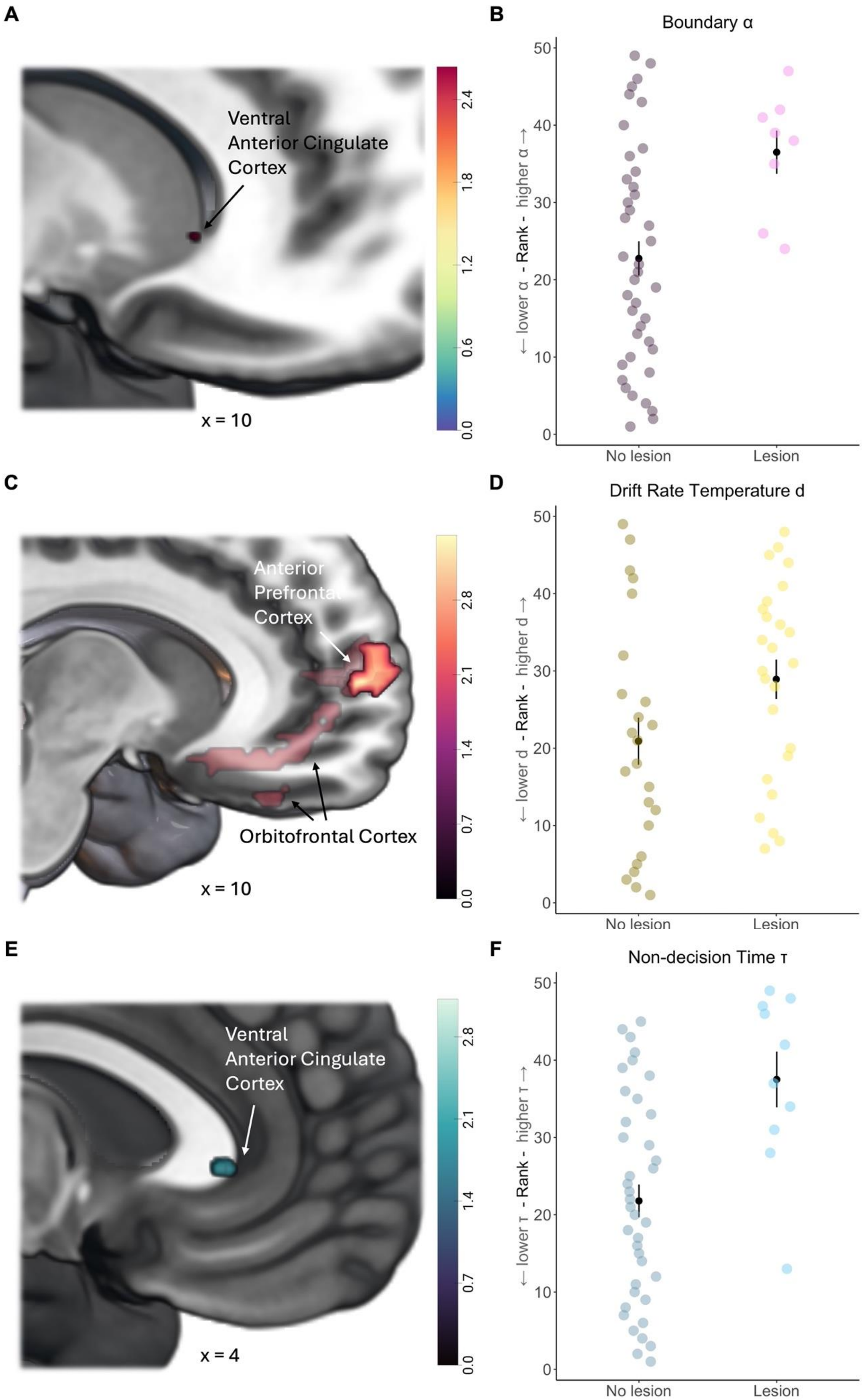
A
Ventral
Anterior Cingulate
Cortex
x = 10
0.0
0.6
1.2
1.8
2.4
B
Boundary α
← lower α - Rank - higher α →
No lesion
Lesion
C
Anterior
Prefrontal
Cortex
Orbitofrontal Cortex
x = 10
0.0
0.7
1.4
2.1
2.8
D
Drift Rate Temperature d
← lower d - Rank - higher d →
No lesion
Lesion
E
Ventral
Anterior Cingulate
Cortex
x = 4
0.0
0.7
1.4
2.1
2.8
F
Non-decision Time τ
← lower τ - Rank - higher τ →
No lesion
Lesion

Figure 5. Damage to the orbitofrontal cortex (OFC), anterior prefrontal cortex (aPFC), and ventral anterior cingulate cortex (vACC) was associated with the DDM parameters. *Permutation-based, whole-brain, non-parametric voxel-based lesion-symptom mapping (VLSM) results of (A) decision boundary α, (C) drift rate temperature d, and (E) non-decision time τ. The ranked (B) boundary α, (D) temperature d, and (F) non-decision time τ contrasts of participants with damage or no damage in the areas identified by the VLSM analysis (see **Figure S15** for mPFC patients only analysis). $p < 0.01$ (the colour bar represents the t-value of the analysis). Note: Panels (B), (D), and (F) are for illustrative purposes only and display the ranked difference in model parameter contrasts between participants with vs. without lesions in the ROI defined by a whole-brain contrast (see **Figure S14** for lesion volume correlations).*

Materials and Methods:

Voxel-based lesion-symptom mapping (VLSM)

For each participant, parameters were ranked to rectify the skewness of the residual distribution and subsequently z-scored[64]. Separate tests were conducted for positive and negative ranks to evaluate these hypotheses. Rankings were input into the FMRIB software library (FSL v6.0.7.15)[136], and permutation-based VLSM analyses were performed using the randomise function to compare each damaged voxel across all patients (mPFC lesion and lesion controls)[64]. For the primary analysis, we performed a whole-brain VLSM including all lesion patients (mPFC lesion group and lesion control group). This approach provides a conservative test of lesion–behaviour associations by leveraging heterogeneous lesion coverage across the brain while controlling for nonspecific lesion effects. Voxels were only included if at least five patients had that voxel damaged to some extent[137]. Lesion maps were mirrored to increase power, given no prior lateralisation hypothesis.

(…).

*In addition to the whole-brain analysis, we conducted a supplementary VLSM restricted to the focal mPFC lesion group to examine subregional variability within the mPFC. This mPFC-only analysis is reported in the **Supplementary Materials** and is interpreted as a complementary within-region analysis.*

Supplementary Results:

Restricted the VLSM analysis to the focal mPFC lesion group

To further examine whether variability in lesion location within the mPFC itself was associated with model-derived results, we conducted an additional VLSM analysis restricted to the focal mPFC lesion group. This analysis specifically tests whether differences in damage distribution within the mPFC predict variability in model-derived parameters, independent of lesions outside this region.

Using the same preprocessing and statistical procedures as in the whole-brain analysis (rank transformation, permutation-based VLSM with TFCE), we examined associations between voxel-wise damage and all five model parameters within the mPFC lesion

group only. This analysis was restricted to voxels within the mPFC mask and therefore should be interpreted as a within-region analysis.

*Within the mPFC-only analysis, no significant associations were observed for the higher-order mentalising parameter, influence play κ. In contrast, the simpler strategy, first-order belief-updating, fictitious play parameter η showed a significant association with damage to orbitofrontal cortex (OFC) within the mPFC regions (**Figure S15A** and **Figure S15B**; $p < 0.01$; peak MNI coordinate [11, 24, -22], cluster size k = 245; peak MNI coordinate [10, 37, -25], cluster size k = 58). For decision-related DDM parameters, within the mPFC, damage to the orbitofrontal cortex (OFC) and extending to anterior prefrontal cortex (aPFC) was associated with higher drift rate temperature d(**Figure S15C** and **Figure S15D**; $p < 0.01$; peak MNI coordinate [9, 62, 6], cluster size k = 1,275; peak MNI coordinate [1, 41, -16], cluster size k = 1,192) and non-decision time τ (**Figure S15E** and **Figure S15F**; $p < 0.01$; peak MNI coordinate [2, 61, -1], cluster size k = 47), while no significant associations were observed for decision boundary α after stringent correction.*

Notably, an association between fictitious play and damage to lateral orbital regions emerged only in the mPFC-only VLSM analysis and not in the conservative whole-brain analysis. This pattern suggests that first-order belief updating may depend on more fine-grained subregional variation within the mPFC (Hampton et al., 2008), rather than reflecting a robust voxel-level effect detectable across heterogeneous lesion sites. Importantly, no associations were observed between any lesion region and the influence play parameter κ across either the whole-brain or mPFC-only analyses. This absence of lesion–symptom mapping effects for influence play κ is consistent with the behavioural findings of preserved higher-order mentalising following mPFC damage and suggests that social strategic reasoning can be maintained despite focal disruption to mPFC subregions.

In contrast, associations between decision boundary and mPFC damage were observed in the whole-brain VLSM analysis but were not consistently detected in the mPFC-only analysis. This difference likely reflects the distinct goals of the two approaches. The whole-brain analysis provides a conservative test of voxel-level lesion–behaviour associations beyond the general effects of having a brain lesion, whereas the mPFC-only analysis examines relative variability within an already focal lesion group. Taken together, these findings support a robust role of mPFC damage in altered decision thresholds during strategic interactions, while suggesting that subregional variability within the mPFC may play a more limited role for this parameter.

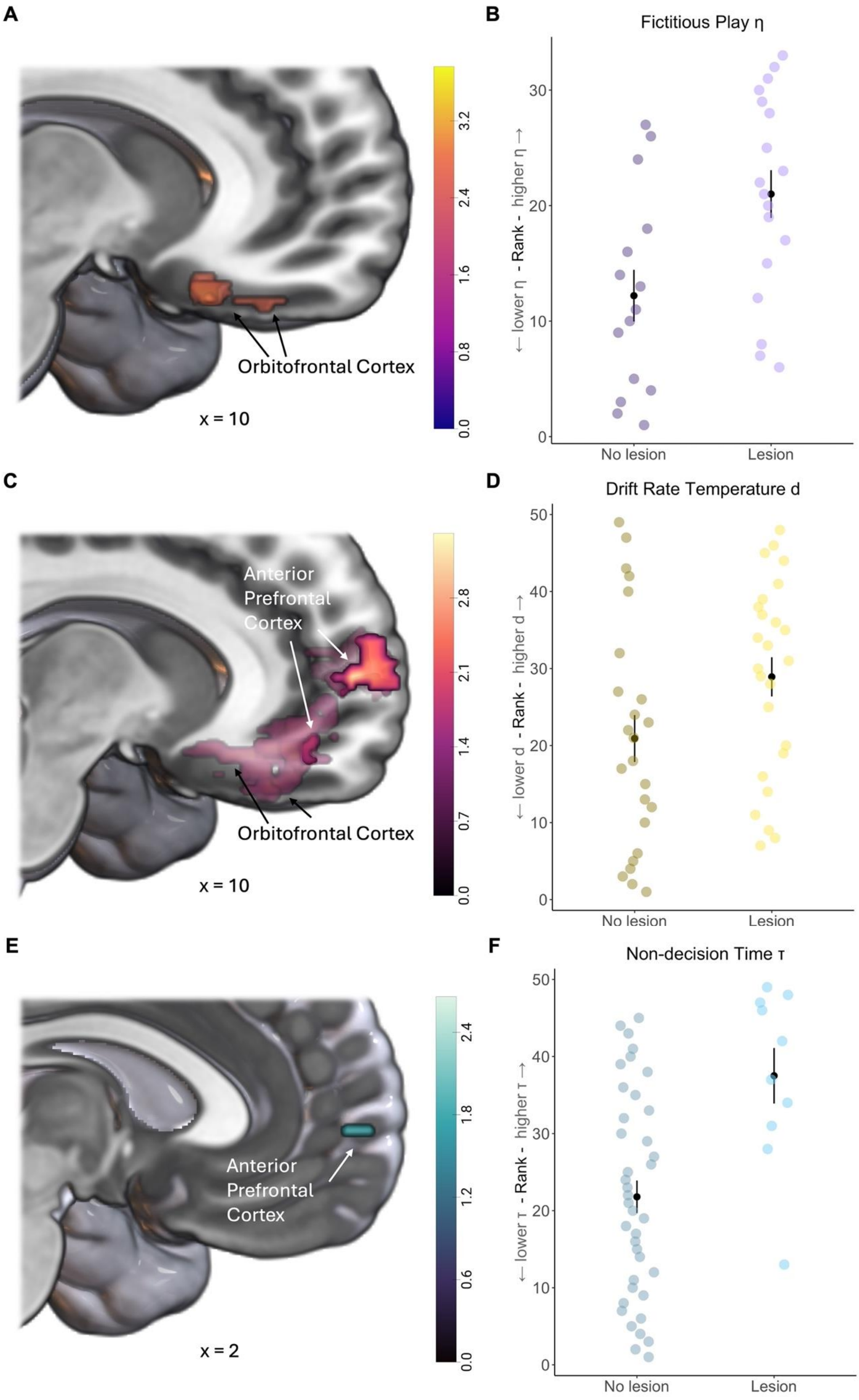

A
Orbitofrontal Cortex
x = 10
0.0
0.8
1.6
2.4
3.2
B
Fictitious Play η
← lower η - Rank - higher η →
0
10
20
30
No lesion
Lesion
C
Anterior Prefrontal Cortex
Orbitofrontal Cortex
x = 10
0.0
0.7
1.4
2.1
2.8
D
Drift Rate Temperature d
← lower d - Rank - higher d →
0
10
20
30
40
50
No lesion
Lesion
E
Anterior Prefrontal Cortex
x = 2
0.0
0.6
1.2
1.8
2.4
F
Non-decision Time τ
← lower τ - Rank - higher τ →
0
10
20
30
40
50
No lesion
Lesion

Figure S15. Damage to the orbitofrontal cortex (OFC) was associated with the first-order belief-updating, and damage to the orbitofrontal cortex (OFC) and anterior prefrontal cortex (aPFC) were associated with the DDM parameters. *Permutation-based, within-mPFC, non-parametric voxel-based lesion-symptom mapping (VLSM) results of (A) fictitious play η, (C) drift rate temperature d, and (E) non-decision time τ. The ranked (B) fictitious play η, (D) drift rate temperature d, and (F) non-decision time τ contrasts of participants with or without damage in the areas identified by the mPFC-only VLSM analysis (see* ***Figure 5*** *for whole brain analysis). p < 0.01 (the colour bar represents the t-value of the analysis). Note: Panels (B), (D), and (F) are for illustrative purposes only and display the ranked difference in model parameter contrasts between participants with vs. without lesions in the ROI defined by a whole-brain contrast.*

R2.3. Furthermore, the choice and characterization of the lesion control group (N=16) are somewhat unclear. This group is rather small and appears to have a heterogeneous lesion profile, concentrated predominantly in right-lateralized temporal cortex regions as suggested by Figure 1B. It is challenging to argue that this group effectively serves as a general control for the "presence of a lesion" without risking confounding factors related to the specific brain regions affected. This raises questions about its added value over the healthy control group, particularly in distinguishing effects specific to mPFC damage from more general lesion-related deficits or from effects related to specific non-mPFC lesion sites. A more detailed justification for its specific utility as a control for mPFC lesions is needed.

Response: Thank you for the opportunity to clarify. The lesion control group is essential to control for the impact of experiencing a brain lesion (and the ensuing medical treatment)) compared to healthy controls. Lesions were intentionally chosen to be heterogeneous for scientific and practical reasons in the lesion control group.

Although lesions in this group are more frequently right-lateralised, inspection of individual lesion maps indicates limited spatial overlap, such that no more than four individuals shared damage in the same region (Figure 1B). We therefore do not interpret this group as indexing effects of any specific non-mPFC region. In response to this comment, we have expanded the justification of the lesion control group in both the Introduction and the Method:

Introduction:

(…). We compared performance on this task in the mPFC damage group to two closely matched control groups, a large sample of healthy age- and gender-matched controls (N=73) and a sample of patients who also had lesions that were outside of the mPFC (N=16, ***Figure 1B****). The lesion control group was included to control for the general consequences of experiencing a focal brain lesion and the ensuing medical treatment, while avoiding systematic damage to any single non-mPFC region[57]. (…).*

Methods:

Participants

The lesion control (LC) group was included to control for the general consequences of experiencing brain lesions, beyond comparisons with healthy individuals, while avoiding systematic damage to the mPFC. Lesions in this group were intentionally heterogeneous for both scientific and practical reasons. Although lesions in the LC group were more frequently right-lateralised, inspection of individual lesion maps revealed limited spatial overlap, such that no more than four individuals shared

*damage in the same region (**Figure 1B**). Accordingly, this group was not intended to index effects of any specific non-mPFC region, but rather to provide a control for lesion-related factors that are not specific to mPFC damage.*

R2.4. Regarding the assessment of "conserved cognitive abilities," the manuscript primarily mentions the Trail Making Test Part A (TMT-A) as a control measure. While TMT-A assesses visual attention, it is not a comprehensive measure of general cognitive abilities and does not cover crucial domains such as memory, logical reasoning, or language. For a study focusing on mentalizing, having a broader battery of non-social cognitive reasoning measures would be vital to ensure that any observed preserved abilities or deficits are indeed socially specific and not simply attributable to more general cognitive impairments or preservation. The claims of preserved performance would be substantially strengthened if other cognitive domains were explicitly controlled for.

Response: Thank you for the opportunity to clarify. Participants also completed broader neuropsychological assessments, including the Raven Standard Progressive Matrices and the Addenbrooke's Cognitive Examination, which index reasoning ability and global cognitive functioning across multiple domains. We now explicitly refer to these measures in the main text and report group differences in the revised manuscript. Moreover, we include Raven and Trail Making Test scores as additional control variables in the analyses to clarify that claims of preserved performance but altered decision dynamics are specific to strategic social reasoning in our task:

Results:
Patients with brain damage demonstrate preserved performance but slower response times in strategic social interactions
*(…). Therefore, although mPFC damage was associated with slower response times, performance remained intact. This pattern remained unchanged when controlling for individual differences in general cognitive ability and non-social processing speed, as indexed by Raven Standard Progressive Matrices and Trail Making Test performance (see **Supplementary Results**, **Table S9-S10**). (…).*

Materials and Methods:
Participants
*The final sample included 122 participants (…). In addition, the mPFC patients did not differ significantly from the healthy or lesion control groups in the neuropsychological test assessing visual attention and processing speed (Trail Making Test; Part A ps > 0.62, Part B ps > 0.12). Participants also completed additional neuropsychological assessments indexing general cognitive ability, including the Raven Standard Progressive Matrices (RSPM) and the Addenbrooke's Cognitive Examination (ACE-III). Group differences were observed on these measures, with lesion patients scoring lower than healthy controls, consistent with broader cognitive consequences of focal brain injury (see **Supplementary Results** and **Table S1**). Importantly, the two lesion groups were broadly comparable on these measures.*

Discussion:
Despite employing a validated interactive social task and a relatively large lesion sample, several limitations should be acknowledged. (…). Finally, the present approach reflects an inherent trade-off between experimental control and ecological richness. While the controlled and anonymised interaction enabled isolation of social strategic belief-based reasoning, and additional control analyses mitigate concerns

about general cognitive impairment, the task does not fully capture the richness of naturalistic social interaction, and standard neuropsychological measures do not provide trial-level non-social benchmarks for directly comparing decision dynamics. Even with these limitations, the present work represents a significant advance beyond traditional ToM paradigms[124–126] *by integrating a more grounded interactive task with computational modelling and lesion-symptom mapping to capture dynamic strategy-based social interactions that require mentalisation.*

Supplementary Results:

Neuropsychological measures and cognitive control analyses

Group comparisons on general cognitive measures showed that the mPFC lesion group scored lower than healthy controls on the Raven Standard Progressive Matrices (RSPM), while not differing significantly from the lesion control group. A similar pattern was observed for global cognitive functioning as assessed by the Addenbrooke's Cognitive Examination (ACE-III), which was administered to lesion patients and an age-matched subset of healthy controls (>40 years). In contrast, performance on the Trail Making Test (Parts A and B), which indexes visual attention and non-social processing speed, did not differ significantly between the three groups. This pattern is consistent with expectations in neurological samples and indicates that, while patients differed from healthy controls on some general cognitive measures, the two lesion groups were broadly matched, particularly on measures most relevant to response speed (see ***Table S1****).*

*To assess whether the observed group differences in response time (RT) could be explained by general cognitive ability or non-social processing speed, we conducted an additional control analysis including both RSPM scores and Trail Making Test performance as covariates in the RT models. The main effect of group on RT remained significant, and the pattern and magnitude of group differences were unchanged (****Table S9****). Inclusion of these covariates did not alter any other key findings: there were still no group differences in overall payoff (****Table S10****), and group comparisons of model-derived parameters yielded the same pattern of results as in the primary analyses (****Table S11****). Together, these results indicate that the slower decision-making observed in mPFC patients cannot be explained by differences in general cognitive ability or non-social processing speed alone, and that the main behavioural and computational conclusions remain robust after accounting for these factors.*

Table S9. Robust linear mixed-effects model predicting RT with cognitive covariates.

Parameters	Coefficient	SE	CI low	CI high	*t*	*p*-value
(Intercept)	0.74	0.08	0.69	0.90	9.43	<.001
Group (mPFC vs HC)	-0.10	0.05	-0.19	-0.01	-2.24	0.025
Group (mPFC vs LC)	-0.14	0.06	-0.27	-0.02	-2.28	0.023
Ravens	-0.02	0.01	-0.04	0.00	-2.33	0.020
Trail Making Part A	0.00	0.00	0.00	0.01	2.32	0.020
Trail Making Part B	0.00	0.00	0.00	0.00	1.26	0.209

Note. mPFC: mPFC lesion patients, HC: Healthy controls, LC: Lesion controls. Three observations were excluded due to missing data.

Table S10. Control analyses of group differences in mean payoff (ANCOVA).

**Effect**	**df**	**Sum Sq**	**Mean Sq**	***F***	***p*-value**
Group	2	8.4	4.210	0.532	0.589
Ravens	1	5.7	5.699	0.720	0.398
Trail Making Part A	1	27.2	27.170	3.434	0.067
Trail Making Part B	1	14.3	14.270	1.803	0.182
Residual	113	894.2	7.913		

Note. Three observations were excluded due to missing data.

Table S11. ANCOVA results for model-derived parameters controlling cognitive measurements.

**Effect**	**df**	**Sum Sq**	**Mean Sq**	***F***	***p*-value**
Decision boundary					
Group	2	0.844	0.422	3.227	0.043
Ravens	1	1.057	1.057	8.074	0.005
Trail Making Part A	1	1.053	1.053	8.047	0.005
Trail Making Part B	1	0.202	0.203	1.547	0.216
Residual	113	14.787	0.131		
Drift rate temperature					
Group	2	0.002	0.001	2.630	0.077
Ravens	1	0.002	0.002	4.905	0.030
Trail Making Part A	1	0.002	0.002	4.560	0.035
Trail Making Part B	1	0.000	0.000	0.016	0.900
Residual	113	0.052	0.000		
Non-decision time					
Group	2	0.012	0.006	0.520	0.596
Ravens	1	0.015	0.015	1.328	0.252
Trail Making Part A	1	0.015	0.014	1.397	0.240
Trail Making Part B	1	0.014	0.011	1.253	0.265
Residual	113	1.250			
Fictitious play					
Group	2	0.034	0.017	0.124	0.884
Ravens	1	0.018	0.018	0.132	0.717
Trail Making Part A	1	0.166	0.166	1.209	0.274
Trail Making Part B	1	0.576	0.576	4.198	0.043
Residual	113	15.492	0.137		
Influence play					
Group	2	0.018	0.001	0.058	0.944
Ravens	1	0.071	0.071	0.455	0.502
Trail Making Part A	1	0.059	0.059	0.375	0.542
Trail Making Part B	1	0.623	0.623	2.968	0.049

Residual	113	17.734	0.157

Note. Three observations were excluded due to missing data.

R2.5. Finally, concerns arise about the ecological validity of the experimental paradigm. The description of the "presentation" of the confederate as an anonymous, physically met "other player" who is actually an algorithm feels highly artificial and unnatural. This approach, while controlled, might differ significantly from more ecologically valid social interactions, such as video-mediated interactions.

Response: Thank you for raising this point and for the opportunity to clarify. Participants indeed experienced the task as a genuine social interaction. Prior to the task, participants physically met the other player and engaged in a brief interaction (i.e., waving), and during the experiment, they were led to believe that they were interacting with this person following a very detailed protocol. First, the experimenter instructed the participant that the game they would play would be with the anonymous other person they met at the beginning of the experimental session. The experimenter and participant went through the instructions, and the participant did the practice task, which they were told would just be randomly picked responses by a computer to get used to responding within the allocated time window, but the actual task would be with the other person. Once the practice was complete, the experimenter told the participant they were leaving the room to check if the other person was ready, and they intentionally left a delay to make it plausible that someone else needed to start at the same time. The beginning of the main task started with a screen saying, 'connecting to other player,' and there was again a delay before the connection was successful. Importantly, post-task debriefing questions did not indicate that participants suspected the opponent to be artificial or algorithmic. This suggests that, from the participants' perspective, the interaction was experienced as social. The use of a non-video-based interaction was a deliberate design choice to minimise prior impressions or biases associated with facial appearance or identity, thereby allowing us to isolate strategic belief-based reasoning. At the same time, we acknowledge that the current paradigm does not constitute fully naturalistic social interaction. We have added additional details in our method section about the social setup and additional details in our discussion:

> ***Methods:***
> ***Procedure***
> *Participants then read the 'inspection game' task instructions (…). They completed a short practice task to familiarise them with the required response time and were told that during this practice, responses were randomly chosen by a computer. The practice phase was not repeated, as task understanding was confirmed prior to its onset. After the practice phase, the experimenter left the room to check whether the other player was ready, with a brief delay introduced to reinforce the impression of synchronisation with another participant. The start of the main task was preceded by a screen indicating that the system was "connecting to the other player," followed by an additional delay before the game commenced. These steps were designed to create a credible social interaction context while maintaining anonymity and experimental control. Notably, no participants suspected the opponent to be artificial or algorithmic in the post-task debriefing. (…).*
>
> ***Discussion:***
> *Despite employing a validated interactive social task and a relatively large lesion sample, several limitations should be acknowledged. (…). Finally, the present*

approach reflects an inherent trade-off between experimental control and ecological richness. While the controlled and anonymised interaction enabled isolation of social strategic belief-based reasoning, and additional control analyses mitigate concerns about general cognitive impairment, the task does not fully capture the richness of naturalistic social interaction, and standard neuropsychological measures do not provide trial-level non-social benchmarks for directly comparing decision dynamics. Even with these limitations, the present work represents a significant advance beyond traditional ToM paradigms[124–126] by integrating a more grounded interactive task with computational modelling and lesion-symptom mapping to capture dynamic strategy-based social interactions that require mentalisation.

Supplementary Experimental Procedures:
Role assignment process
(…). In reality, all participants were consistently assigned the role of 'employee'. To reinforce the belief that another real participant was present while minimising identity-based biases, the participant and a confederate researcher (posing as another participant) were introduced under controlled conditions without direct visual contact. Specifically, the two were positioned on opposite sides of a door, allowing them to see two experimenters simultaneously while preventing face-to-face interaction. Both the participant and the confederate wore rubber gloves and were instructed to silently wave at each other during the introduction.

This brief controlled interaction served to create a believable yet depersonalised social context for the subsequent game. Participants were reminded that the inspection game would be played against the other participant they had met at the beginning of the session. Connection delays prior to the main task further reinforced the impression of real-time interaction. Post-task debriefing did not indicate that participants suspected the opponent to be artificial or algorithmic, suggesting that the interaction was experienced as social from the participants' perspective. The use of a non-video-based interaction was a deliberate design choice to minimise prior impressions or biases related to facial appearance or identity, thereby allowing strategic belief-based reasoning to be examined in a controlled social context.

R2.6. Moreover, despite its computational sophistication, the paradigm, in its simplicity, might be more accurately conceptualized as a prediction error paradigm or strategic learning task rather than a nuanced representation of complex "mentalizing" in its broader sense. Clarifying the precise aspects of mentalizing that the paradigm is intended to capture would be beneficial for interpreting the "preserved mentalizing" claims.

Response: Thank you for raising this point. We have clarified in the manuscript that our use of the term 'mentalising' refers specifically to this belief-based, strategic component, and we have revised the text accordingly to avoid overgeneralisation. The inspection game used in this study, adapted from Hampton et al. (2008) and Hill et al. (2017), has been extensively validated as a well-established approach for studying strategic belief inference in interactive contexts. This paradigm is explicitly designed to capture a specific, theoretically grounded component of mentalising: belief-based reasoning about another agent's likely actions during repeated strategic interaction. Importantly, the model space accounts for a strategy that follows a simple prediction error strategy, and we show that a reinforcement learning model cannot account for behaviour on the task as accurately as a model that indeed incorporates belief updating. In addition, we did ask participants after the task a series of questions to probe the strategy they used, ranging from one that purely followed prediction errors about successful choice options (I tried to choose the option that worked best in the past) to one that explicitly used mentalising (I tried to influence my opponent's choice). Comparing the extent

to which participants used these strategies showed that people were, on average, more likely to report using mentalising.

> ***Discussion:***
> *These findings should be interpreted within a multifaceted view of mentalising[78], rather than as evidence that the mPFC is unnecessary for all forms of social cognition. Contemporary accounts increasingly emphasise that mentalising comprises dissociable components—such as belief-based strategic reasoning, affective understanding, valuation of others, and self-referential processing—that may rely on partially distinct neural substrates[79–82]. The present paradigm specifically targets belief-based inference about another agent's likely actions during repeated strategic interaction, a component that may be supported by a distributed social cognition network rather than by the mPFC alone[83–85]. From this perspective, preserved performance following mPFC damage may reflect compensation via other regions within this network, either through long-term plastic reorganisation after the lesion or through the flexible recruitment of intact regions during task performance[86]. This aligns with some studies suggesting that a distributed network, including the temporoparietal junction (TPJ)[22–30], flexibly supports mental state attribution. (…).*

Despite these criticisms, this reviewer believes the study presents important new data and innovative analytical approaches to a challenging research question. Addressing the points raised, particularly by refining the control group comparisons, clarifying the precise scope of "mentalizing" within the chosen paradigm, and strengthening the cognitive control measures, would significantly enhance the manuscript's conclusions and overall impact for Nature Communications.

Response: Thank you very much again for your time, for your positive evaluation of our work, and for your constructive suggestions, which have greatly helped us strengthen the manuscript!

Reviewer #3 (Remarks to the Author):

I co-reviewed this manuscript with one of the reviewers who provided the listed reports. This is part of the Nature Communications initiative to facilitate training in peer review and to provide appropriate recognition for Early Career Researchers who co-review manuscripts.

Response: Thank you very much for taking the time to contribute to the review of our manuscript. We really appreciate your evaluation of our work.

Reviewer #4 (Remarks to the Author):

In the manuscript under review, Hsieh et al. examined whether strategic mentalization was altered or intact in a relatively large sample of patients with mPFC damage. Previous work in healthy adults suggests that the mPFC is involved in social computations, specifically strategic social reasoning and theory of mind. The authors compared strategic game play in a previously validated social coordination game, the inspection task, in mPFC lesion patients, lesion control patients, and matched healthy controls.

The strengths of this paper are 1) the large patient sample size, 2) the validated task, and 3) the computational model that allows the author to disentangle latest cognitive contributions to overall performance. Notably, the authors have made the data and code available on OSF, which definitely increases the value of the contribution. The OSF site could be improved by README files in each folder that explain the data columns in each csv and .R file.

Response: Thank you for this helpful suggestion. We have now added README files to the OSF repository in each folder, providing clear descriptions of the folder structure and the data contents.

The results of the study are compelling: the authors find no evidence of altered k-level reasoning in mPFC lesion patients compared to lesion and healthy controls. However, medial prefrontal damage was significantly related to increased boundaries (caution), drift rate (i.e., evidence accumulation/conflict), and non-decision time (perceptual and motor processing).

I have only minor comments and suggestions.

Response: Thank you very much for your thoughtful and positive evaluation of our manuscript. We sincerely appreciate your recognition of the study's strengths. Your comments and suggestions have been very helpful in further improving the clarity and presentation of the manuscript.

R4.1. Please show more of the behavioral data, in addition to overall reward and RT, and prior to the model fit. For example, it would be useful to illustrate a sequence of rounds compatible with high fictious play but low influence; versus a sequence of rounds well fit by high influence. I would also like to see a plot of cumulative reward over rounds for each group, rather than just the overall average sum.

Response: Thank you for this helpful suggestion. We have now plotted example sequences of rounds and cumulative payoff over rounds, which are included in the **Supplementary Figures** as qualitative illustrations. Meanwhile, the main text intentionally focuses on summary behavioural measures and computational analyses that directly test the underlying decision-making mechanisms, which provide a quantitative basis for group-level inference.

> ***Results:***
> ***Patients with brain damage demonstrate preserved performance but slower response times in strategic social interactions***
> *We first analysed whether the three groups differed in overall performance. We found that there was substantial evidence for no difference in total reward received between the three groups (**Figure 2C**; $F_{(2,119)} = 0.313$, $p = 0.732$, $BF_{01} = 7.98$; see cumulative payoff trajectories across rounds for each group in **Figure S2**).*

Separating mentalising from basic choice mechanisms and perceptual and motor speed using computational modelling

*The influence-DDM model was best able to explain behaviour across groups, showing the lowest Bayesian Information Criterion (BIC) score (mean BIC: RL-DDM = 307.42, Fictitious-DDM = 308.37, Influence-DDM = 306.28, Simple Influence-DDM = 311.27) (**Figure 3A**; see **Figure 3C** and **Figure S4** for across-groups BIC with and without individual data points; see also **Figure S7** for illustrative example trial sequences).*

Supplementary Figures:

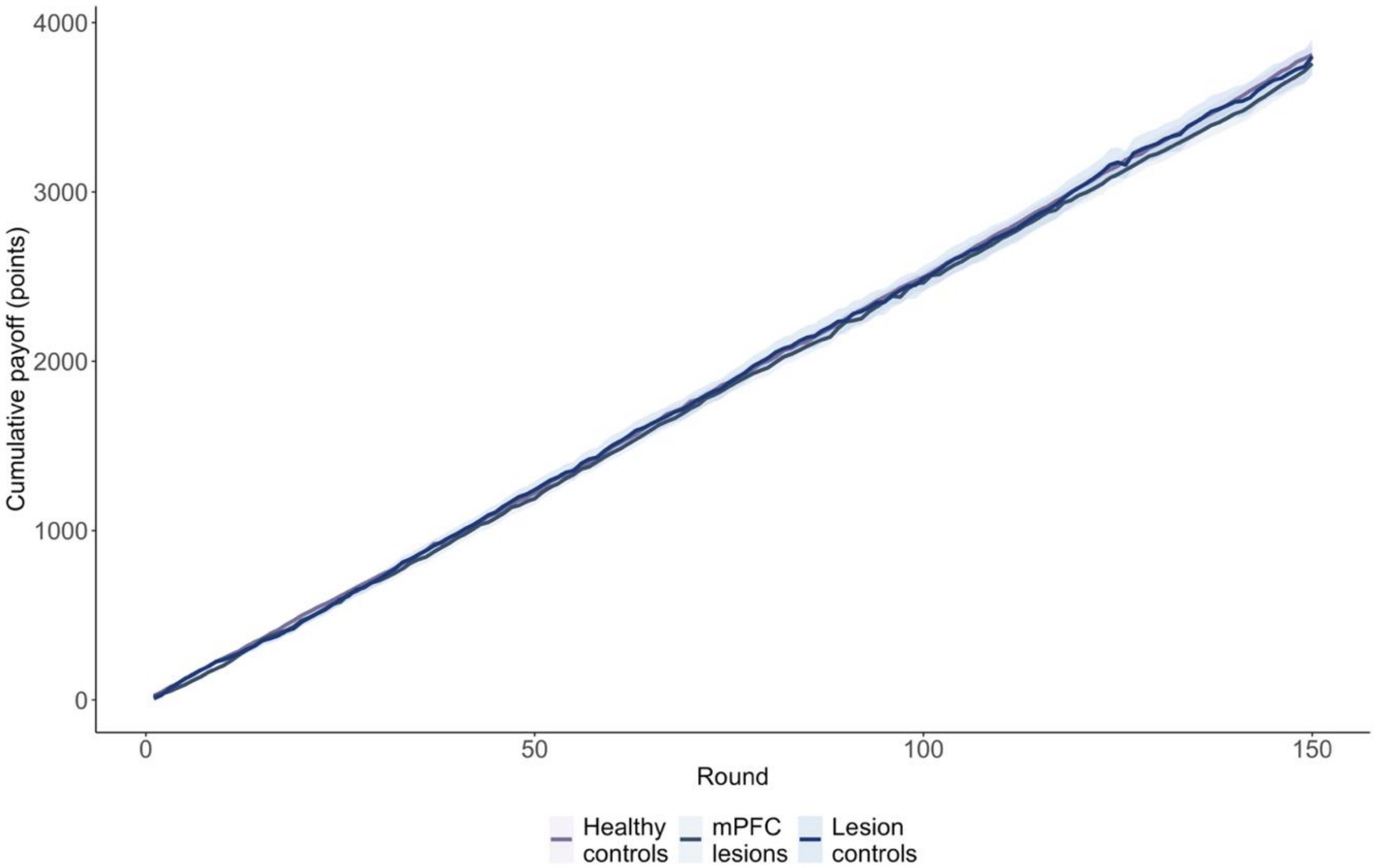

***Figure S2. Cumulative payoff over rounds for each group.** Mean cumulative payoff across rounds is shown separately for healthy controls, mPFC lesion patients, and lesion control participants. Shaded areas represent ±1 SEM. The trajectories show largely overlapping patterns across groups, consistent with the absence of group differences in overall payoff reported in the main text (see **Figure 2C** for the main payoff analysis).*

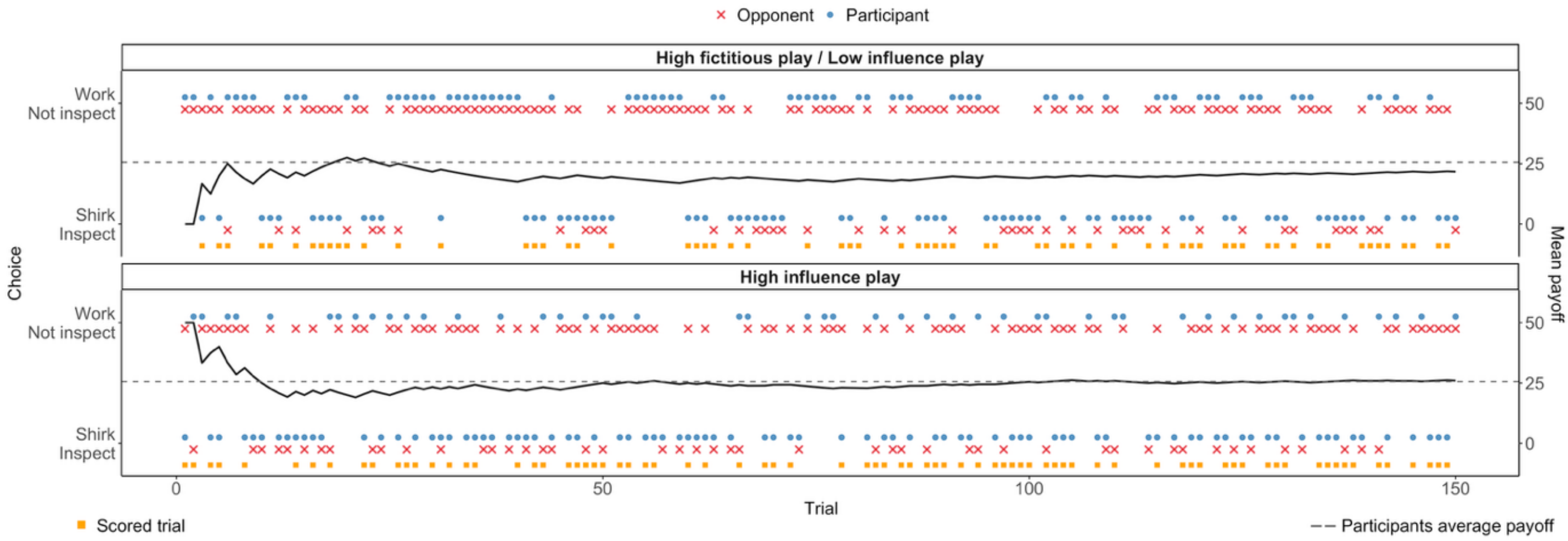

Figure S7. Illustrative example trial sequences showing different patterns of fictitious play and influence play of fitted influence-DDM. *Example sequences of rounds are shown for two healthy control participants whose behaviour was characterised by high fictitious play but low influence play (top panel) and by high influence play (bottom panel), based on model-derived parameter estimates (see **Figure 3** for main model fitting results).. These examples are provided for illustrative purposes to aid intuition about how different mentalising strategies unfold across rounds and are not intended for statistical comparison. Note that higher influence plays leads to more alternation in choices. The x-axis represents trial number, illustrating the temporal sequence of decisions across the task. For each trial, blue circles indicate the participant's choices and red crosses indicate the opponent's choices (work vs. shirk / inspect vs. not inspect, as labelled on the y-axis). Yellow squares indicate trials on which the participant received a payoff, providing a visual marker of rewarded outcomes. The black line represents the participant's running average payoff, while the dashed grey line indicates the mean payoff across participants.*

R4.2. The model comparison figure in the supplement, figure S2, does a great job of showing relative consistency in model performance across patient groups. I think this is a nicer plot to include in the main manuscript, in Fig. 3. instead of collapsed across groups. Including it in the main text may help convince readers that the influence parameters improve model performance across patient groups. Is it possible to include individual participants' data in the BIC plots, as points overlaid on the overall fits?

Response: Thank you for this helpful suggestion. We have revised the main manuscript to include the group-specific model comparison results, and in response to Reviewer 1.3.c, we have expanded the **Supplementary Materials** with further predictive accuracy analyses.
We have also considered overlaying individual participants' BIC values onto these plots; however, because the BIC differences are presented at the group-averaged level, including individual data points substantially reduced the readability of the figure and obscured the relative model comparisons. We therefore retained the current presentation in the main manuscript to preserve clarity and put the individual participant data version in supplementary materials:

Figures:

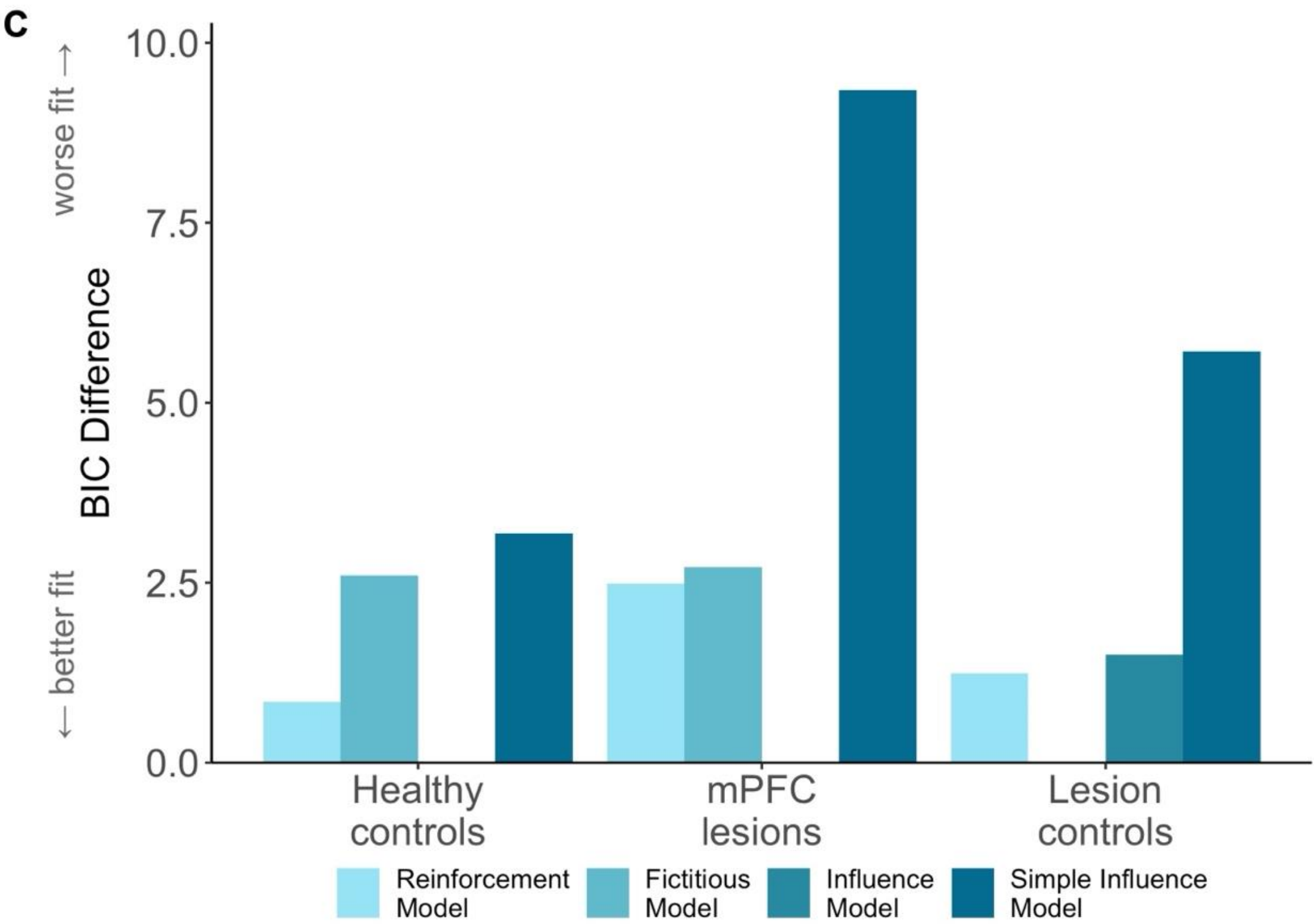

Figure 3. The influence-DDM model best accounts for participants' behaviour. *(…). (B) The winning model showed excellent parameter recovery, as evidenced by the high correlations on the diagonal and the low correlations off the diagonal (see **Supplementary Results** and **Figure S5** for model validation). (C) BIC difference of each group (see **Figure S4** for the plot with individual data points overlaid; see **Figure S6** for additional predictive accuracy comparisons within the LC group).*

Supplementary Figures:

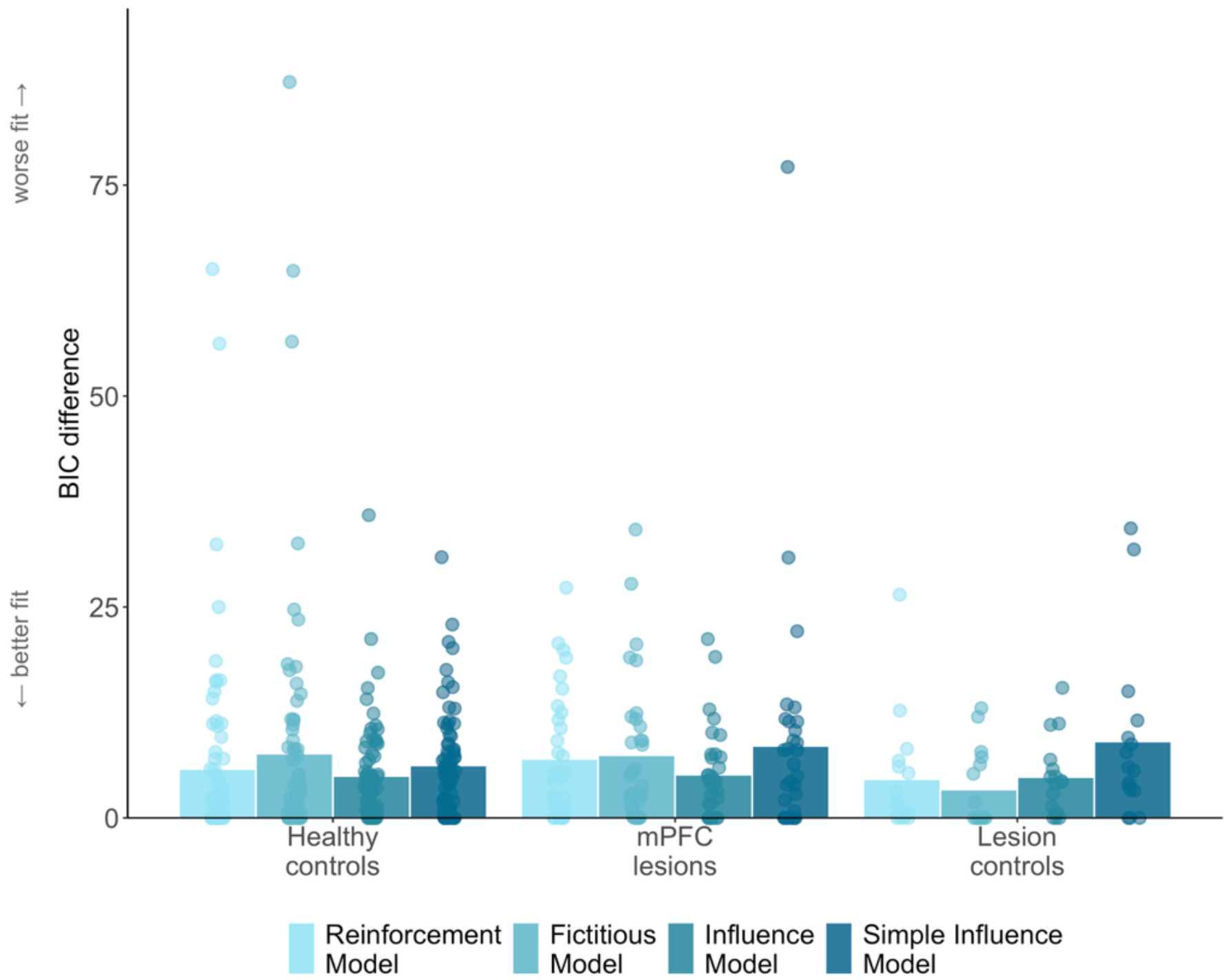

Figure S4. Relative BIC differences for model comparison across groups with individual data. *Model comparison using BIC across groups indicated that the influence-DDM model generally provided the best fit for both the healthy control group and the mPFC lesion group (see also **Figure 3A** & **3C**), whereas the fictitious play model tended to provide the best fit for the lesion control group. For each participant, relative BIC difference values were computed by subtracting the lowest BIC value among all candidate models from that participant's BIC values. Therefore, the model with the lowest BIC for a given participant was assigned a relative BIC difference of 0, and larger relative BIC differences indicated worse model fit. Because the best-fitting model could vary across individuals, the zero point in the relative BIC scale did not necessarily correspond to the same model across participants. The distinct pattern observed in the lesion control group might be related to its smaller sample size (N = 16).*

R4.3. The methods section includes the statement "three other experimental tasks were reported elsewhere." Please add citations, because these other data could be relevant for understanding the overall cognitive profile of these groups.

Response: Thank you for your suggestion. We agree and have updated the manuscript to include citations for the tasks reported elsewhere:

Materials and Methods:
Procedure
After the series of tasks was done (three other experimental tasks are reported elsewhere)[54–56]*, participants completed a range of questionnaires (see below and Supplementary Experimental Procedures).*

R4.4. It would also help to know, for reference, whether this patient group showed deficits on any other standard decision making task. If these patients are not impaired in anything, that would confuse the interpretation of the lack of impairment in k-level reasoning.

Response: Thank you for the opportunity to clarify. In addition to the inspection game, participants completed a battery of standardised assessments, including general cognitive functioning assessments, the Raven Standard Progressive Matrices and the Addenbrooke's Cognitive Examination. Group differences were observed on these measures, with lesion patients performing worse than the healthy control group. We further show that controlling for these measures does not alter the preserved performance in lesion groups and observed group differences in RT, supporting the specificity of the main findings. These analyses are now explicitly reported and discussed in the revised manuscript:

Results:
Patients with brain damage demonstrate preserved performance but slower response times in strategic social interactions
*(…). Therefore, although mPFC damage was associated with slower response times, performance remained intact. This pattern remained unchanged when controlling for individual differences in general cognitive ability and non-social processing speed, as indexed by Raven Standard Progressive Matrices and Trail Making Test performance (see **Supplementary Results, Table S9-S10**). (…).*

Discussion:
Despite employing a validated interactive social task and a relatively large lesion sample, several limitations should be acknowledged. (…). Finally, the present approach reflects an inherent trade-off between experimental control and ecological

richness. While the controlled and anonymised interaction enabled isolation of social strategic belief-based reasoning, and additional control analyses mitigate concerns about general cognitive impairment, the task does not fully capture the richness of naturalistic social interaction, and standard neuropsychological measures do not provide trial-level non-social benchmarks for directly comparing decision dynamics. Even with these limitations, the present work represents a significant advance beyond traditional ToM paradigms[124–126] by integrating a more grounded interactive task with computational modelling and lesion-symptom mapping to capture dynamic strategy-based social interactions that require mentalisation.

Materials and Methods:

Participants

*The final sample included 122 participants (…). In addition, the mPFC patients did not differ significantly from the healthy or lesion control groups in the neuropsychological test assessing visual attention and processing speed (Trail Making Test; Part A ps > 0.62, Part B ps > 0.12). Participants also completed additional neuropsychological assessments indexing general cognitive ability, including the Raven Standard Progressive Matrices (RSPM) and the Addenbrooke's Cognitive Examination (ACE-III). Group differences were observed on these measures, with lesion patients scoring lower than healthy controls, consistent with broader cognitive consequences of focal brain injury (see **Supplementary Results** and **Table S1**). Importantly, the two lesion groups were broadly comparable on these measures.*

Supplementary Results:

Neuropsychological measures and cognitive control analyses

*Group comparisons on general cognitive measures showed that the mPFC lesion group scored lower than healthy controls on the Raven Standard Progressive Matrices (RSPM), while not differing significantly from the lesion control group. A similar pattern was observed for global cognitive functioning as assessed by the Addenbrooke's Cognitive Examination (ACE-III), which was administered to lesion patients and an age-matched subset of healthy controls (>40 years). In contrast, performance on the Trail Making Test (Parts A and B), which indexes visual attention and non-social processing speed, did not differ significantly between the three groups. This pattern is consistent with expectations in neurological samples and indicates that, while patients differed from healthy controls on some general cognitive measures, the two lesion groups were broadly matched, particularly on measures most relevant to response speed (see **Table S1**).*

*To assess whether the observed group differences in response time (RT) could be explained by general cognitive ability or non-social processing speed, we conducted an additional control analysis including both RSPM scores and Trail Making Test performance as covariates in the RT models. The main effect of group on RT remained significant, and the pattern and magnitude of group differences were unchanged (**Table S9**). Inclusion of these covariates did not alter any other key findings: there were still no group differences in overall payoff (**Table S10**), and group comparisons of model-derived parameters yielded the same pattern of results as in the primary analyses (**Table S11**). Together, these results indicate that the slower decision-making observed in mPFC patients cannot be explained by differences in general cognitive ability or non-social processing speed alone, and that the main behavioural and computational conclusions remain robust after accounting for these factors.*

Table S9. Robust linear mixed-effects model predicting RT with cognitive covariates.

Parameters	Coefficient	SE	CI low	CI high	*t*	*p*-value
(Intercept)	0.74	0.08	0.69	0.90	9.43	<.001
Group (mPFC vs HC)	-0.10	0.05	-0.19	-0.01	-2.24	0.025
Group (mPFC vs LC)	-0.14	0.06	-0.27	-0.02	-2.28	0.023
Ravens	-0.02	0.01	-0.04	0.00	-2.33	0.020
Trail Making Part A	0.00	0.00	0.00	0.01	2.32	0.020
Trail Making Part B	0.00	0.00	0.00	0.00	1.26	0.209

Note. mPFC: mPFC lesion patients, HC: Healthy controls, LC: Lesion controls. Three observations were excluded due to missing data.

Table S10. Control analyses of group differences in mean payoff (ANCOVA).

Effect	df	Sum Sq	Mean Sq	*F*	*p*-value
Group	2	8.4	4.210	0.532	0.589
Ravens	1	5.7	5.699	0.720	0.398
Trail Making Part A	1	27.2	27.170	3.434	0.067
Trail Making Part B	1	14.3	14.270	1.803	0.182
Residual	113	894.2	7.913		

Note. Three observations were excluded due to missing data.

Table S11. ANCOVA results for model-derived parameters controlling cognitive measurements.

Effect	df	Sum Sq	Mean Sq	*F*	*p*-value
Decision boundary					
Group	2	0.844	0.422	3.227	0.043
Ravens	1	1.057	1.057	8.074	0.005
Trail Making Part A	1	1.053	1.053	8.047	0.005
Trail Making Part B	1	0.202	0.203	1.547	0.216
Residual	113	14.787	0.131		
Drift rate temperature					
Group	2	0.002	0.001	2.630	0.077
Ravens	1	0.002	0.002	4.905	0.030
Trail Making Part A	1	0.002	0.002	4.560	0.035
Trail Making Part B	1	0.000	0.000	0.016	0.900
Residual	113	0.052	0.000		
Non-decision time					
Group	2	0.012	0.006	0.520	0.596
Ravens	1	0.015	0.015	1.328	0.252
Trail Making Part A	1	0.015	0.014	1.397	0.240

Trail Making Part B	1	0.014	0.011	1.253	0.265
Residual	113	1.250			
Fictitious play					
Group	2	0.034	0.017	0.124	0.884
Ravens	1	0.018	0.018	0.132	0.717
Trail Making Part A	1	0.166	0.166	1.209	0.274
Trail Making Part B	1	0.576	0.576	4.198	0.043
Residual	113	15.492	0.137		
Influence play					
Group	2	0.018	0.001	0.058	0.944
Ravens	1	0.071	0.071	0.455	0.502
Trail Making Part A	1	0.059	0.059	0.375	0.542
Trail Making Part B	1	0.623	0.623	2.968	0.049
Residual	113	17.734	0.157		

Note. Three observations were excluded due to missing data.

R4.5. Were these analyses confirmatory or exploratory? These claims would be particularly strong if preregistered. If some part of the analysis was exploratory, or if other analyses were attempted in addition to those reported, that would be useful to know in interpreting these results.

Response: Thank you for your query. Unfortunately, the study was not pre-registered. However, the analyses reported here were hypothesis-driven rather than exploratory. The task, computational models, and analytic approach were selected a priori, based on the procedures in previous publications, to test clear predictions about whether mPFC damage affects mentalising and related decision processes. All analyses conducted are reported in the manuscript and Supplementary Materials, with no additional unreported analyses performed. We make this point explicit in the manuscript to clarify.

> ***Methods:***
> ***Statistical analysis***
> *All analyses reported were specified a priori based on the task design and theoretical framework, and no additional unreported analyses were conducted.*

R4.6. Finally, there is one matter of rhetoric. The authors name their finding “preserved mentalising”. While this broad generalization, treating mentalising as a single construct, is consistent with the vast majority of the prior literature, personally I think it’s unhelpful. Recent meta-analyses suggest that existing “mentalising” tasks do not have strong construct validity (Yeung et al 2024) fwiw. The k-level parameter of influence play in this game captures one narrow aspect of mentalising / theory of mind. Without in any way diminishing the importance of this result, the claim is too general. Prior fMRI evidence of MPFC involvement in social reasoning would not necessarily have identified k-level in the influence game as the most likely role of MPFC within mentalising. MPFC seems to be particularly recruited for aspects of mentalising connected to valence, value and self-relevance — understanding others’ emotions, valuing certain others, considering what others value in the self. It may be possible to engage in influence play in these games without relying on the aspects of mentalising that particularly require MPFC.

Response: Thank you for this thoughtful comment. We agree that mentalising is unlikely to be a single construct and contains multiple components, and that construct validity is crucial (Yeung et al 2024). The title simply provided a shorthand way to describe the task participants did and was influenced by other published work using the same task, which labels behaviour as mentalising or mentalisation (Hampton et al., 2008; Hill et al., 2017). We agree that greater specificity in our title and additional discussion of the multifaceted nature of mentalising would be helpful. We have revised our title to "Preserved higher-order mentalising during strategic interactions after medial prefrontal damage" and provide additional discussion:

> ***Discussion:***
> *These findings should be interpreted within a multifaceted view of mentalising[78], rather than as evidence that the mPFC is unnecessary for all forms of social cognition. Contemporary accounts increasingly emphasise that mentalising comprises dissociable components—such as belief-based strategic reasoning, affective understanding, valuation of others, and self-referential processing—that may rely on partially distinct neural substrates[79–82]. The present paradigm specifically targets belief-based inference about another agent's likely actions during repeated strategic interaction, a component that may be supported by a distributed social cognition network rather than by the mPFC alone[83–85]. From this perspective, preserved performance following mPFC damage may reflect compensation via other regions within this network, either through long-term plastic reorganisation after the lesion or through the flexible recruitment of intact regions during task performance[86]. This aligns with some studies suggesting that a distributed network, including the temporoparietal junction (TPJ)[22–30], flexibly supports mental state attribution. (…).*

Reviewer #4 (Remarks on code availability):

R4.7. The OSF site could be improved by **README files** in each folder that explain the data columns in each csv and .R file.

Response: Thank you for this helpful suggestion. We have now added README files to the OSF repository in each folder, providing clear descriptions of the folder structure and the data contents.

REVIEWER COMMENTS

Reviewer #1 (Remarks to the Author):

I would like to thank the authors for addressing all my comments. Congratulations to this fine piece of work, which will have a considerably influence on the literature.

Response: Thank you for your positive assessment and encouraging comments.

Reviewer #2 (Remarks to the Author):

The authors have done a fantastic job in revising this manuscript and I just want to mention one further point.

Response: Thank you for your positive comments and helpful additional suggestions.

Thank you for elaborating on the tests done to probe general cognitive deficits in the participants. Given how crucial language capabilities are, I would still think it would have been important to also include a test there and would encourage the authors to mention this in the limitations.

Response: Thank you for highlighting the importance of considering language-related cognitive functions. In response, we have added a statement to the limitations section:

> ***Discussion***
> *(…). While the controlled and anonymised interaction enabled isolation of social strategic belief-based reasoning and additional control analyses mitigate concerns about general cognitive impairment, the absence of a formal language assessment means that language-related factors cannot be fully excluded. In addition, the task does not fully capture the richness of naturalistic social interaction, and standard neuropsychological measures do not provide trial-level non-social benchmarks for directly comparing decision dynamics. (…).*

Reviewer #3 (Remarks to the Author):

I co-reviewed this manuscript with one of the reviewers who provided the listed reports. This is part of the Nature Communications initiative to facilitate training in peer review and to provide appropriate recognition for Early Career Researchers who co-review manuscripts.

Response: Thank you for your contribution to the peer-review process.

Reviewer #4 (Remarks to the Author):

I am satisfied that the authors have addressed my review and the suggestions and questions of the author reviewers, with their extensive thoughtful responses. I recommend publication.

Response: Thank you for your positive evaluation and support for publication.
